# Green Synthesis of 2-Mercapto 5,6-Dihydro-4*H*-1,3-Thiazines via Sequential C–S Couplings

**DOI:** 10.3390/molecules29225255

**Published:** 2024-11-06

**Authors:** Wenjie Liu, Shuo Wang, Li Pan, Xiaojing Bi, Enxue Shi

**Affiliations:** 1 State Key Laboratory of NBC Protection for Civilian, Beijing 102205, China; l2507338854@163.com (W.L.); ws20140506@163.com (S.W.); bk6180b@163.com (L.P.); 2 School of Chemistry and Environmental Engineering, Wuhan Institute of Technology, Wuhan 430205, China

**Keywords:** 5,6-dihydro-4*H*-1,3-thiazines, thiol click reaction, cascade C–S coupling, microwave-assisted, green synthesis, DFT calculation

## Abstract

The six-membered N,S-heterocyclic 1,3-thiazines and their derivatives are widely acknowledged as pharmaceutical molecules with a wide range of biological activities. In this study, we developed a unique thiol-involved cascade reaction that enables the efficient construction of the 5,6-dihydro-4*H*-1,3-thiazine scaffold through consecutive intermolecular thiol-isothiocyanate and intramolecular thiol-halogen click reactions. Structurally diverse 2-mercapto dihydrothiazines including three antitumour candidates of *bis*-dihydrothiazines were readily obtained in high yields from the readily available thiols and 3-chloroisothiocyanate in the green solvent EtOH/H_2_O (1:1) using K_2_CO_3_ (0.6 equiv.) as the base. Between the two synthesis procedures investigated, the microwave-assisted reaction generally behaved more efficiently than that under routine heating conditions. Furthermore, DFT calculation confirmed the sequential addition–substitution mechanism. This cascade C–S coupling reaction methodology offers several advantages, including rapid completion, high reliability, easy purification, and benign conditions.

## 1. Introduction

**Figure 1:**
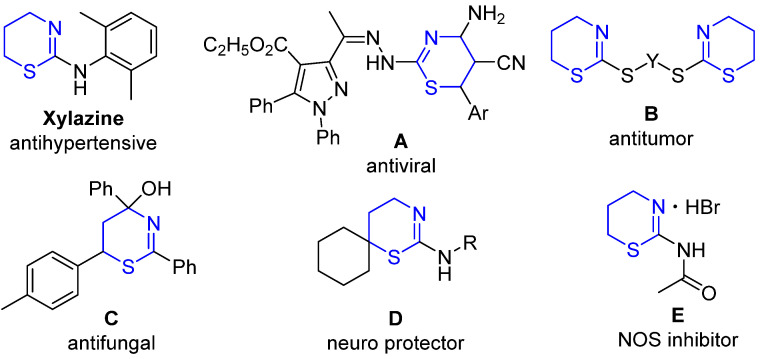
Selected bioactive 5,6-dihydro-4*H*-1,3-thiazine molecules.

Thiazines and their benzo derivatives are regarded as one of the most privileged structures among the multitude of six-membered N,S-heterocyclic scaffolds [1]. In particular, 5,6-dihydro-4*H*-1,3-thiazines have received considerable attention because they are the structural cores of many pharmaceutically active molecules such as antifungal, anticonvulsant, antitubercular, antibacterial, antimicrobial, antitumor, insecticidal, fungicidal, and herbicidal agents, tranquilizers, and so on. As shown in Figure 1, the anti-hypertensive Xylazine is a strong α_2_-adrenergic agonist utilized as a sedative, analgesic, and muscle relaxant in veterinary medicine [2]. The derivative of 2-amino dihydrothiazine (**A**) exhibited antiviral activity against Herpes simplex virus type 1 (HSV-1) [3]. The 2-mercapto dihydrothiazine thioethers (**B**) featured considerable in vitro antitumour IC_50_ values against both A-549 and Bcap-37 cancer cells [4]. The 4-hydroxyl dihydrothiazine molecules (**C**) were found to possess high antifungal activity [5]. The unique spiro dihydrothiazines (**D**) were recognized as neuro protectors involving a blockade of glutamate-induced calcium ion uptake into the brain cortex [6]. The 2-*N*-acetylamino dihydrothiazide (**E**) was found to be approximately twice as effective as 2-aminothiazide in lowering blood pressure [7].

**Figure 2:**
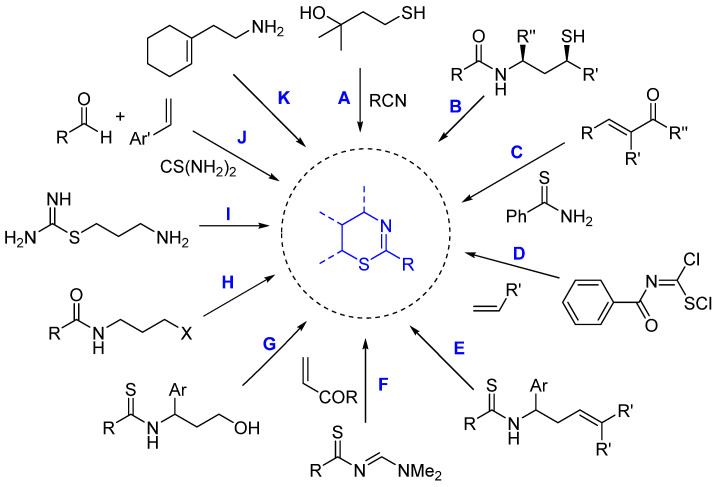
Synthetic routes to 5,6-dihydro-4*H*-1,3-thiazines.

Due to the aforementioned pharmacological properties of six-membered N,S-heterocyclic scaffolds, numerous synthetic approaches toward 5,6-dihydro-4*H*-1,3-thiazines have been developed over the past decades (Figure 2). For instance, reactions starting from 3-hydroxyl thiols [8] (method A), 3-*N*-acylamino thiols [9] (method B), *S*-alkyldithiocarbamates and α,β-unsaturated ketones [5] (method C), *N*-benzoyliminochloromethanesulfenyl chloride and 3,3-dimethyl-2-butene [10] (method D), *N*-homoallyl thioamides [11] (method E), 1-thia-3-aza-buta-1,3-dienes and dienophiles [12] (method F), γ-hydroxyl thioamides [13] (method G), *N*-(3-halogenopropyl)amides [14] (method H), *S*-(aminopropyl)isothiourea [15] (method I), three-component reaction of aromatic aldehydes, thiourea, and vinylbenzenes [16] (method J), and 2-cyclohexenyl ethylamine and isothiocyanates [6] (method K) have been extensive investigated, affording different structures of 5,6-dihydro-4*H*-1,3-thiazines. Moreover, a stereoselective synthesis of 2-susbstituted amino-5,6-dihydro-4*H*-1,3-thiazines via sequential thiourea formation and intramolecular thiol-Michael reaction was also reported [17].

**Figure 3:**
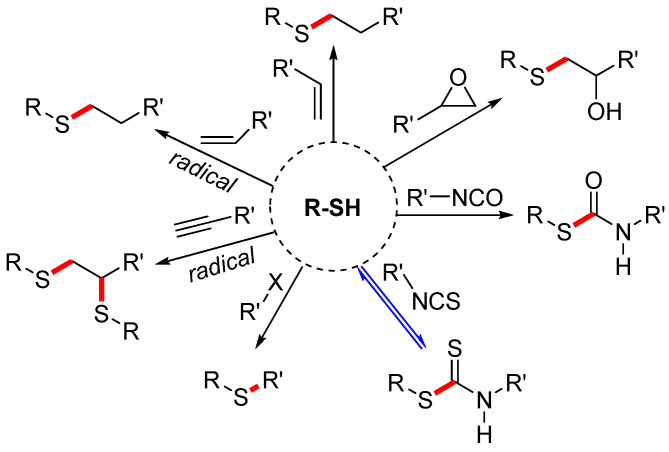
The family of thiol-based click reactions.

Despite the emergence of a multitude of synthetic methods, the pursuit of a more benign and efficient synthetic protocol for dihydrothiazines remains a compelling and valuable endeavor. Meanwhile, a number of rapid, reliable, and selective reactions involving thiols have been identified as click reactions and subjected to extensive investigation. These include the thiol-ene, thiol-yne, thiol-epoxide, thiol-isocyanate, thiol-isothiocyanate, thiol-halogen, and thiol-Michael addition reactions, which can be broadly classified as radical and nucleophilic reactions [18,19,20] (Figure 3). In particular, the reversible thiol-isothiocyanate reaction can produce a dithiocarbamate moiety which is able to tautomerize into the corresponding carbonimidodithioate, thus forming an endogenously reactive SH group [18]. So, we envisioned that, if introducing a leaving group such as halogen into the molecule of isothiocyanate, kinds of intermolecular cyclization should occur, which may result in the desired six-membered N,S-heterocyclic scaffolds.

**Scheme 1:**
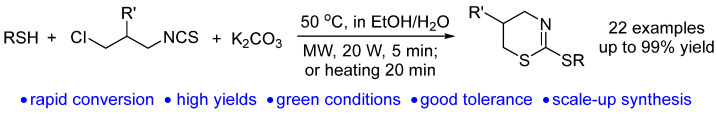
Synthesis of 5,6-dihydro-4*H*-1,3-thiazines via thiol-involved cascade reaction.

Herein we wish to report a simple and benign synthesis of diverse 2-mercapto 5,6-dihydro-4*H*-1,3-thiazines from thiols and 3-chloroisothiocyanates via a thiol-involved cascade reaction under mild green conditions (Scheme 1).

## 2. Results and Discussion

**Scheme 2:**
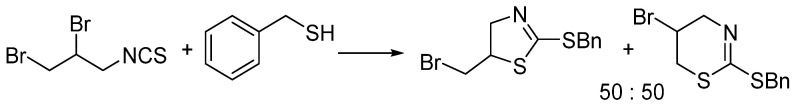
Reaction of 2,3-dibromopropyl isothiocyanate with toluenethiol.

To examine the feasibility of the proposed thiol-involved cascade reaction, we initially used the commercially available 2,3-dibromopropyl isothiocyanate as the starting compound to react with toluenethiol (BnSH). To our delight, two types of N,S-heterocyclic products were then observed. One is the five-membered thiazoline, the other is the expected six-membered 2-mercapto 5,6-dihydro-4*H*-1,3-thiazine, with a 50:50 ratio in the mixture (Scheme 2).

Encouraged by the above findings, we then selected 3-chloropropyl isothiocyanate **1a** with aromatic toluenethiol (BnSH) **2a** for screening. The expected product, dihydrothiazine **3a**, was obtained in only 30% yield after a 60-min solvent-free reaction at room temperature when using 0.6 equiv. K_2_CO_3_ to capture the HCl generated in the mixture (Table 1, Entry 1). Considering environmental benignity, we then tried the reaction focusing on green water and/or alcohol solvents. However, the individual use of H_2_O or EtOH only afforded **3a** in 49% and 31% yields, respectively (Table 1, Entries 2–3), which could be attributed to the low solubility of organic reactants in H_2_O and inorganic K_2_CO_3_ in EtOH. Gratifyingly, the employment of H_2_O and EtOH in a 1:1 ratio resulted in a 78% yield of **3a** (Table 1, Entry 4). When the reaction temperature was increased from 25 °C to 50 °C, the yield reached 96% after a 60-min reaction (Table 1, Entry 5). LC-MS monitoring of the reaction process showed that the reaction time could be shortened to 20 min at 50 °C with a comparative yield of **3a** (Table 1, Entry 6). Inorganic bases such as NaOH, NaHCO_3_, and Na_2_CO_3_, and organic bases such as Et_3_N, 1,8-diazabicyclo[5.4.0]undec-7-ene (DBU) all gave satisfactory but slightly declined yields (Table 1, Entries 7–11). Further screening of other types of commonly used green solvents all led to a varying degree of declined yield of **3a**, including CH_3_COCH_3_ (affording 91% of **3a**) (Table 1, Entry 12), as well as DCM (81% of **3a**), PhMe (37% of **3a**), CH_3_CN (85% of **3a**), and THF (80% of **3a**). Moreover, high to 92% yield of **3a** could also be achieved when using 10% of the amount of solvent (EtOH/H_2_O: 0.1 mL/0.1 mL). To address this, the decreased loading of 0.5 equiv. K_2_CO_3_ gave a slightly declined yield (92%) of **3a** (Table 1, Entry 13), whereas an increased loading of 0.75 equiv. K_2_CO_3_ afforded no improvement for the outcome of **3a** (Table 1, Entry 14). Potassium carbonate was ultimately chosen as the optimal base because K_2_CO_3_ and the only by-product, KCl, are very soluble and can be easily separated simply by washing with H_2_O to afford the high purity of crude products.

Of note, when we employed microwave-assisted means instead of the conventional heating conditions, the reaction was found to be much more rapidly completed in only 5 min. More importantly, the product yield of **3a** was also elevated from 95% to 99%, despite a trace amount of dibenzyl disulfide (BnSSBn) being observed by LC/MS (Table 1, Entry 15). However, a reaction at a higher temperature of 80 °C would result in the relatively greater generation of the by-product BnSSBn, which should be attributed to the disfavored oxidation of thiol by the oxygen in the open-air system (Table 1, Entry 16). In addition, replacement of **1a** by 3-iodopropyl isothiocyanate to react with BnSH showed nearly no efficiency difference (Table 1, Entry 17).

Under the optimal conditions, we investigated the substrate scope of this reaction with two different synthetic methods: the microwave-assisted procedure and the routine heating procedure respectively (Table 2). Regarding the MW-assisted synthesis method, the cascade reaction exhibited excellent tolerances toward different toluenethiols with either electron-withdrawing groups (EWG) or electron-donating groups (EDG), affording dihydrothiazines **3a**–**3g** in nearly quantitative yields (95–99%). Conversely, benzenethiols with EWGs (MeO or *i*Pr) or EDGs (F, Cl, or Br) provided somewhat smaller yields (85–95%) of products **3h**–**3i**. In particular, the reduction of yield (85%) in compound **3i** may be attributed to the steric site-blocking effect. Delightedly, heteroaryl thiols were also well-tolerated to afford products **3m** and **3n** in 93% and 95% yields, respectively. Aliphatic thiol substrates featuring butyl (**3o**), isopropyl (**3p**), cyclohexyl (**3q**), and adamantyl (**3r**) structures all gave nearly quantitative yields of 95–98%.

In comparison, the routine heating method always performed declined reaction outcomes, generally affording relatively lower yields of the dihydrothiazine products, and even no accessibility of some compounds such as product **3k** (Table 2). Because of the relative scarcity of 1-chloro-3-isothiocyanatopropane substrates, we have only found 2-methyl substituted material so far, and obtained compound **3s** in yields of 99% and 95%, respectively, via two different methods.

**Scheme 3:**
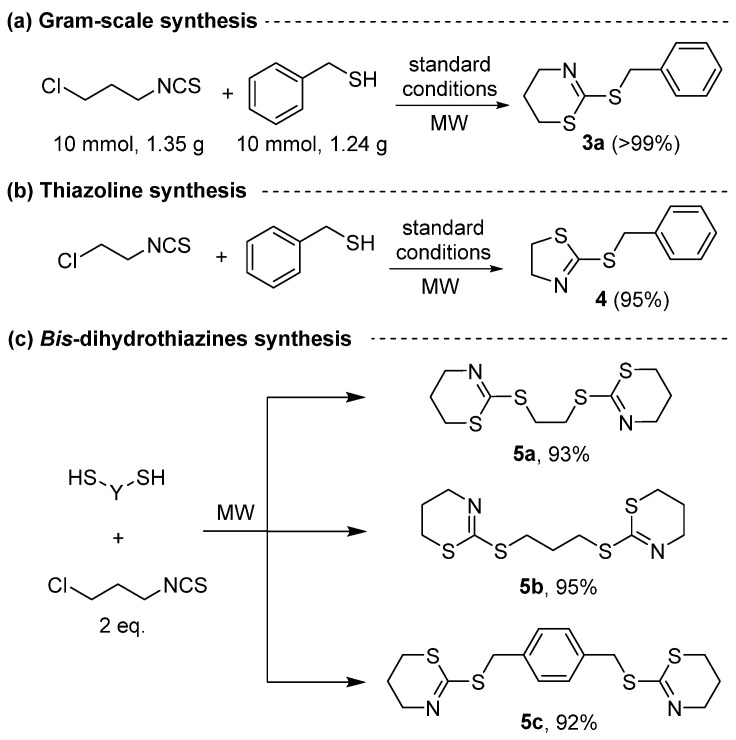
Synthetic application experiments.

To further demonstrate the practicality of the present method for N,S-heterocyclic compounds, some reactions were carried out. Firstly, when 10 mmol of 3-chloropropyl isothiocyanate reacted with toluenethiol (10 mmol) under the standard MW conditions, the expected **3a** was obtained in quantitative yield, which suggests the robust gram-scale synthetic accessibility (Scheme 3a). Inspired by the experiment in Scheme 2, when we employed the 2-chloroethyl isothiocyanate instead of 3-chloropropyl isothiocyanate to react with toluenethiol under the standard MW conditions, the five-membered N,S-heterocyclic thiazoline **4** was obtained in high to 95% yield, which reveals that our procedure should also be applicable for the construction of other types of N,S-heterocyclic scaffolds (Scheme 3b). Importantly, the easy synthesis of *bis*-dihydrothiazines, which were recognized as displaying antitumor activity [4], was also achieved efficiently using our standard microwave-assisted synthetic method, hence affording three molecules of **5a**–**5c** in 92–95% yields (Scheme 3c). The synthetic application experiments collectively demonstrate the considerable potential of this new reaction system for industrial preparations.

**Figure 4:**
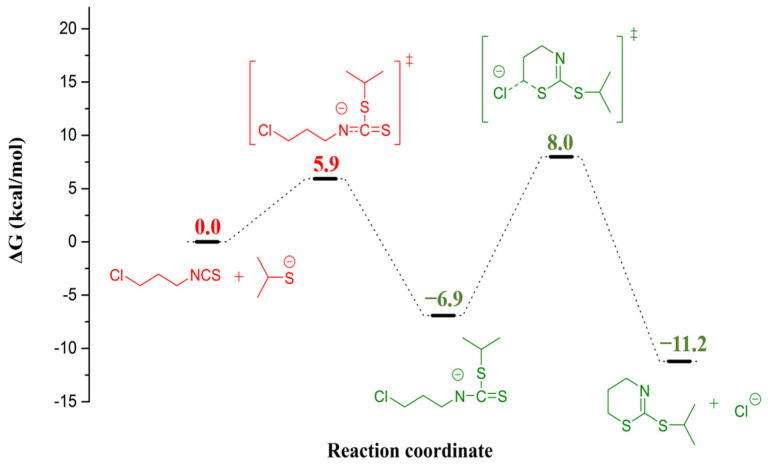
DFT calculation of the reaction process.

In order to gain further insight into the reaction mechanism, an ESI-MS monitoring experiment was conducted, yet failed to capture the supposed intermediate adducts, even at a lower temperature of 0 °C, which suggests that the sequence thiol click reactions should occur at a very high speed. To verify the rationality of the reaction mechanism, a density functional theory (DFT) calculation about the reaction process of product **3p** was then performed, as shown in Figure 4 (computational details see Supplementary Materials).

The barrier of the first elementary reaction, which involves the formation of the first S–C bond through intermolecular nucleophilic addition, was 5.9 kcal/mol. Then, the second elementary reaction for the second S–C bond formation was an intramolecular S_N_2 nucleophilic substitution, resulting in the construction of the six-membered ring, with a corresponding reaction barrier of 14.9 kcal/mol. From the barrier diagram of the cascade S–C coupling reaction, it could be observed that the overall energy of the reaction exhibited a noticeable downward trend (∆G = −11.2 kcal/mol).

**Figure 5:**
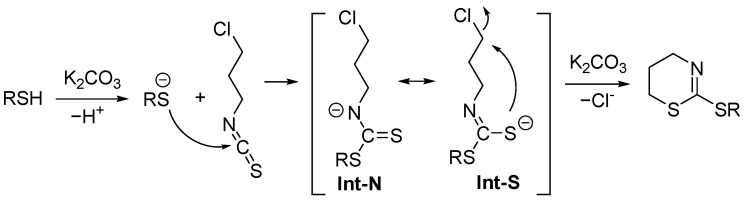
Proposed reaction mechanism.

On the basis of the experimental results and related literature [21], our metal- and ligand-free synthesis of 5,6-dihydro-4*H*-1,3-thiazines should proceed through an addition–substitution pathway, as depicted in Figure 5. The intermolecular nucleophilic addition of thiol anion to isothiocyanate leads to the formation of dithiocarbamate (**Int-N**), which tautomerizes into the corresponding carbonimidodithioate (**Int-S**). A rapid intramolecular nucleophilic substitution of the γ-chlorine atom by the newly-generated thiol group in **Int-S** affords the desired double S–C coupling product of 2-mercapto dihydrothiazide and one equivalent HCl to be neutralized by the base.

## 3. Experimental

All the commercially available reagents were used without further purification unless otherwise stated. Reactions were monitored by LC/MS or thin layer chromatography (TLC). NMR spectra were recorded on Bruker 300 instruments (BRUKER, Romanshorn, Switzerland) and calibrated using residual undeuterated solvents. Flash column chromatography was performed using Qingdao Haiyang (Haiyang, China) silica (silica gel for thin-layer chromatography, HG/T2354-2010 [22]). High-resolution mass spectra (HRMS) were obtained on a Thermo Scientific Q Exactive HPLC and mass spectrometry (Thermo Fisher Scientific, Waltham, MA, USA).

**General procedure for synthesis of 3.**

MW Method: To a stirred solution of 3-chloropropyl isothiocyanate (0.2 mmol) and thiol (0.2 mmol) in EtOH/H_2_O (1 mL/1 mL) was added K_2_CO_3_ (0.6 eq.) under air, and then reacted under MW irradiation (20 W) for about 5 min at 50 °C. Dichloromethane (5 mL) was added, and the two layers were separated, and the aqueous layer was extracted twice with dichloromethane (2 × 5 mL). The combined organic layers were washed with saturated aqueous brine (2 × 10 mL) and dried over anhydrous Na_2_SO_4_. The crude products were purified by column chromatography (silica gel, *n*-hexane: ethyl acetate = 10:1) to afford the desired products.

Heat Method: To a stirred solution of 3-chloropropyl isothiocyanate (0.2 mmol) and thiol (0.2 mmol) in EtOH/H_2_O (1 mL/1 mL) was added K_2_CO_3_ (0.6 eq.) under air, which was then reacted for about 20 min at 50 °C. Dichloromethane (5 mL) was added, and the aqueous layer was extracted twice with dichloromethane (2 × 5 mL). The combined organic layers were washed with saturated aqueous brine (2 × 10 mL) and dried over anhydrous Na_2_SO_4_. The crude products were purified by column chromatography (silica gel, *n*-hexane: ethyl acetate = 10:1) to afford the desired products.

*2-(benzylthio)-5,6-dihydro-4H-1,3-thiazine* (**3a**), Oily liquid, yield 99%, 46 mg; ^1^H NMR (300 MHz, Chloroform-*d*) δ 7.27–7.13 (m, 5H), 4.16 (s, 2H), 3.76–3.64 (m, 2H), 3.04–2.92 (m, 2H), 1.84 (dd, *J* = 11.8, 5.6 Hz, 2H). ^13^C NMR (75 MHz, Chloroform-*d*) δ 155.0, 137.4, 129.1, 128.5, 127.2, 48.5, 34.5, 27.7, 20.6. HRMS (ESI-TOF, *m*/*z*): calcd for C_11_H_14_NS_2_ [M + H]^+^ 224.0562, found 224.0559.

*2-((4-methylbenzyl)thio)-5,6-dihydro-4H-1,3-thiazine* (**3b**), Oily liquid, yield 97%, 46 mg; ^1^H NMR (300 MHz, Chloroform-*d*) δ 7.23–7.06 (m, 4H), 4.19 (s, 2H), 3.80–3.72 (m, 2H), 3.10–3.00 (m, 2H), 2.31 (s, 3H), 1.90 (dt, *J* = 12.0, 5.9 Hz, 2H). ^13^C NMR (75 MHz, Chloroform-*d*) δ 155.1, 136.8, 134.2, 129.2, 129.0, 48.5, 34.4, 27.7, 21.2, 20.6. HRMS (ESI-TOF, *m*/*z*): calcd for C_12_H_16_NS_2_ [M + H]^+^ 238.0719, found 238.0717.

*2-((2-methylbenzyl)thio)-5,6-dihydro-4H-1,3-thiazine* (**3c**), Oily liquid, yield 95%, 45 mg; ^1^H NMR (300 MHz, Chloroform-*d*) δ 7.28 (d, *J* = 5.6 Hz, 1H), 7.14 (d, *J* = 1.6 Hz, 3H), 4.24 (s, 2H), 3.81–3.73 (m, 2H), 3.09–3.01 (m, 2H), 2.36 (s, 3H), 1.91 (p, *J* = 5.9 Hz, 2H). ^13^C NMR (75 MHz, Chloroform-*d*) δ 155.4, 137.1, 134.8, 130.4, 130.2, 127.6, 126.1, 48.5, 32.8, 27.8, 20.6, 19.3. HRMS (ESI-TOF, *m*/*z*): calcd for C_12_H_16_NS_2_ [M + H]^+^ 238.0719, found 238.0715.

*2-((4-methoxybenzyl)thio)-5,6-dihydro-4H-1,3-thiazine* (**3d**), Oily liquid, yield 98%, 50 mg; ^1^H NMR (300 MHz, Chloroform-*d*) δ 7.24 (d, *J* = 8.6 Hz, 2H), 6.82 (d, *J* = 8.7 Hz, 2H), 4.18 (s, 2H), 3.77 (d, *J* = 4.0 Hz, 5H), 3.11–2.98 (m, 2H), 1.90 (dt, *J* = 12.2, 5.9 Hz, 2H). ^13^C NMR (75 MHz, Chloroform-*d*) δ 158.7, 155.1, 130.2, 129.2, 113.9, 55.3, 48.5, 34.1, 27.7, 20.6. HRMS (ESI-TOF, *m*/*z*): calcd for C_12_H_16_NOS_2_ [M + H]^+^ 254.0668, found 254.0665.

*2-((4-fluorobenzyl)thio)-5,6-dihydro-4H-1,3-thiazine* (**3e**), Oily liquid, yield 98%, 47 mg; ^1^H NMR (300 MHz, Chloroform-*d*) δ 7.21 (dd, *J* = 8.5, 5.5 Hz, 2H), 6.89 (t, *J* = 8.7 Hz, 2H), 4.11 (s, 2H), 3.74–3.64 (m, 2H), 3.02–2.94 (m, 2H), 1.83 (dt, *J* = 12.2, 5.9 Hz, 2H). ^13^C NMR (75 MHz, Chloroform-*d*) δ 161.9 (d, *J* = 244.5 Hz), 154.7, 133.3 (d, *J* = 3 Hz), 130.7 (d, *J* = 7.5 Hz), 115.3 (d, *J* = 21 Hz), 48.5, 33.7, 27.7, 20.5. ^19^F NMR (282 MHz, Chloroform-*d*) δ −115.43. HRMS (ESI-TOF, *m*/*z*): calcd for C_11_H_13_FNS_2_ [M + H]^+^ 242.0468, found 242.0466.

*2-((4-chlorobenzyl)thio)-5,6-dihydro-4H-1,3-thiazine* (**3f**), Oily liquid, yield 97%, 50 mg; ^1^H NMR (300 MHz, Chloroform-*d*) δ 7.18 (s, 4H), 4.11 (s, 2H), 3.73–3.64 (m, 2H), 3.02–2.93 (m, 2H), 1.83 (dt, *J* = 12.1, 5.9 Hz, 2H). ^13^C NMR (75 MHz, Chloroform-*d*) δ 154.7, 136.2, 132.9, 130.4, 128.6, 48.4, 33.7, 27.7, 20.5. HRMS (ESI-TOF, *m*/*z*): calcd for C_11_H_13_ClNS_2_ [M + H]^+^ 258.0172, found 258.0170.

*2-((4-bromobenzyl)thio)-5,6-dihydro-4H-1,3-thiazine* (**3g**), Oily liquid, yield 98%, 59 mg; ^1^H NMR (300 MHz, Chloroform-*d*) δ 7.40 (d, *J* = 8.3 Hz, 2H), 7.19 (d, *J* = 8.4 Hz, 2H), 4.16 (s, 2H), 3.80–3.70 (m, 2H), 3.10–3.00 (m, 2H), 1.97–1.83 (m, 2H). ^13^C NMR (75 MHz, Chloroform-*d*) δ 154.7, 136.8, 131.5, 130.8, 121.0, 48.4, 33.7, 27.7, 20.5. HRMS (ESI-TOF, *m*/*z*): calcd for C_11_H_13_BrNS_2_ [M + H]^+^ 301.9667, found 301.9666.

*2-((4-methoxyphenyl)thio)-5,6-dihydro-4H-1,3-thiazine* (**3h**), Oily liquid, yield 95%, 45 mg; ^1^H NMR (300 MHz, Chloroform-*d*) δ 7.52 (d, *J* = 8.7 Hz, 2H), 6.90 (d, *J* = 8.8 Hz, 2H), 3.82 (s, 3H), 3.76–3.69 (m, 2H), 3.03–2.95 (m, 2H), 1.84 (p, *J* = 5.7 Hz, 2H). ^13^C NMR (75 MHz, Chloroform-*d*) δ 161.0, 158.2, 138.1, 119.0, 114.5, 55.4, 48.8, 28.1, 19.7. HRMS (ESI-TOF, *m*/*z*): calcd for C_11_H_14_NOS_2_ [M + H]^+^ 240.0511, found 240.0508.

*2-((2-isopropylphenyl)thio)-5,6-dihydro-4H-1,3-thiazine* (**3i**), Oily liquid, yield 85%, 43 mg; ^1^H NMR (300 MHz, Chloroform-*d*) δ 7.60 (d, *J* = 7.7 Hz, 1H), 7.45–7.32 (m, 2H), 7.21–7.14 (m, 1H), 3.76–3.57 (m, 3H), 3.03–2.93 (m, 2H), 1.84 (dt, *J* = 12.1, 5.8 Hz, 2H), 1.23 (d, *J* = 6.9 Hz, 6H). ^13^C NMR (75 MHz, Chloroform-*d*) δ 157.5, 153.4, 137.8, 130.7, 126.9, 126.3, 126.1, 48.9, 31.1, 28.1, 23.7, 19.7. HRMS (ESI-TOF, *m*/*z*): calcd for C_13_H_18_NS_2_ [M + H]^+^ 252.0875, found 252.0872.

*2-((4-fluorophenyl)thio)-5,6-dihydro-4H-1,3-thiazine* (**3j**), Oily liquid, yield 90%, 41 mg; ^1^H NMR (300 MHz, Chloroform-*d*) δ 7.54–7.44 (m, 2H), 6.99 (t, *J* = 8.7 Hz, 2H), 3.69–3.60 (m, 2H), 3.01–2.88 (m, 2H), 1.82–1.75 (m, 2H). ^13^C NMR (75 MHz, Chloroform-*d*) δ 163.7 (d, *J* = 249 Hz), 156.9, 138.1 (d, *J* = 8.25 Hz), 124.0 (d, *J* = 3 Hz), 116.2 (d, *J* = 21.75 Hz), 48.9, 28.1, 19.7. ^19^F NMR (282 MHz, Chloroform-*d*) δ -110.80. HRMS (ESI-TOF, *m*/*z*): calcd for C_10_H_11_FNS_2_ [M + H]^+^ 228.0311, found 228.0309.

*2-((4-chlorophenyl)thio)-5,6-dihydro-4H-1,3-thiazine* (**3k**), Oily liquid, yield 91%, 44 mg; ^1^H NMR (300 MHz, Chloroform-*d*) δ 7.43 (d, *J* = 8.5 Hz, 2H), 7.26 (d, *J* = 8.5 Hz, 2H), 3.69–3.61 (m, 2H), 2.99–2.91 (m, 2H), 1.82–1.75 (m, 2H). ^13^C NMR (75 MHz, Chloroform-*d*) δ 156.2, 136.9, 135.8, 129.2, 127.5, 49.0, 28.2, 19.8. HRMS (ESI-TOF, *m*/*z*): calcd for C_10_H_11_ClNS_2_ [M + H]^+^ 244.0016, found 244.0014.

*2-((4-bromophenyl)thio)-5,6-dihydro-4H-1,3-thiazine* (**3l**), Oily liquid, yield 91%, 52 mg; ^1^H NMR (300 MHz, Chloroform-*d*) δ 7.52–7.39 (m, 4H), 3.73 (t, *J* = 5.6 Hz, 2H), 3.08–2.98 (m, 2H), 1.87 (p, *J* = 5.7 Hz, 2H). ^13^C NMR (75 MHz, Chloroform-*d*) δ 156.1, 137.0, 132.2, 128.1, 124.1, 49.0, 28.2, 19.8. HRMS (ESI-TOF, *m*/*z*): calcd for C_10_H_11_BrNS_2_ [M + H]^+^ 287.9511, found 287.9509.

*2-((furan-2-ylmethyl)thio)-5,6-dihydro-4H-1,3-thiazine* (**3m**), Oily liquid, yield 93%, 40 mg; ^1^H NMR (300 MHz, Chloroform-*d*) δ 7.26 (s, 1H), 6.24–6.19 (m, 1H), 6.13 (d, *J* = 2.9 Hz, 1H), 4.20 (s, 2H), 3.75–3.64 (m, 2H), 3.05–2.94 (m, 2H), 1.84 (dt, *J* = 12.1, 5.9 Hz, 2H). ^13^C NMR (75 MHz, Chloroform-*d*) δ 154.2, 150.8, 142.1, 110.6, 108.0, 48.5, 27.7, 26.6, 20.6. HRMS (ESI-TOF, *m*/*z*): calcd for C_9_H_12_NOS_2_ [M + H]^+^ 214.0355, found 214.0352.

*2-(thiophen-2-ylthio)-5,6-dihydro-4H-1,3-thiazine* (**3n**), Oily liquid, yield 95%, 41 mg; ^1^H NMR (300 MHz, Chloroform-*d*) δ 7.57 (d, *J* = 5.3 Hz, 1H), 7.33 (d, *J* = 3.6 Hz, 1H), 7.14–7.02 (m, 1H), 3.76 (t, *J* = 5.6 Hz, 2H), 3.07–2.96 (m, 2H), 1.87 (p, *J* = 5.8 Hz, 2H). ^13^C NMR (75 MHz, Chloroform-*d*) δ 158.1, 138.6, 133.1, 127.8, 126.0, 48.9, 27.9, 19.5. HRMS (ESI-TOF, *m*/*z*): calcd for C_8_H_11_NS_3_ [M + H]^+^ 215.9970, found 215.9969.

*2-(butylthio)-5,6-dihydro-4H-1,3-thiazine* (**3o**), Oily liquid, yield 98%, 37 mg; ^1^H NMR (300 MHz, Chloroform-*d*) δ 3.72–3.61 (m, 2H), 3.04–2.86 (m, 4H), 1.83 (dt, *J* = 12.2, 5.9 Hz, 2H), 1.58–1.46 (m, 2H), 1.33 (dq, *J* = 14.0, 7.2 Hz, 2H), 0.84 (t, *J* = 7.3 Hz, 3H). ^13^C NMR (75 MHz, Chloroform-*d*) δ 155.4, 48.5, 31.3, 30.0, 27.7, 22.0, 20.5, 13.7. HRMS (ESI-TOF, *m*/*z*): calcd for C_8_H_16_NS_2_ [M + H]^+^ 190.0719, found 190.0717.

*2-(isopropylthio)-5,6-dihydro-4H-1,3-thiazine* (**3p**), Oily liquid, yield 95%, 33 mg; ^1^H NMR (300 MHz, Chloroform-*d*) δ 3.81–3.63 (m, 3H), 3.03–2.94 (m, 2H), 1.83 (dt, *J* = 11.1, 5.9 Hz, 2H), 1.25 (d, *J* = 6.9 Hz, 6H). 13C NMR (75 MHz, Chloroform-*d*) δ 155.2, 48.5, 35.8, 27.9, 23.0, 20.5. HRMS (ESI-TOF, *m*/*z*): calcd for C_7_H_14_NS_2_ [M + H]^+^ 176.0562, found 176.0561.

*2-(cyclohexylthio)-5,6-dihydro-4H-1,3-thiazine* (**3q**), Oily liquid, yield 96%, 41 mg; ^1^H NMR (300 MHz, Chloroform-*d*) δ 3.70–3.54 (m, 3H), 3.03–2.90 (m, 2H), 1.94–1.48 (m, 7H), 1.39–1.15 (m, 5H). ^13^C NMR (75 MHz, Chloroform-*d*) δ 155.0, 48.6, 43.4, 33.1, 27.9, 25.9, 25.7, 20.5. HRMS (ESI-TOF, *m*/*z*): calcd for C_10_H_18_NS_2_ [M + H]^+^ 216.0875, found 216.0873.

*2-(((1r,3R,5S)-adamantan-1-yl)thio)-5,6-dihydro-4H-1,3-thiazine* (**3r**), White solid, yield 96%, 51 mg; ^1^H NMR (300 MHz, Chloroform-*d*) δ 3.74–3.67 (m, 2H), 3.00–2.91 (m, 2H), 2.04 (d, *J* = 2.7 Hz, 9H), 1.83–1.76 (m, 2H), 1.62 (s, 6H). ^13^C NMR (75 MHz, Chloroform-*d*) δ 153.4, 52.1, 48.9, 43.3, 36.3, 30.1, 28.9, 19.8. HRMS (ESI-TOF, *m*/*z*): calcd for C_14_H_22_NS_2_ [M + H]^+^ 268.1188, found 268.1185.

*2-(benzylthio)-5-methyl-5,6-dihydro-4H-1,3-thiazine* (**3s**), Oily liquid, yield 99%, 47 mg; ^1^H NMR (300 MHz, Chloroform-*d*) δ 7.27–7.13 (m, 5H), 4.16 (s, 2H), 3.83 (m, 1H), 3.18 (dd, *J* = 16.0, 9.6 Hz, 1H), 2.84 (m, 1H), 2.79–2.65 (m, 1H), 1.88 (m, 1H), 0.97 (d, *J* = 6.7 Hz, 3H). ^13^C NMR (75 MHz, Chloroform-*d*) δ 154.5, 137.4, 129.1, 128.5, 127.2, 55.6, 34.6, 34.0, 25.5, 18.8. HRMS (ESI-TOF, *m*/*z*): calcd for C_12_H_16_NS_2_ [M + H]^+^ 238.0719, found 238.0715.

*2-(benzylthio)-4,5-dihydrothiazole* (**4**), Oily liquid, yield 95%, 40 mg; ^1^H NMR (300 MHz, Chloroform-*d*) δ 7.32–7.14 (m, 5H), 4.28 (s, 2H), 4.15 (t, *J* = 8.0 Hz, 2H), 3.32 (t, *J* = 8.0 Hz, 2H). ^13^C NMR (75 MHz, Chloroform-*d*) δ 165.4, 136.6, 129.1, 128.6, 127.5, 64.3, 37.0, 35.7. HRMS (ESI-TOF, *m*/*z*): calcd for C_10_H_12_NS_2_ [M + H]^+^ 210.0406, found 210.0403.

*1,2-bis((5,6-dihydro-4H-1,3-thiazin-2-yl)thio)ethane* (**5a**), Oily liquid, yield 93%, 54 mg; ^1^H NMR (300 MHz, Chloroform-*d*) δ 3.71–3.61 (m, 2H), 3.51 (dd, *J* = 7.3, 5.2 Hz, 2H), 3.38–3.23 (m, 4H), 3.04–2.94 (m, 4H), 1.98–1.79 (m, 4H). ^13^C NMR (75 MHz, Chloroform-*d*) δ 168.2, 154.9, 57.9, 48.5, 37.8, 34.7, 30.2, 28.2, 27.8, 20.5. HRMS (ESI-TOF, *m*/*z*): calcd for C_10_H_17_N_2_S_4_ [M + H]^+^ 293.0269, found 293.0265.

*1,3-bis((5,6-dihydro-4H-1,3-thiazin-2-yl)thio)propane* (**5b**), Oily liquid, yield 95%, 58 mg; ^1^H NMR (300 MHz, Chloroform-*d*) δ 3.72–3.59 (m, 4H), 2.98 (td, *J* = 7.2, 6.5, 2.7 Hz, 8H), 1.91–1.78 (m, 6H). ^13^C NMR (75 MHz, Chloroform-*d*) δ 154.7, 48.4, 29.3, 29.1, 27.8, 20.5. HRMS (ESI-TOF, *m*/*z*): calcd for C_11_H_19_N_2_S_4_ [M + H]^+^ 307.0426 found 307.0421.

*1,4-bis(((5,6-dihydro-4H-1,3-thiazin-2-yl)thio)methyl)benzene* (**5c**), Oily liquid, yield 92%, 68 mg; ^1^H NMR (300 MHz, Chloroform-*d*) δ 7.17 (s, 4H), 4.12 (s, 4H), 3.73–3.65 (m, 4H), 3.02–2.93 (m, 4H), 1.83 (dt, *J* = 12.1, 5.9 Hz, 4H). ^13^C NMR (75 MHz, Chloroform-*d*) δ 155.0, 136.3, 129.2, 48.5, 34.2, 27.7, 20.6. HRMS (ESI-TOF, *m*/*z*): calcd for C_16_H_21_N_2_S_4_ [M + H]^+^ 369.0582, found 369.0577.

## 4. Conclusions

In summary, we have reported a new synthetic route to the six-membered N,S-heterocyclic molecules of 2-mercapto 5,6-dihydro-4*H*-1,3-thiazines based on a double C–S linking methodology. In total, twenty-two structurally diverse dihydrothiazines, including three bioactive *bis*-dihydrothiazine compounds, were efficiently afforded in high yields from the readily available 3-chloropropyl isothiocyanates and various thiols in the green EtOH/H_2_O (1:1) solvents using K_2_CO_3_ as the base. The two protocols, employing microwave or heating means, respectively, both provided mild conditions, rapid completion, high reliability, and good scopes. DFT calculation supported the supposed cascade intermolecular thiol-isothiocyanate addition and intramolecular thiol-chloride substitution mechanism.

## Figures and Tables

**Table 1:** Screening of the thiol-involved cascade reaction *^a^*.

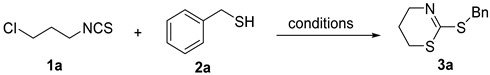					
Entry	Base (eq.)	Solvent	Temp. (°C)	Time (min)	3a (%) *^b^*
1	K_2_CO_3_ (0.6)	neat	25	60	30
2	K_2_CO_3_ (0.6)	H_2_O	25	60	49
3	K_2_CO_3_ (0.6)	EtOH	25	60	31
4	K_2_CO_3_ (0.6)	EtOH/H_2_O	25	60	78
5	K_2_CO_3_ (0.6)	EtOH/H_2_O	50	60	96
**6**	**K_2_CO_3_ (0.6)**	**EtOH/H_2_O**	**50**	**20**	**95**
7	NaOH (1.2)	EtOH/H_2_O	50	20	94
8	Na_2_CO_3_ (0.6)	EtOH/H_2_O	50	20	94
9	NaHCO_3_ (1.2)	EtOH/H_2_O	50	20	92
10	Et_3_N (1.2)	EtOH/H_2_O	50	20	94
11	DBU (1.2)	EtOH/H_2_O	50	20	94
12	K_2_CO_3_ (0.6)	Acetone	50	20	91
13	K_2_CO_3_ (0.5)	EtOH/H_2_O	50	20	92
14	K_2_CO_3_ (0.75)	EtOH/H_2_O	50	20	95
**15 *^c^***	**K_2_CO_3_ (0.6)**	**EtOH/H_2_O**	**50**	**5**	**99**
16 ***^c^***	K_2_CO_3_ (0.6)	EtOH/H_2_O	80	5	95
17 *^d^*	K_2_CO_3_ (0.6)	EtOH/H_2_O	50	5	98

*^a^* Reaction conditions: **1a** (0.2 mmol), **2a** (0.2 mmol), the loading of base based on **1a**. EtOH (1 mL), H_2_O (1 mL); *^b^* isolated yields; *^c^* MW, 20 W; *^d^* using 3-iodopropyl isothiocyanate, MW, 20 W.

**Table 2:** Scope of 5,6-dihydro-4*H*-1,3-thiazines **3** *^a,b^*.

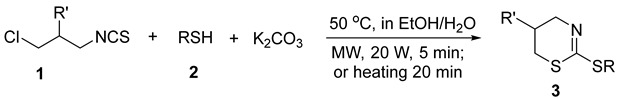		
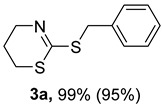	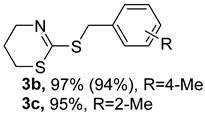	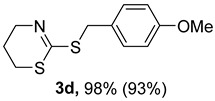
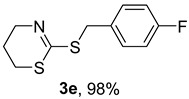	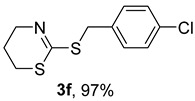	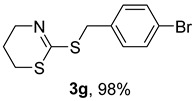
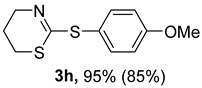	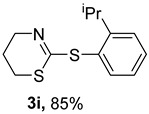	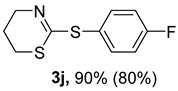
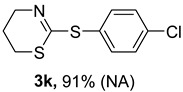	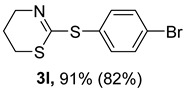	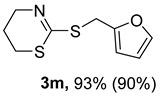
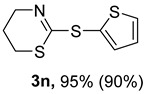	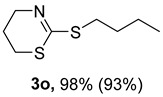	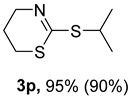
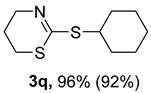	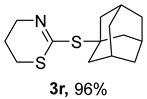	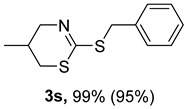

*^a^* Reaction conditions: **1** (0.2 mmol), **2** (0.2 mmol), K_2_CO_3_ (0.12 mmol), EtOH (1 mL), H_2_O (1 mL), 50 °C, 20 min; *^b^* isolated yields (by MW), bracketed are from heating method.

## Data Availability

The original contributions presented in the study are included in the article/Supplementary Materials. Further inquiries can be directed to the corresponding authors.

## Supplementary Materials

The following supporting information can be downloaded at: https://www.mdpi.com/article/10.3390/molecules29225255/s1, General information, General procedure for synthesis of compounds **3**, DFT Calculation, NMR spectra of products **3**–**5**, HRMS of products **3**–**5**.
