# Supplementary material for: Green Synthesis of 2-Mercapto 5,6-Dihydro-4H-1,3-Thiazines via Sequential C–S Couplings
Source: Molecules. 2024 Nov 6;29(22):5255. doi: 10.3390/molecules29225255 (PMC11596141; doi:10.3390/molecules29225255)
Supplement: Supplementary file 1 [file molecules-29-05255-s001.zip › molecules-3284953-supplementary.pdf]

Supporting Information

Green Synthesis of 2-Mercapto 5,6-Dihydro-4*H*-1,3-Thiazines via Sequential C–S Couplings

Wenjie Liu [1,2], Shuo Wang [1], Li Pan [1], Xiaojing Bi [1,*] and Enxue Shi [1,*]

[1] State Key Laboratory of NBC Protection for Civilian, Beijing 102205, China; l2507338854@163.com (W.L.); ws20140506@163.com (S.W.); bk6180b@163.com (L.P.)

[2] School of Chemistry and Environmental Engineering, Wuhan Institute of Technology, Wuhan 430205, China

* Correspondence: xiaojingbimail@yeah.net (X.B.); exshi@sina.com (E.S.)

Contents

1. General information 1
2. General procedure for synthesis of compounds **3** 2
3. DFT Calculation.. 3
4. NMR spectra of products **3-5** 8
5. HRMS of products **3-5** 32

1. General information

Reagents were purchased at the highest commercial quality and used without further purification, unless otherwise stated. All the reactions were performed in oven-dried glass vial under microwave irradiation (CEM Discover 2.0 Microwave Synthesis Reactor). Reactions were monitored by LC/MS or thin layer chromatography (TLC). NMR spectra were recorded on Bruker 300 instruments and calibrated using residual undeuterated solvents. The following abbreviations were used to explain multiplicities: s = singlet, d = doublet, t = triplet, q = quartet, m = multiplet, br = broad. Flash column chromatography was performed using Qingdao Haiyang silica (silica gel for thin-layer chromatography, HG/T2354-2010). High-resolution mass spectra (HRMS) were obtained on a Thermo Scientific Q Exactive HPLC and mass spectrometry.

2. General procedure for synthesis of compounds 3

To a stirred solution of 3-chloropropyl isothiocyanate (**3a**-**3r**, **3v**:0.2 mmol; **3s**-**3u**:0.4 mmol) and aromatic or aliphatic thiol (0.2 mmol) in EtOH/H_2O (1 mL/1 mL) was added K_2CO_3 (0.6 eq.) and stirred at 50 °C for 5 minutes under microwave (MW) conditions. Dichloromethane (5 mL) was added, and the two layers were separated. The aqueous layer was then extracted twice with dichloromethane (2 × 5 mL). The combined organic layers were washed with saturated aqueous brine (2 × 10 mL), and dried over anhydrous Na_2SO_4. The crude products were purified by column chromatography (silica gel, *n*-hexane: ethyl acetate =10:1) to afford the desired pure product **3a-3u**.

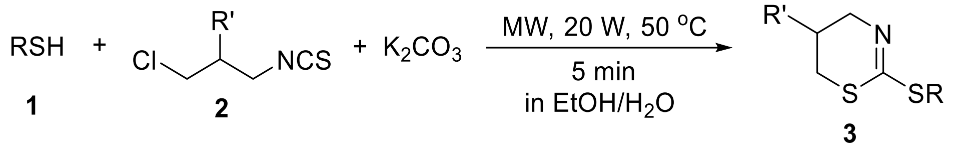

3. DFT Calculation

All calculations were carried out with the density functional theory (DFT) at the M06-2X level of theory using Gaussian 16 series of programs. The def2-SV basis set was used for all the atoms. The gas-phase geometries of all intermediates and transition states were fully optimized without any symmetry restriction, following harmonic frequency calculations to ensure that the local minima had zero imaginary frequencies and the transition state one and to derive the thermal corrections for Gibbs free energies. The transition states had been verified by intrinsic reaction coordinate (IRC) calculation and imaginary vibration modes, which linked reactants and products. Double hybrid functional (B2PLYP method), which could give more accurate energetic information, was used to calculate single point energies with def2-TZVP basis set. Gibbs free energies of all stationary points were obtained by the thermal correction to Gibbs free energy in gas phase and single point energy in gas phase.

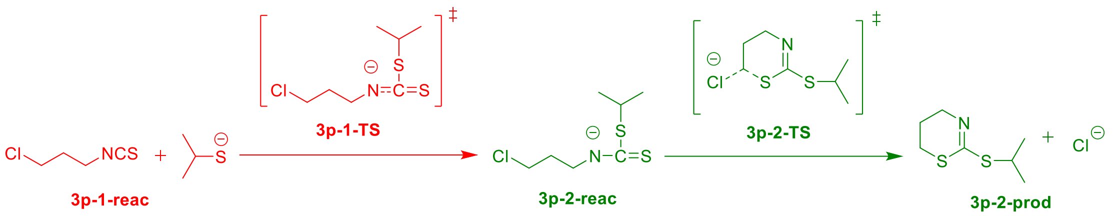

2D Structures of all stationary points

Gibbs free energies of all stationary points

Geometry	$E_{(gas\text{-}B2PLYP)}$[a] (Hartree)	$G_{(corr\text{-}M062X)}$ [b] (Hartree)	IF[c]	ΔG[d] (kcal/mol)
**3p-1-reac**	-1585.4166555085	0.143963	/	0.0
**3p-1-TS**	-1585.4119822910	0.148748	-104.49	5.9
**3p-2-reac**	-1585.4360071091	0.152298	/	-6.9
**3p-2-TS**	-1585.4114372963	0.151499	-418.12	8.0
**3p-2-prod**	-1585.4427744069	0.152203	/	-11.2

[a] The electronic energy calculated by B2PLYP in gas phase. [b] The thermal correction to Gibbs free energy calculated by M062X in gas phase. [c] The M062X calculated imaginary frequencies for the transition states. [d] ΔG = [$G_{(corr\text{-}M062X)}$ + $E_{(gas\text{-}B2PLYP)}$] (Sum of electronic and thermal free energies for transition state) - [$G_{(corr\text{-}M062X)}$ + $E_{(gas\text{-}B2PLYP)}$] (Sum of electronic and thermal free energies for reactant) or ΔG = [$G_{(corr\text{-}M062X)}$ + $E_{(gas\text{-}B2PLYP)}$] (Sum of electronic and thermal free

energies for product) - [$G_{(corr-M062X)}$ + $E_{(gas-B2PLYP)}$] (Sum of electronic and thermal free energies for reactant)

Optimized Geometries for the Compounds and Transition State:

3p-1-reac

C	0.34120400	2.40383200	0.36749300
S	0.97550800	-2.38865300	0.45500700
S	0.93425600	3.65445600	-0.44626300
C	2.19340300	-1.13723700	-0.09548200
H	2.21525800	-0.29844700	0.63211500
C	1.83566300	-0.54284000	-1.45848700
H	0.84731900	-0.05268600	-1.42351700
H	2.57889500	0.20810000	-1.79656200
H	1.77391100	-1.35434200	-2.20584000
C	3.60058200	-1.73509900	-0.13483500
H	4.36549100	-0.99092200	-0.43995500
H	3.86647900	-2.13811900	0.85627200
H	3.61953200	-2.57804600	-0.84898400
N	-0.09974000	1.50186800	0.97443400
C	-0.59407600	0.35178500	1.65237900
H	0.09034300	-0.52095700	1.47151800
H	-0.62575600	0.57954000	2.73119200
C	-1.98328900	-0.05419800	1.15694400
H	-2.72256600	0.74648500	1.33606100
H	-2.28671000	-0.93999100	1.73951100
C	-1.95638200	-0.42872800	-0.31411100
H	-1.66872100	0.42407500	-0.94608700
H	-1.25527600	-1.26788900	-0.47617700
Cl	-3.60463900	-0.92516500	-0.86081700

3p-1-TS

C	0.34359500	-1.89289300	0.09985100
S	1.04340600	0.77487300	-1.02350600
S	1.53724400	-2.21399600	1.11724200
C	2.56330900	0.88128700	-0.02900700
H	2.96711800	-0.14092200	0.09957200
C	2.27764900	1.43826400	1.36513400
H	1.55272400	0.79034400	1.88477700
H	3.19835400	1.50666100	1.98147800
H	1.83218600	2.44601800	1.27709200
C	3.62184100	1.72448800	-0.74146400
H	4.56831700	1.77560500	-0.16409600
H	3.83460300	1.30486500	-1.73858400

H	3.24381600	2.75241800	-0.88839400
N	-0.68365600	-2.00241200	-0.52082200
C	-1.42066800	-1.25894200	-1.49413000
H	-0.71021100	-0.57329600	-1.99310000
H	-1.83312400	-1.96486500	-2.23555400
C	-2.58490200	-0.48745600	-0.86665300
H	-3.27859500	-1.19860400	-0.38492600
H	-3.14242500	0.01797300	-1.67423600
C	-2.15083200	0.54670000	0.15745200
H	-1.62111700	0.08976800	1.00410500
H	-1.48953700	1.31557200	-0.26425500
Cl	-3.61922800	1.36544800	0.84088200

3p-2-reac

C	0.48035300	-0.86911300	0.09257800
S	1.96530200	-0.54951100	-0.93591200
S	0.40652300	-0.29950500	1.71745600
C	3.01436100	0.53531200	0.08836700
H	2.91068800	0.15635100	1.11699900
C	2.53446900	1.98178800	0.05064200
H	1.50492600	2.02887000	0.44000200
H	3.17832300	2.62561200	0.68091400
H	2.55203900	2.36873800	-0.98414200
C	4.45648300	0.39486600	-0.39273400
H	5.12825200	1.02555300	0.21801000
H	4.79606100	-0.65181500	-0.32568300
H	4.55483400	0.71782900	-1.44526500
N	-0.36739800	-1.54145600	-0.60089200
C	-1.60654500	-1.93654800	0.01641500
H	-1.87483200	-2.94587800	-0.35076600
H	-1.52905000	-1.97857900	1.12059600
C	-2.76066100	-1.00079600	-0.37208800
H	-3.71325600	-1.39429300	0.02739200
H	-2.84575800	-0.97051800	-1.47229700
C	-2.55811100	0.41313200	0.13603500
H	-2.48489700	0.45966300	1.22951600
H	-1.65414100	0.87120400	-0.28577100
Cl	-3.95662900	1.46763900	-0.35195600

3p-2-TS

C	0.60318600	0.93188600	-0.08312100
S	2.31692700	0.85255000	0.48729800
S	-0.00472100	-0.20097400	-1.25570100
C	2.92913200	-0.76006000	-0.11654700

H	2.57190000	-0.86097700	-1.15276900
C	2.38965400	-1.91793200	0.71418400
H	1.28985000	-1.93768100	0.65984700
H	2.77418700	-2.88170900	0.33067100
H	2.69585500	-1.80906800	1.76933300
C	4.45491000	-0.70333900	-0.09697000
H	4.87514100	-1.65629800	-0.46451200
H	4.83099900	0.11644700	-0.73044200
H	4.82816000	-0.54184500	0.93023500
N	-0.04927900	1.90010300	0.42778900
C	-1.39975700	2.07467000	-0.04382000
H	-1.79640600	3.01110000	0.38333200
H	-1.41913100	2.16330600	-1.14957000
C	-2.36067800	0.94033700	0.36159600
H	-3.39063300	1.27008200	0.16114800
H	-2.28733000	0.78048400	1.44854300
C	-2.19668900	-0.38994100	-0.33471100
H	-2.57259500	-0.52702800	-1.34430700
H	-1.83289800	-1.26394100	0.19182600
Cl	-4.33741000	-1.21426700	0.34487800

3p-2-prod

C	0.36850400	0.54730100	0.20746000
S	2.02201700	1.18111700	-0.12903600
S	0.07926900	-1.16201200	-0.21921200
C	3.06729700	-0.29914600	-0.39413900
H	2.53222300	-0.96194600	-1.09200200
C	3.35828500	-1.03854600	0.90735400
H	2.42287000	-1.37831300	1.37869600
H	3.99153300	-1.92408400	0.71352000
H	3.88740000	-0.37271600	1.61072000
C	4.34960100	0.18982000	-1.06497100
H	5.02651900	-0.66176900	-1.25268900
H	4.13263500	0.68718400	-2.02376700
H	4.87812100	0.90916300	-0.41454100
N	-0.44164000	1.38879700	0.66669000
C	-1.83303800	1.08199300	0.94936100
H	-2.15419900	1.74464100	1.77001200
H	-2.46931800	1.32250500	0.07273900
C	-2.12428900	-0.37256200	1.29391800
H	-3.22041100	-0.46292000	1.36625900
H	-1.63341600	-0.67083800	2.23928500
C	-1.71072800	-1.27908200	0.14731400
H	-2.32090000	-0.99885700	-0.73083900

H	-1.87814200	-2.34204800	0.38146400
Cl	-4.50457600	0.12376900	-0.90538300

4. NMR spectra of products 3-5

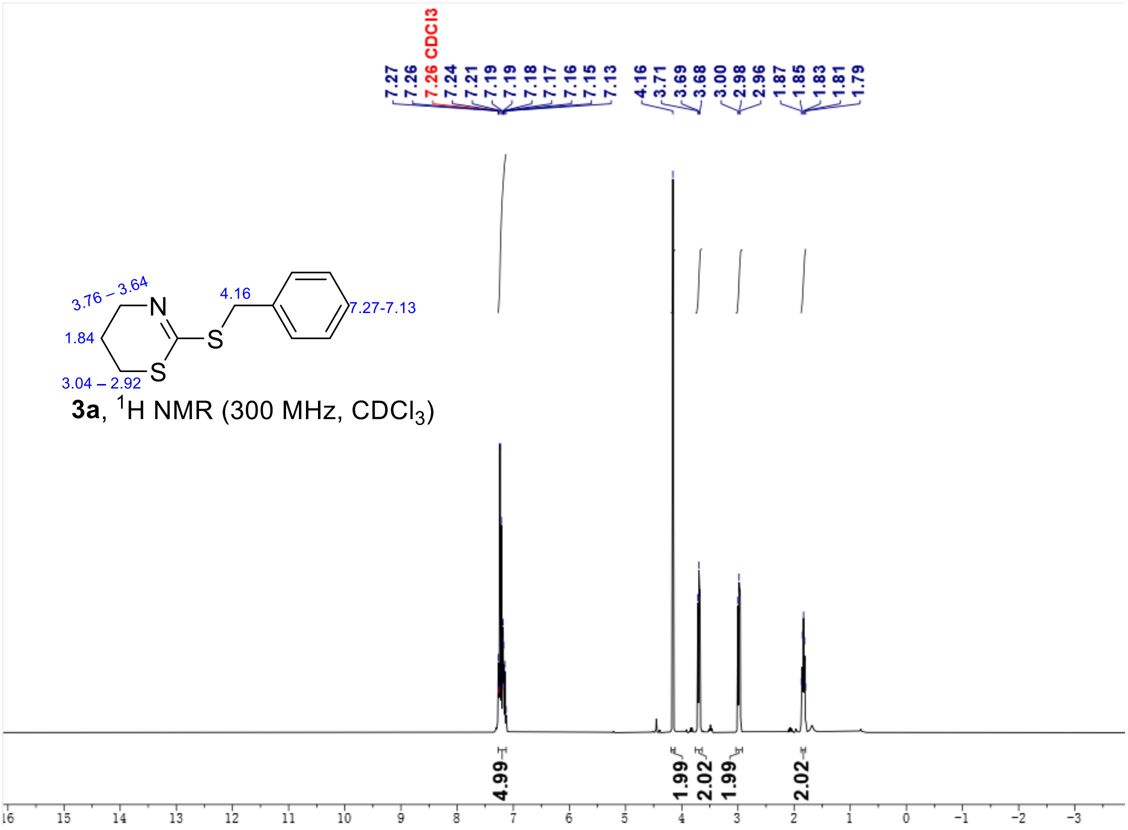

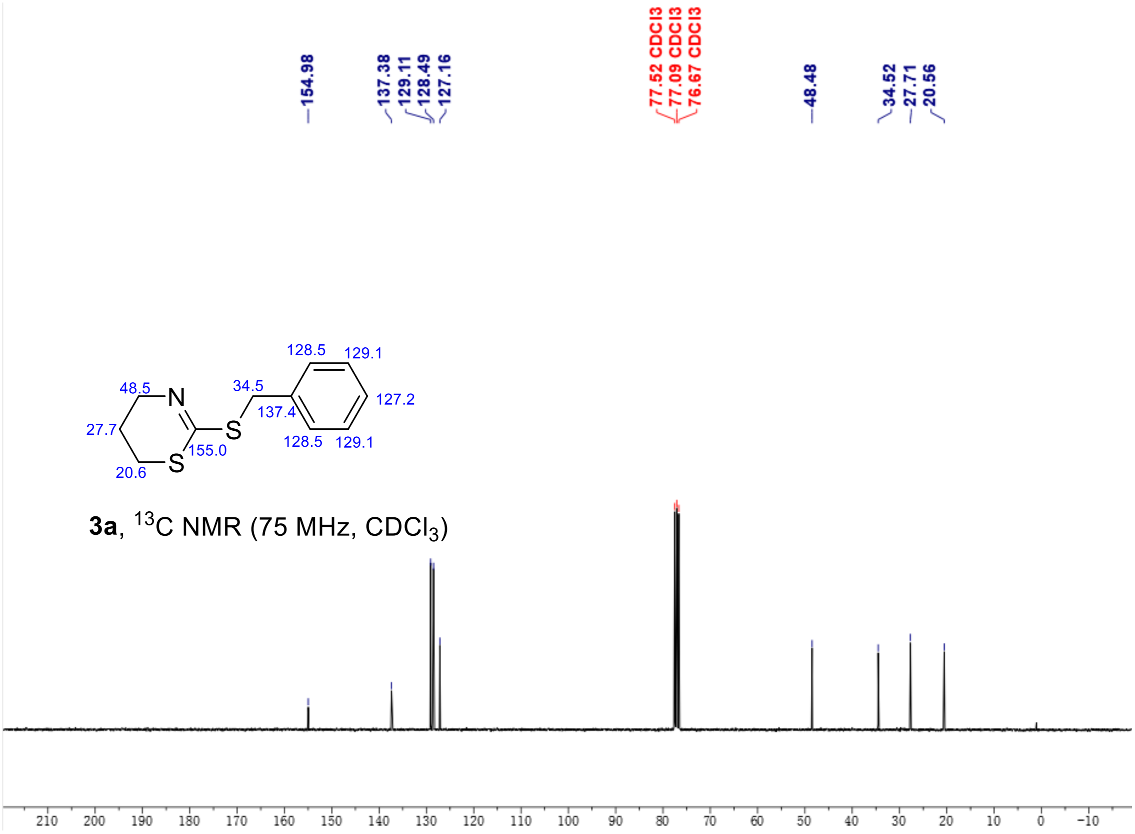

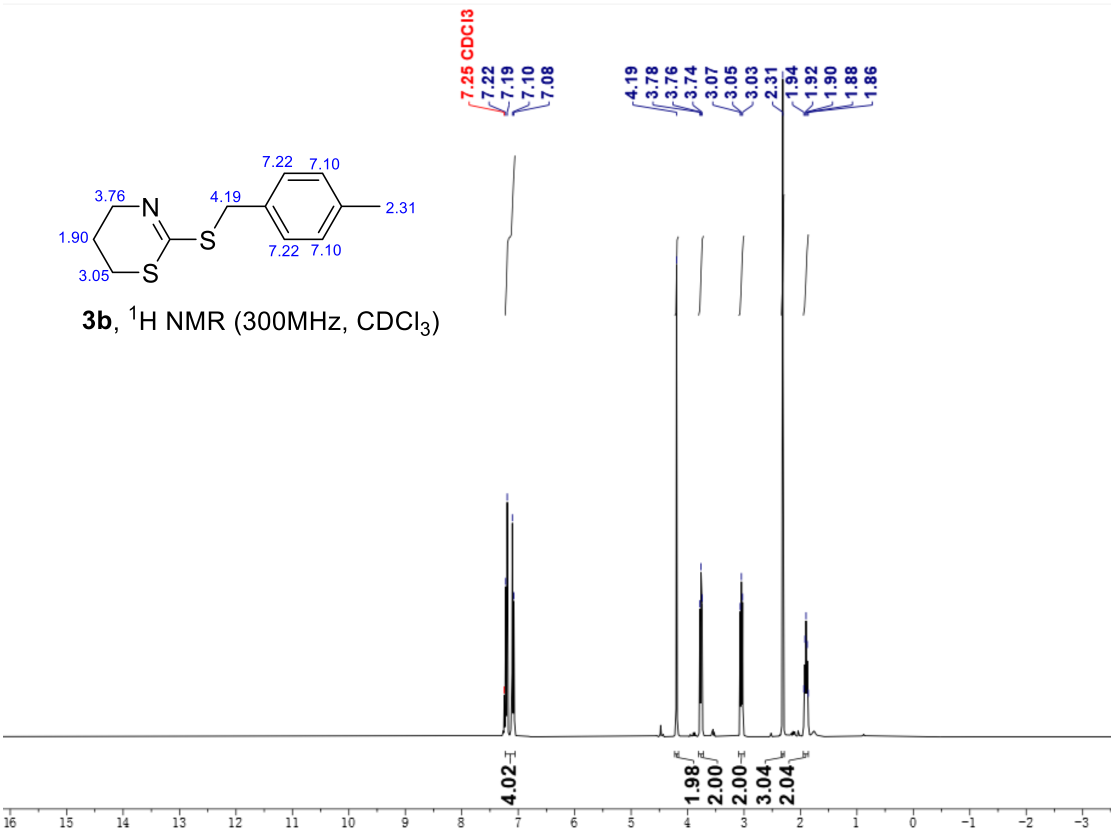
7.25 CDCl3
7.22
7.19
7.10
7.08
4.19
3.78
3.76
3.74
3.07
3.05
3.03
2.31
1.94
1.92
1.90
1.88
1.86
3b, 1H NMR (300MHz, CDCl3)
4.02
1.98
2.00
2.00
3.04
2.04

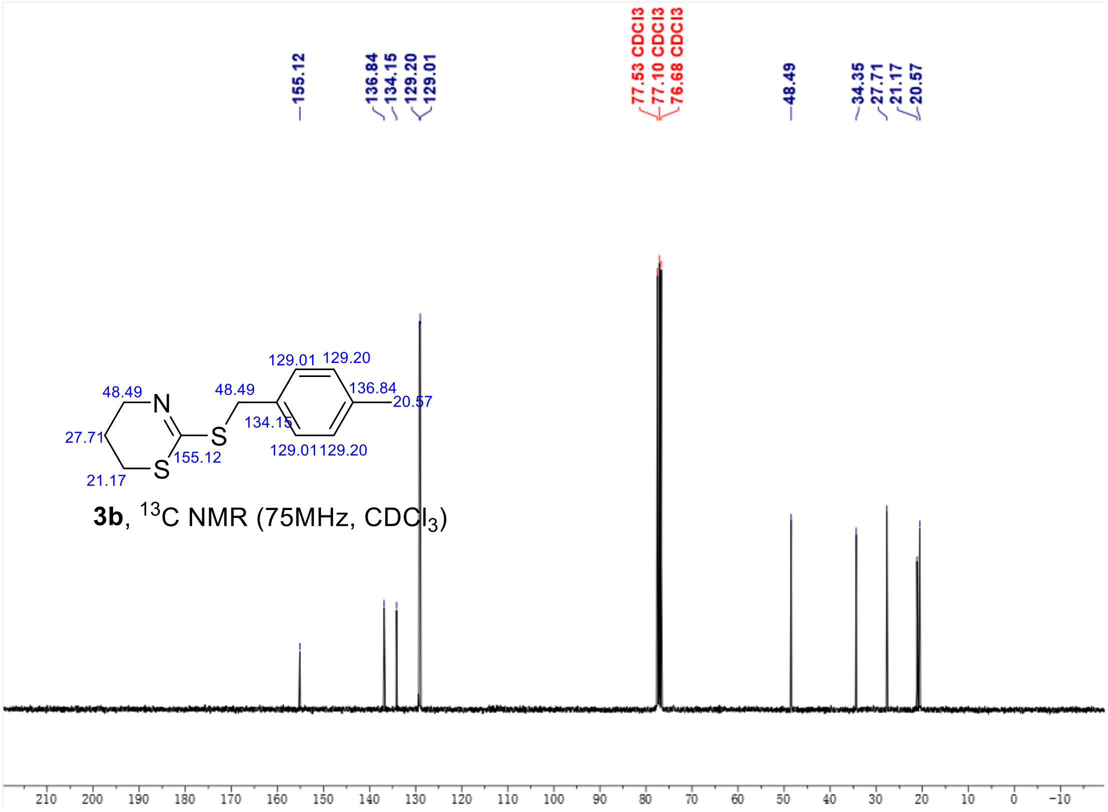
155.12
136.84
134.15
129.20
129.01
77.53 CDCl3
77.10 CDCl3
76.68 CDCl3
48.49
34.35
27.71
21.17
20.57
3b, 13C NMR (75MHz, CDCl3)

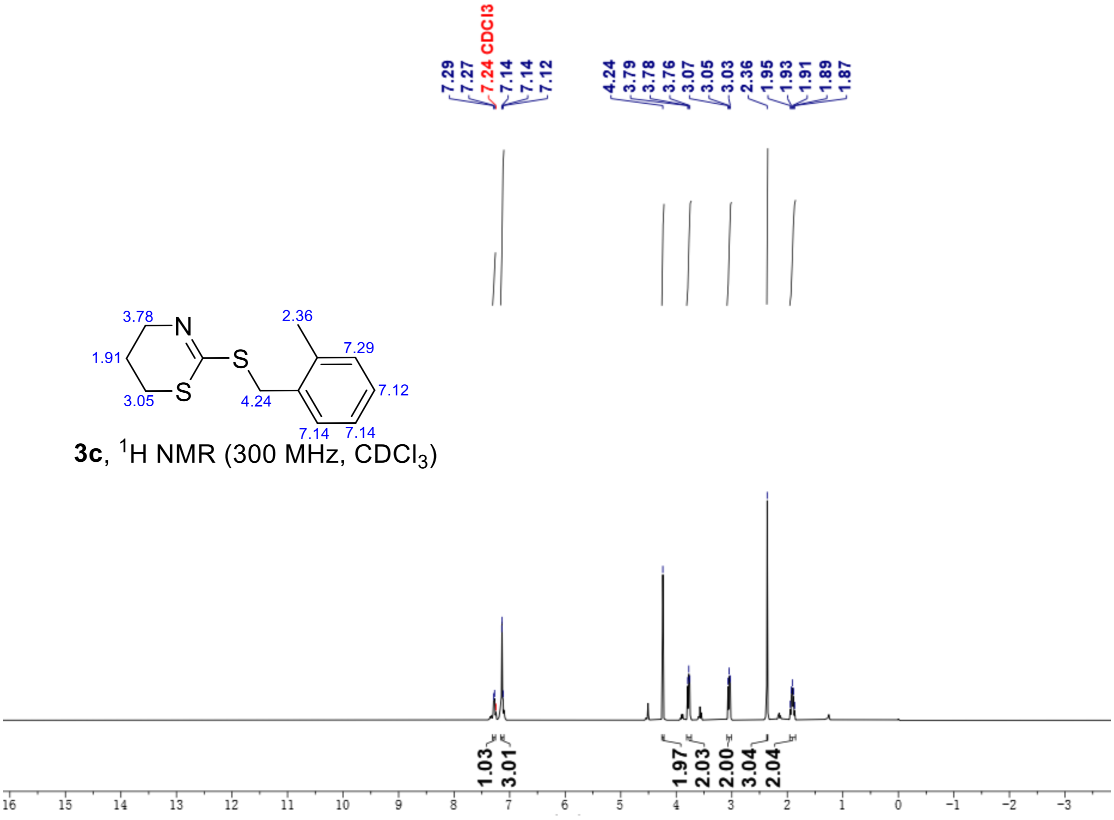

3c, ^{1}H NMR (300 MHz, $CDCl_3$)

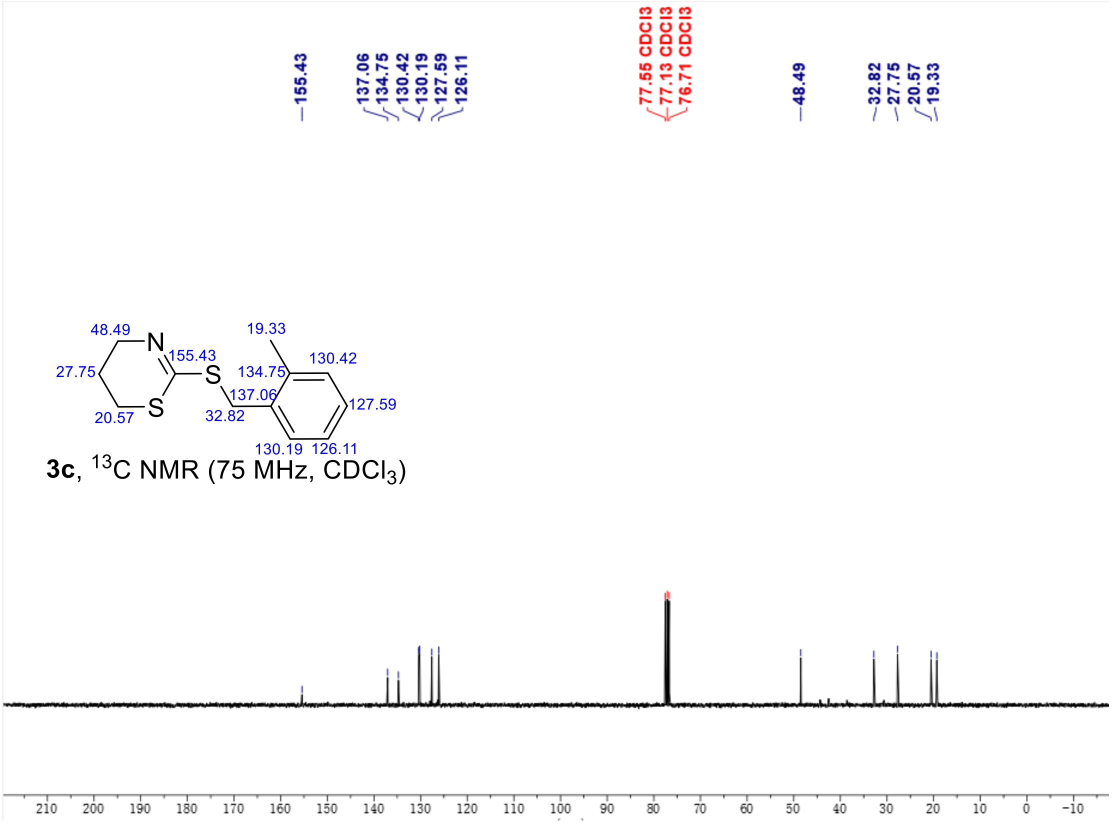

3c, ^{13}C NMR (75 MHz, $CDCl_3$)

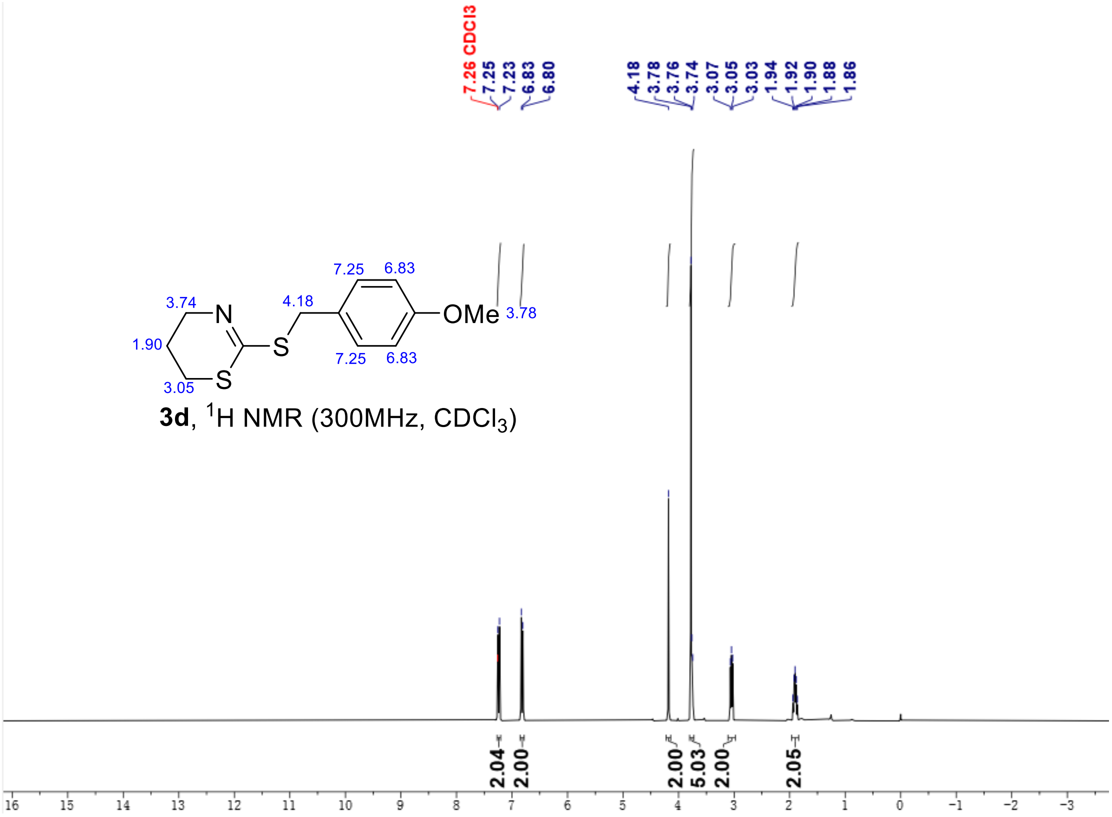
7.26 CDCl3
7.25
7.23
6.83
6.80
4.18
3.78
3.76
3.74
3.07
3.05
3.03
1.94
1.92
1.90
1.88
1.86
3.74
1.90
3.05
4.18
7.25
6.83
7.25
6.83
OMe 3.78
N
S
S
3d, ^{1}H NMR (300MHz, $CDCl_3$)
2.04
2.00
2.00
5.03
2.00
2.05
16 15 14 13 12 11 10 9 8 7 6 5 4 3 2 1 0 −1 −2 −3

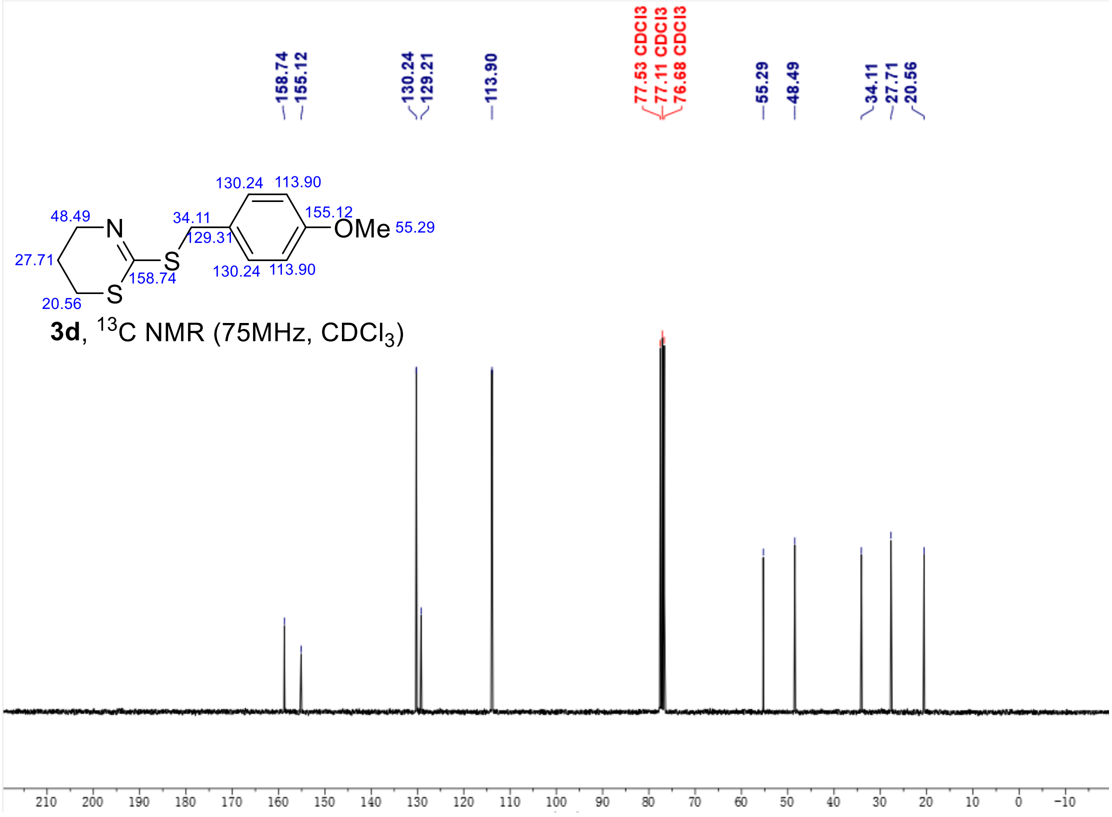
158.74
155.12
130.24
129.21
113.90
77.53 CDCl3
77.11 CDCl3
76.68 CDCl3
55.29
48.49
34.11
27.71
20.56
48.49
27.71
20.56
158.74
34.11
129.31
130.24
113.90
130.24
113.90
155.12
OMe 55.29
N
S
S
3d, ^{13}C NMR (75MHz, $CDCl_3$)
210 200 190 180 170 160 150 140 130 120 110 100 90 80 70 60 50 40 30 20 10 0 −10

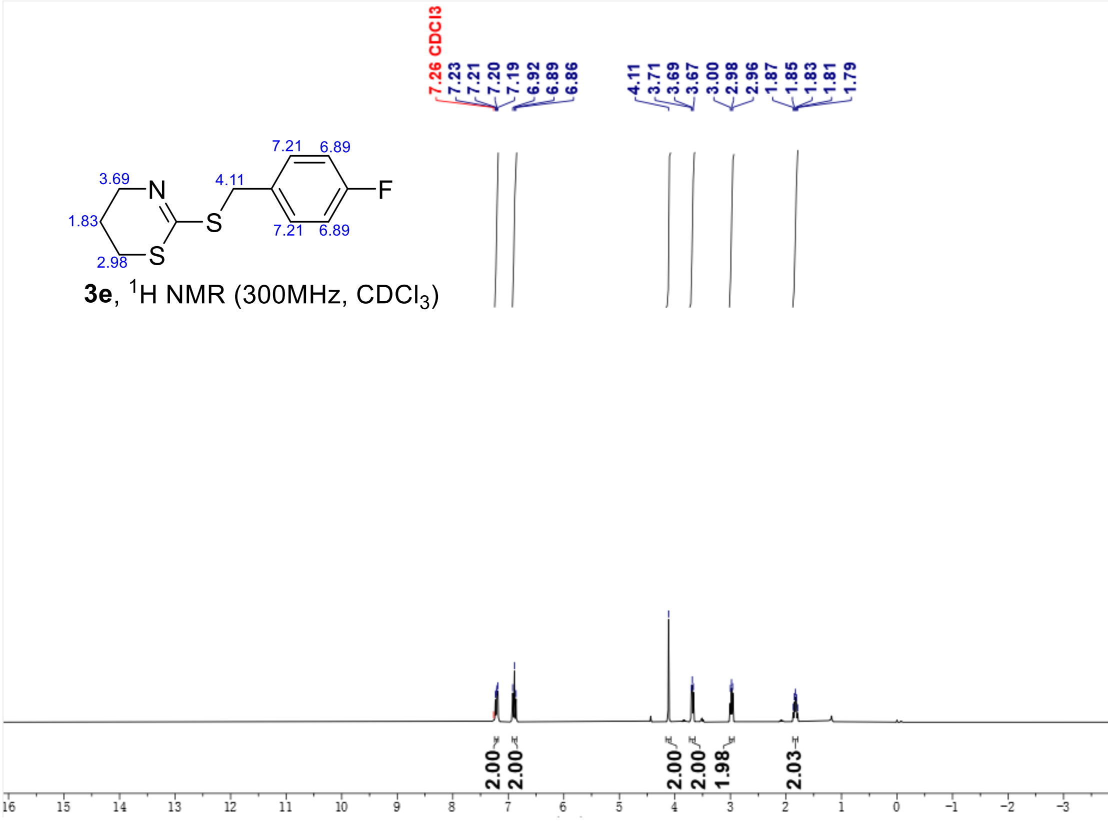
7.26 CDCl3
7.23
7.21
7.20
7.19
6.92
6.89
6.86
4.11
3.71
3.69
3.67
3.00
2.98
2.96
1.87
1.85
1.83
1.81
1.79
3e, 1H NMR (300MHz, CDCl3)
2.00
2.00
2.00
2.00
1.98
2.03
16 15 14 13 12 11 10 9 8 7 6 5 4 3 2 1 0 -1 -2 -3

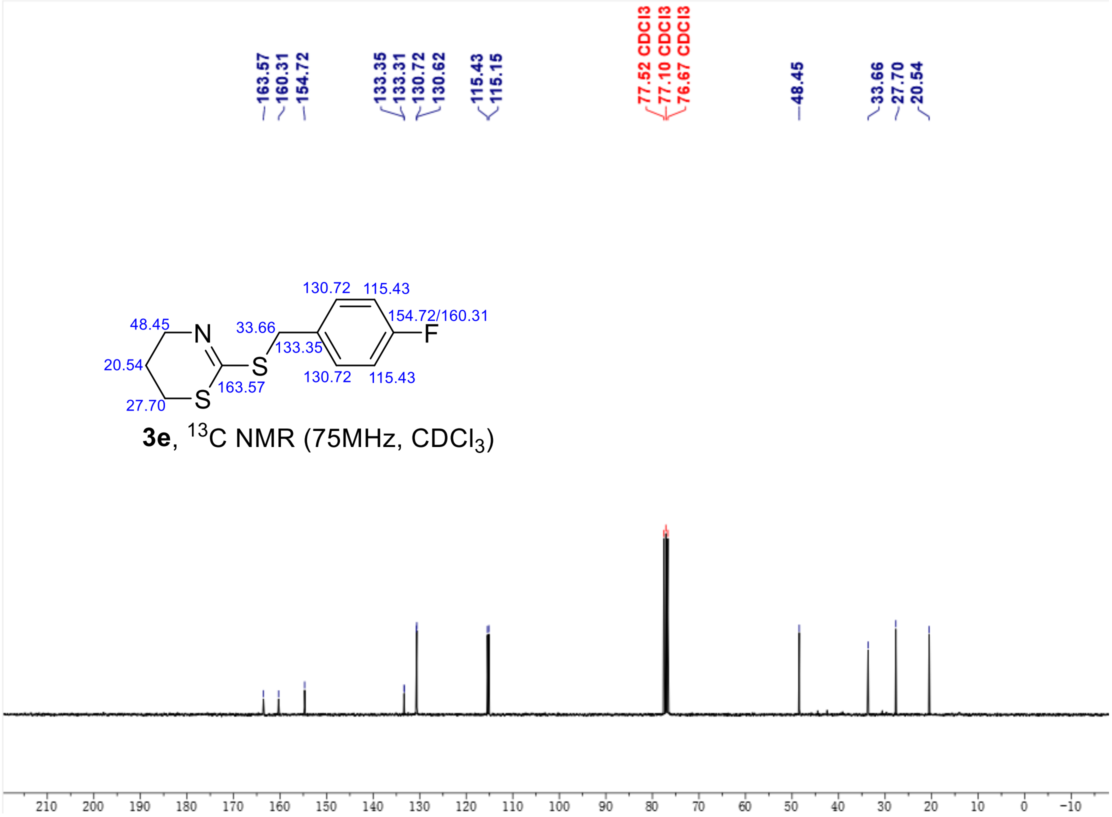
163.57
160.31
154.72
133.35
133.31
130.72
130.62
115.43
115.15
77.52 CDCl3
77.10 CDCl3
76.67 CDCl3
48.45
33.66
27.70
20.54
154.72/160.31
3e, 13C NMR (75MHz, CDCl3)
210 200 190 180 170 160 150 140 130 120 110 100 90 80 70 60 50 40 30 20 10 0 -10

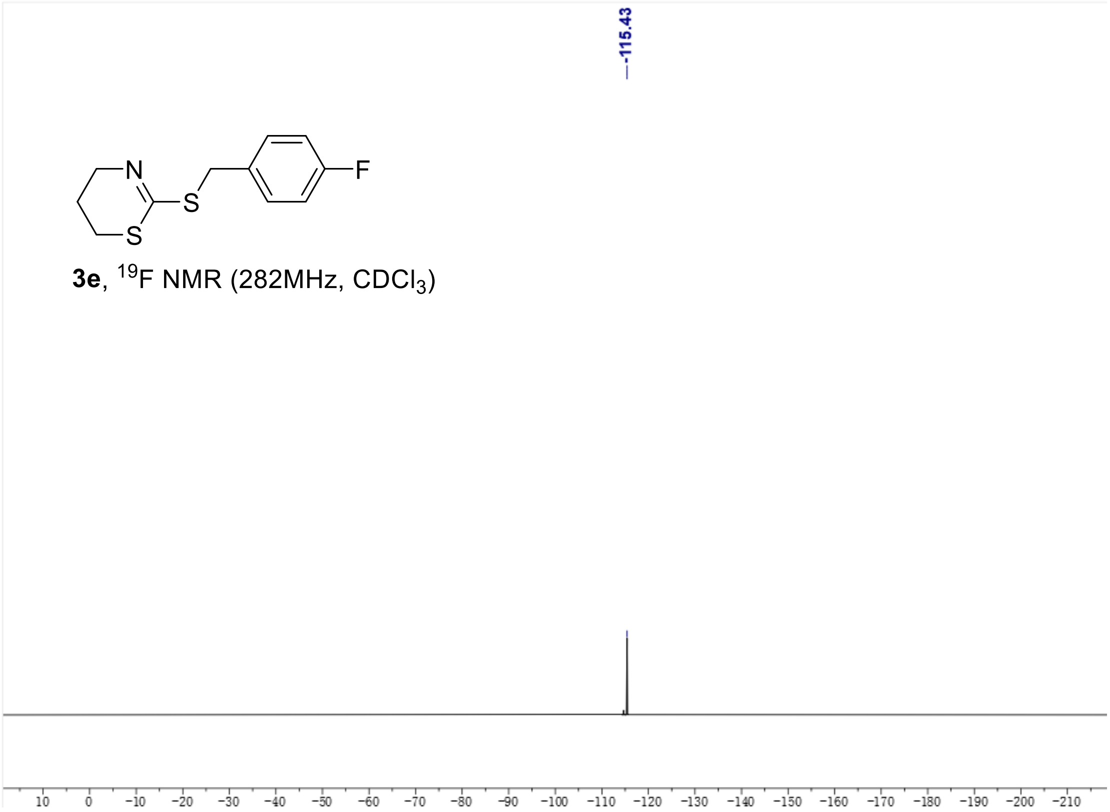

-115.43
N
S
S
F
3e, ¹⁹F NMR (282MHz, CDCl₃)
10
0
−10
−20
−30
−40
−50
−60
−70
−80
−90
−100
−110
−120
−130
−140
−150
−160
−170
−180
−190
−200
−210

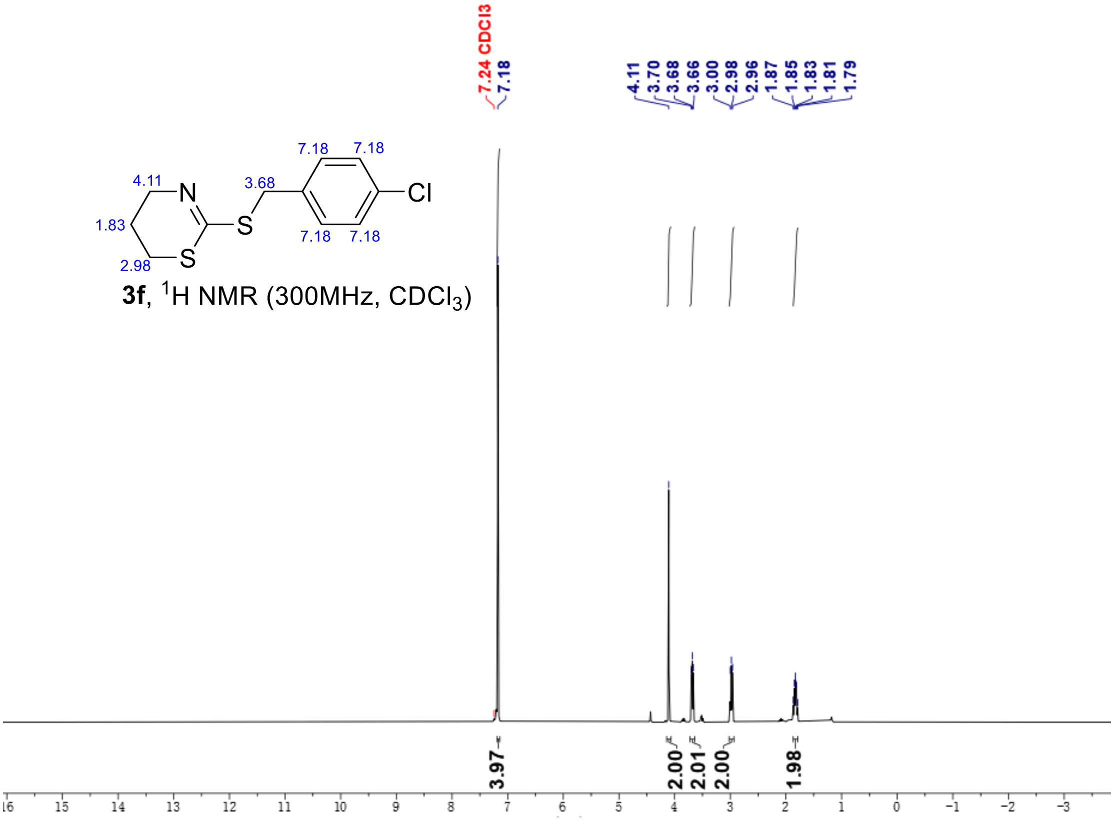

7.24 CDCl3
7.18
4.11
3.70
3.68
3.66
3.00
2.98
2.96
1.87
1.85
1.83
1.81
1.79
7.18 7.18
4.11
3.68
1.83
2.98
7.18 7.18
N
S
S
Cl
3f, ¹H NMR (300MHz, CDCl₃)
3.97
2.00
2.01
2.00
1.98
16
15
14
13
12
11
10
9
8
7
6
5
4
3
2
1
0
−1
−2
−3

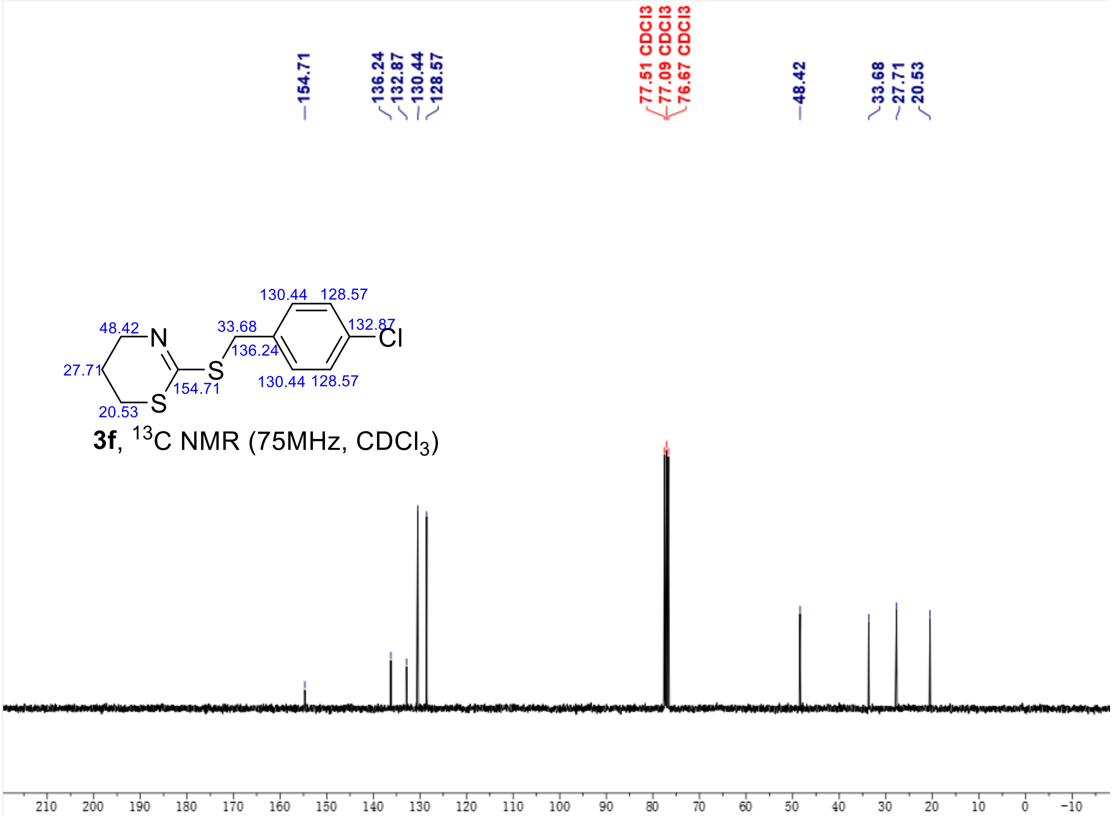

3f, ^{13}C NMR (75MHz, $CDCl_3$)

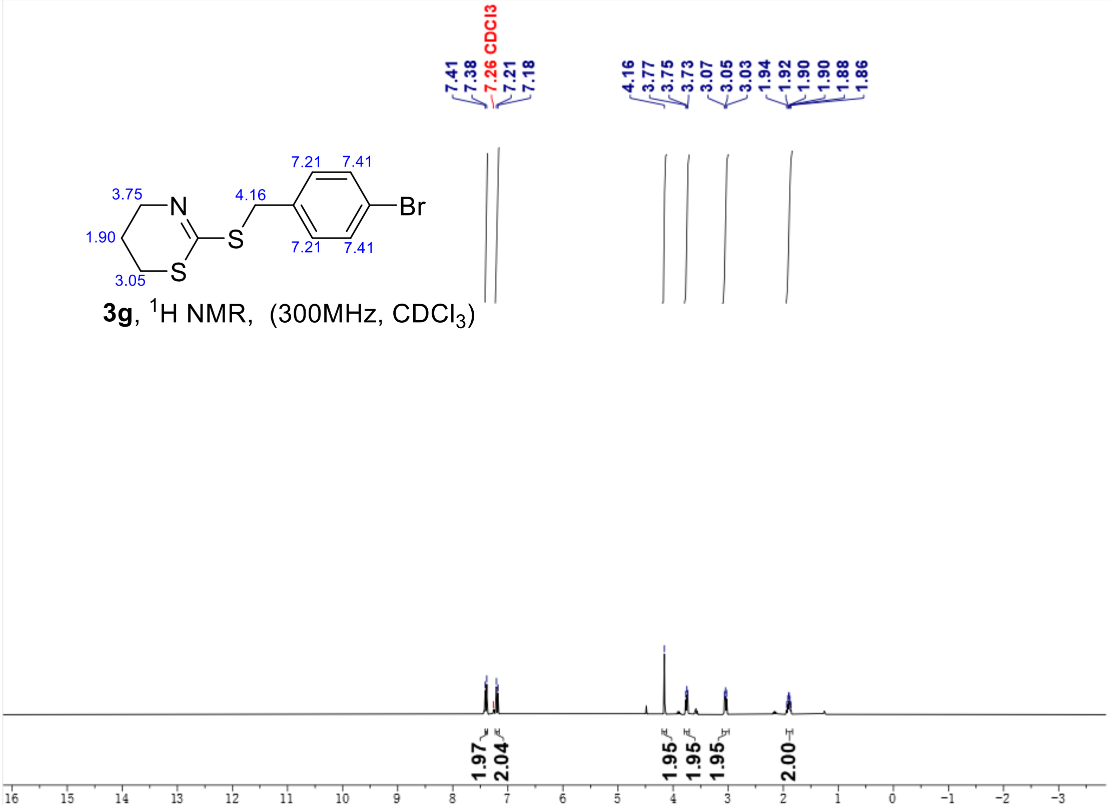

3g, ^{1}H NMR, (300MHz, $CDCl_3$)

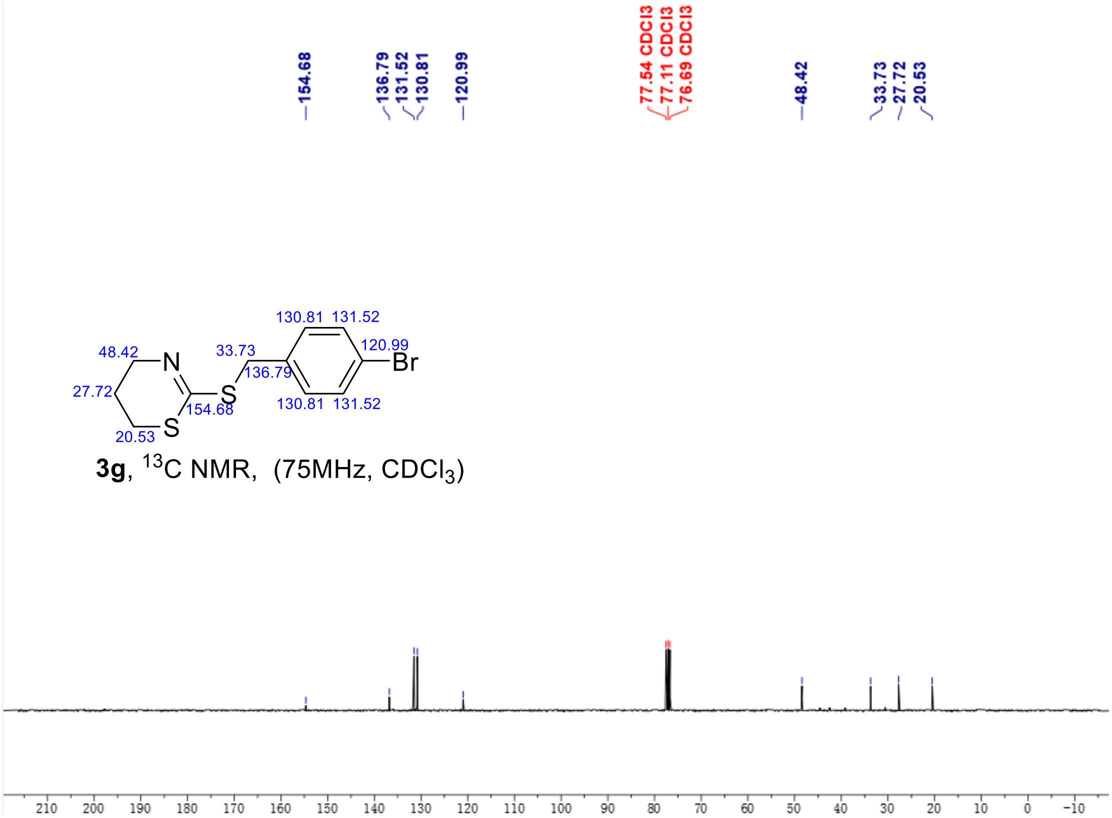

3g, ^{13}C NMR, (75MHz, $CDCl_3$)

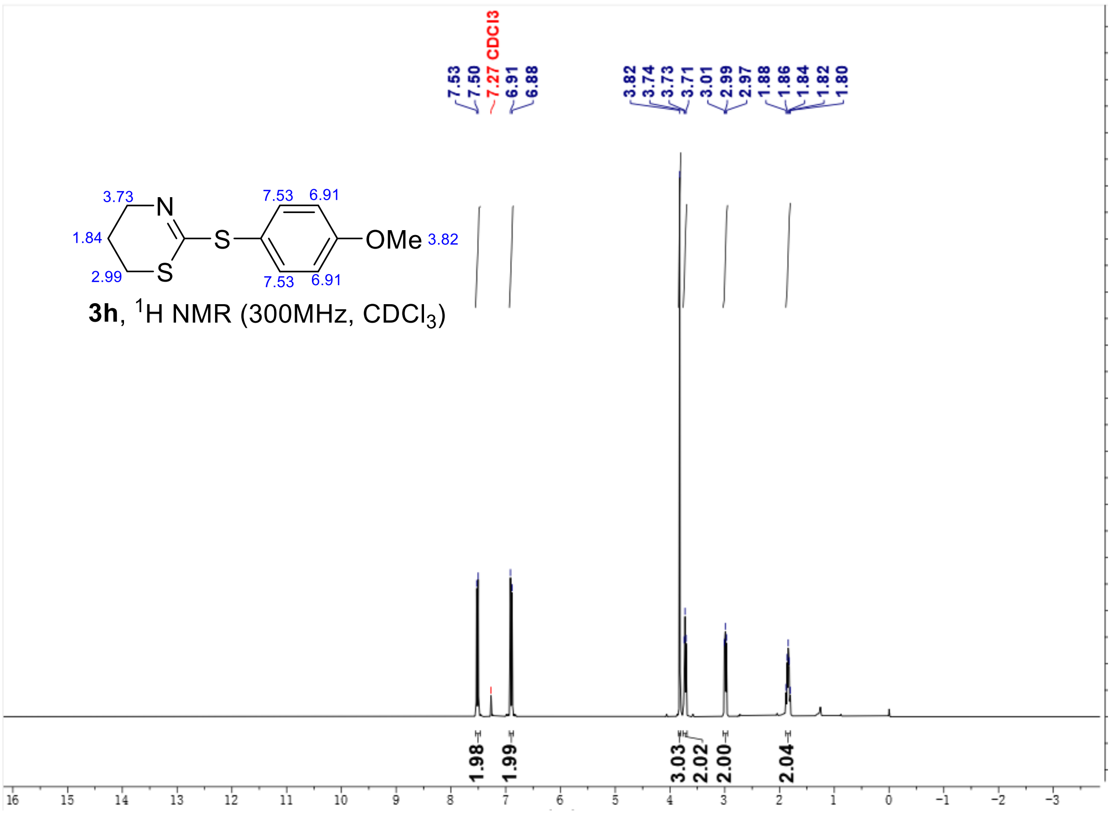

3h, ^{1}H NMR (300MHz, $CDCl_3$)

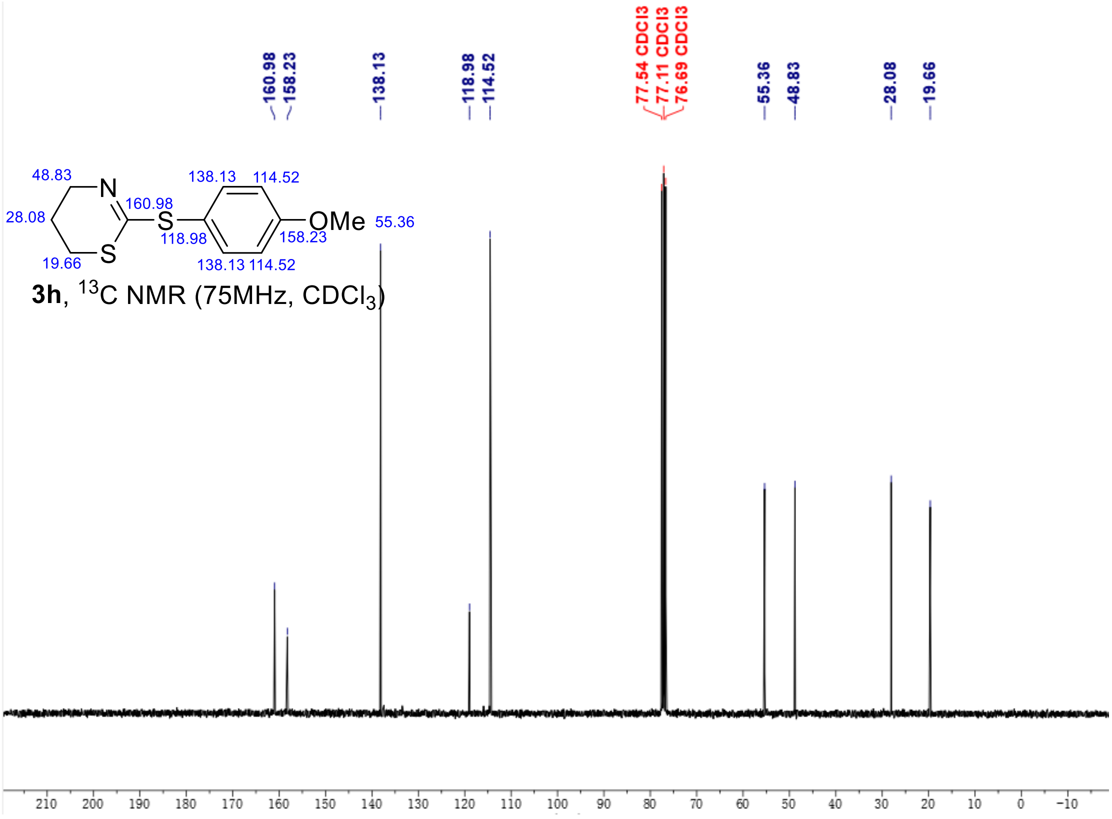

160.98
158.23
138.13
118.98
114.52
77.54 CDCl3
77.11 CDCl3
76.69 CDCl3
55.36
48.83
28.08
19.66
48.83
28.08
19.66
N
S
160.98
S
118.98
138.13
114.52
138.13 114.52
OMe
55.36
158.23
3h, 13C NMR (75MHz, CDCl3)

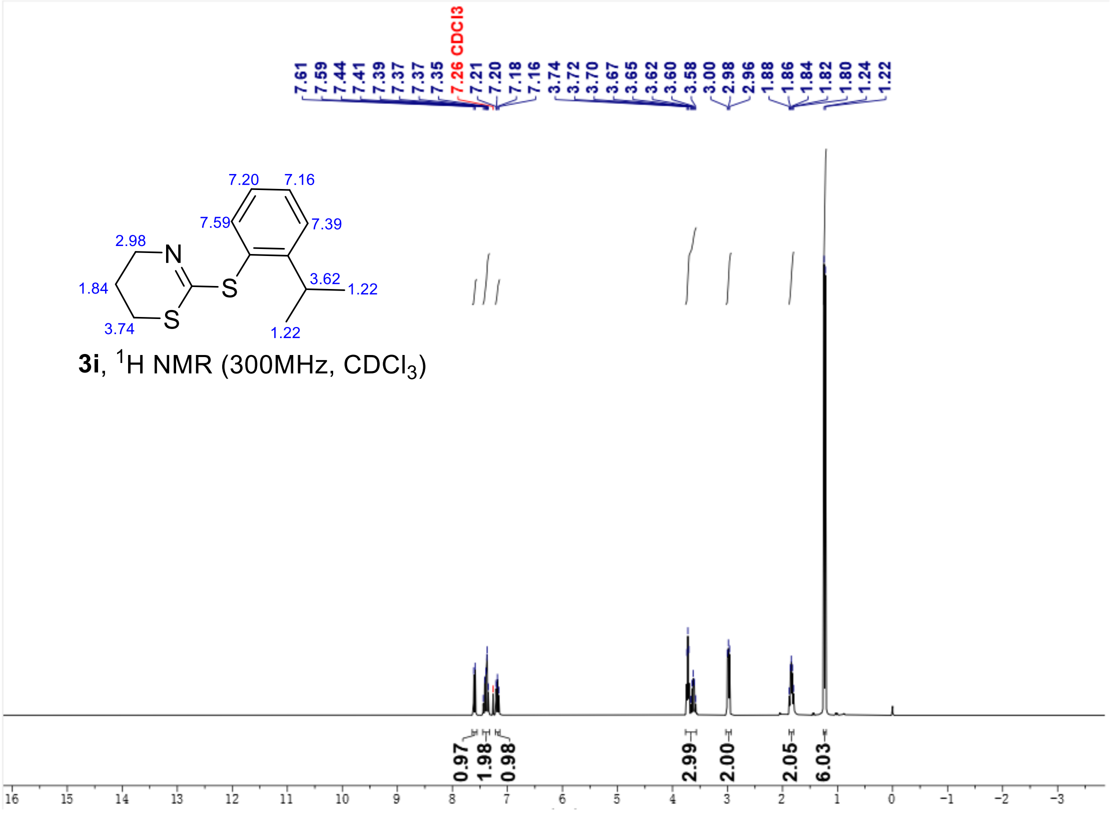

7.61
7.59
7.44
7.41
7.39
7.37
7.37
7.35
7.26 CDCl3
7.21
7.20
7.18
7.16
3.74
3.72
3.70
3.67
3.65
3.62
3.60
3.58
3.00
2.98
2.96
1.88
1.86
1.84
1.82
1.80
1.24
1.22
7.20
7.16
7.59
7.39
2.98
N
1.84
S
3.62
1.22
3.74
S
1.22
3i, 1H NMR (300MHz, CDCl3)
0.97
1.98
0.98
2.99
2.00
2.05
6.03

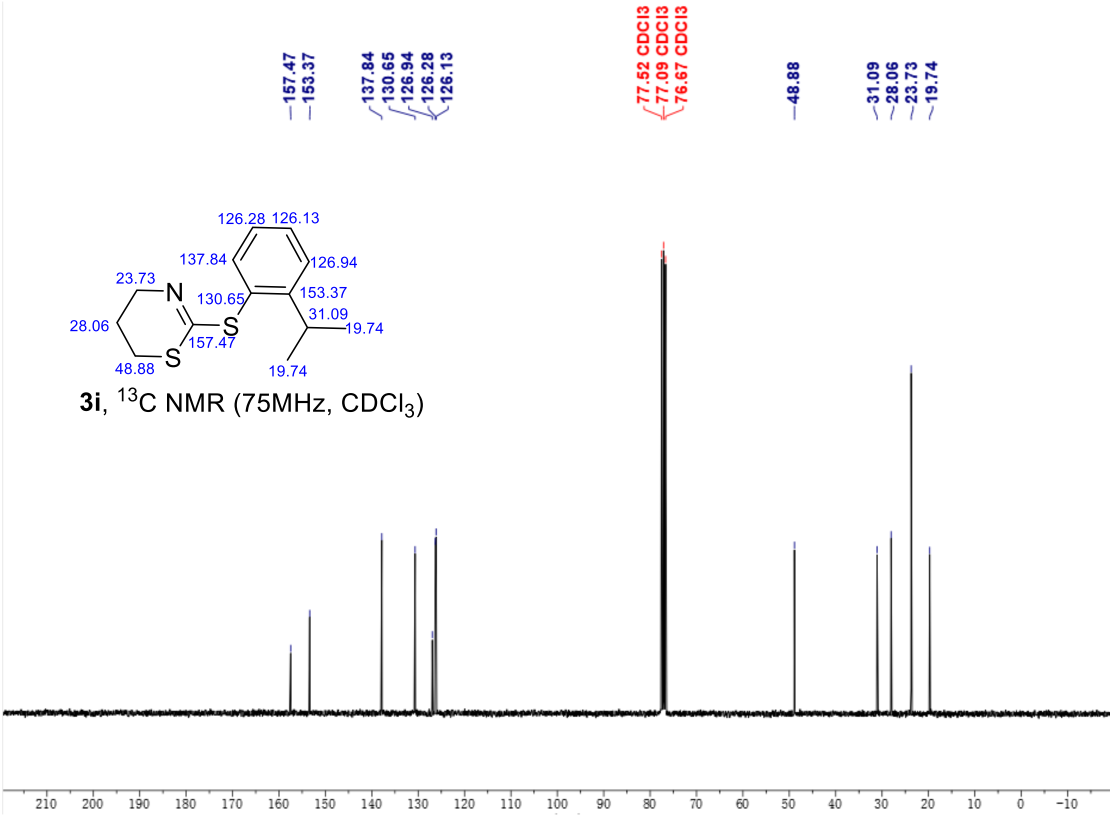
157.47
153.37
137.84
130.65
126.94
126.28
126.13
77.52 CDCl3
77.09 CDCl3
76.67 CDCl3
48.88
31.09
28.06
23.73
19.74
3i, ^{13}C NMR (75MHz, $CDCl_3$)

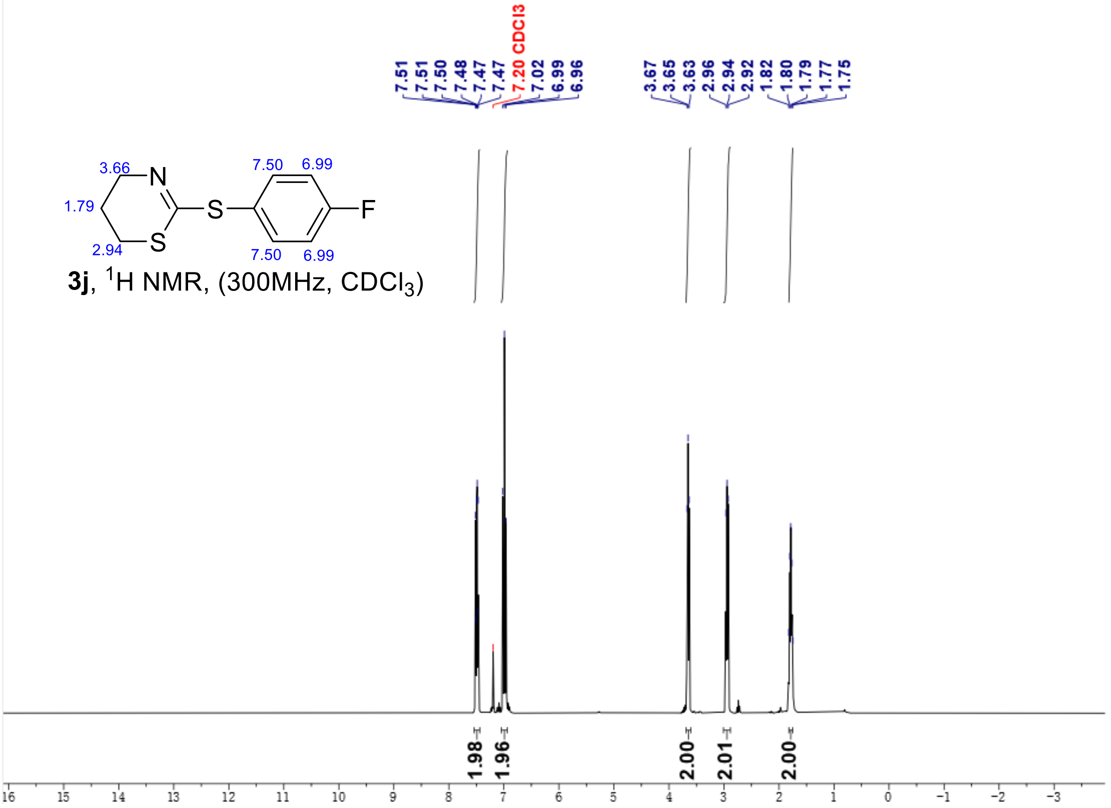
7.51
7.51
7.50
7.48
7.47
7.47
7.20 CDCl3
7.02
6.99
6.96
3.67
3.65
3.63
2.96
2.94
2.92
1.82
1.80
1.79
1.77
1.75
3j, ^{1}H NMR, (300MHz, $CDCl_3$)
1.98
1.96
2.00
2.01
2.00

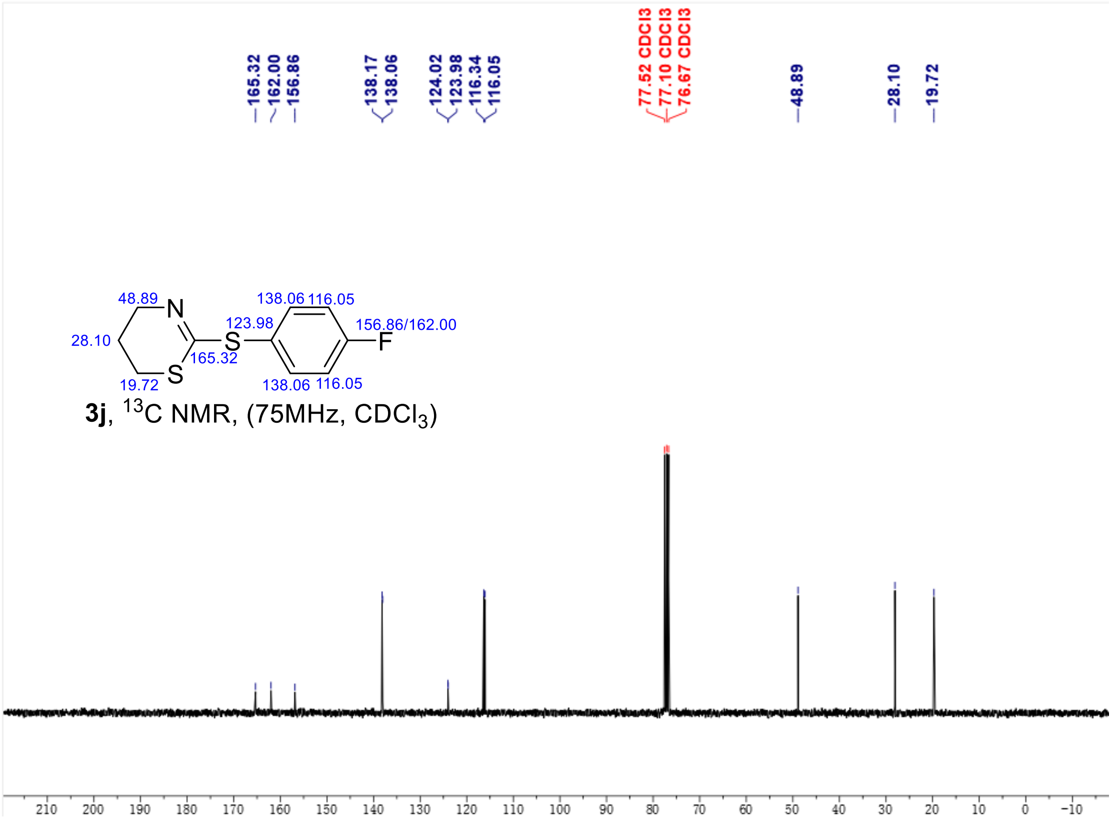
165.32
162.00
156.86
138.17
138.06
124.02
123.98
116.34
116.05
77.52 CDCl3
77.10 CDCl3
76.67 CDCl3
48.89
28.10
19.72
3j, 13C NMR, (75MHz, CDCl3)

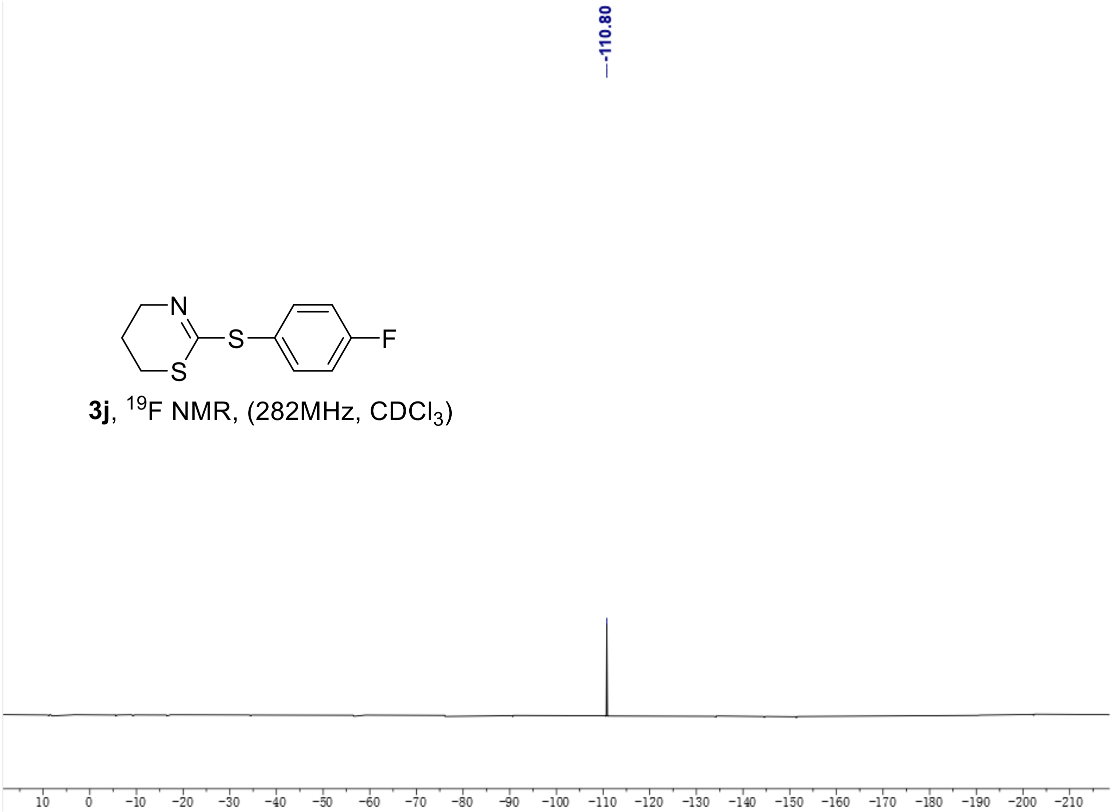
-110.80
3j, 19F NMR, (282MHz, CDCl3)

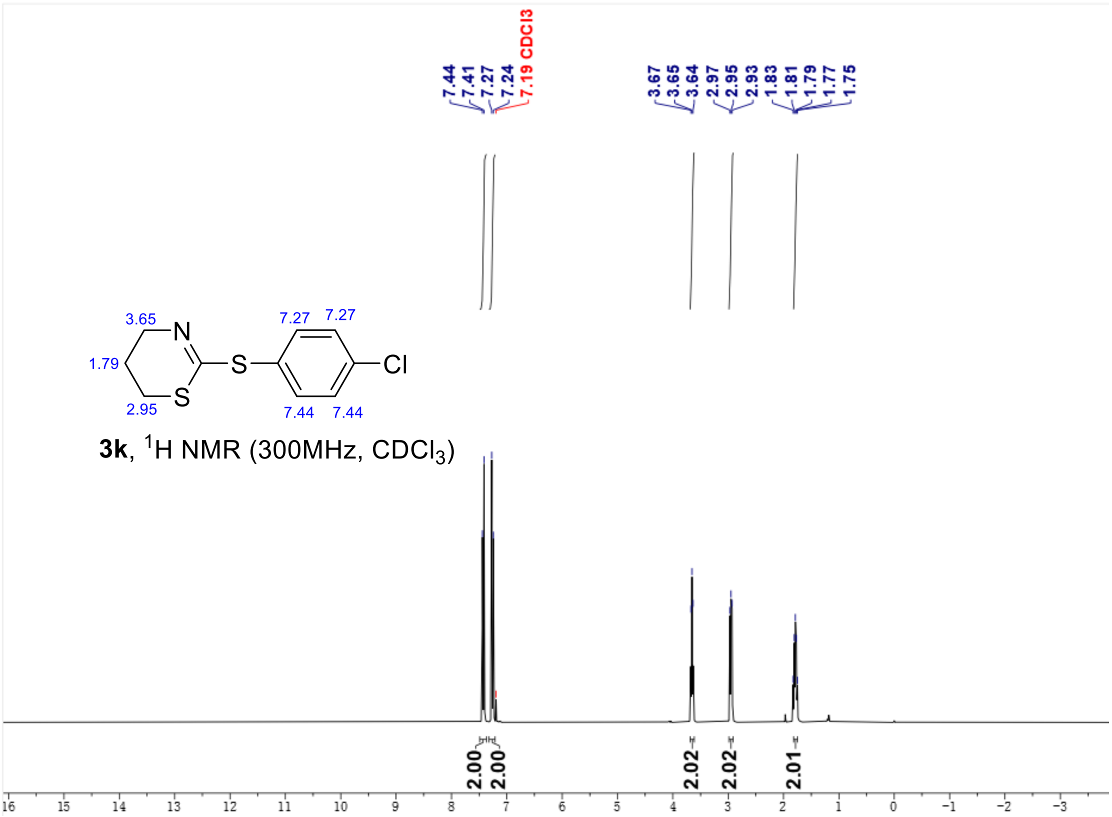
7.44
7.41
7.27
7.24
7.19 CDCl3
3.67
3.65
3.64
2.97
2.95
2.93
1.83
1.81
1.79
1.77
1.75
3.65
1.79
2.95
7.27
7.27
7.44
7.44
N
S
S
Cl
3k, ¹H NMR (300MHz, CDCl3)
2.00
2.00
2.02
2.02
2.01

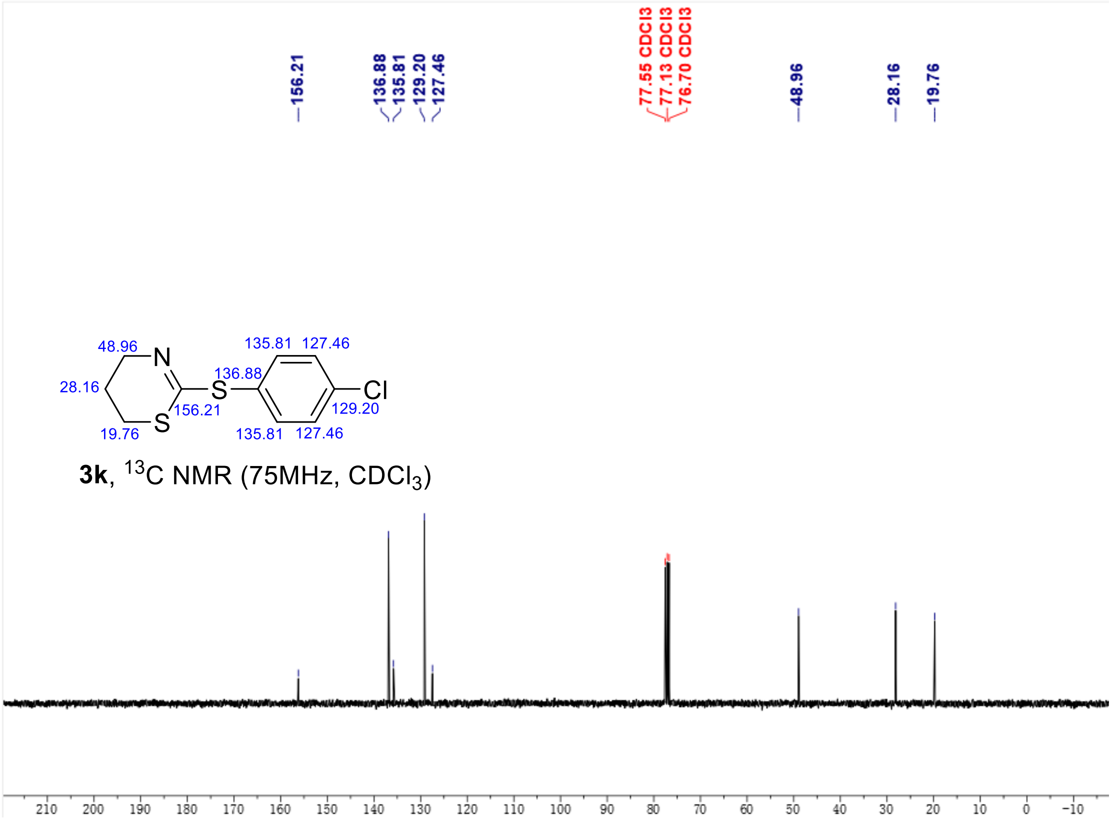
156.21
136.88
135.81
129.20
127.46
77.55 CDCl3
77.13 CDCl3
76.70 CDCl3
48.96
28.16
19.76
48.96
28.16
19.76
156.21
136.88
135.81
127.46
135.81
127.46
129.20
N
S
S
Cl
3k, ¹³C NMR (75MHz, CDCl3)

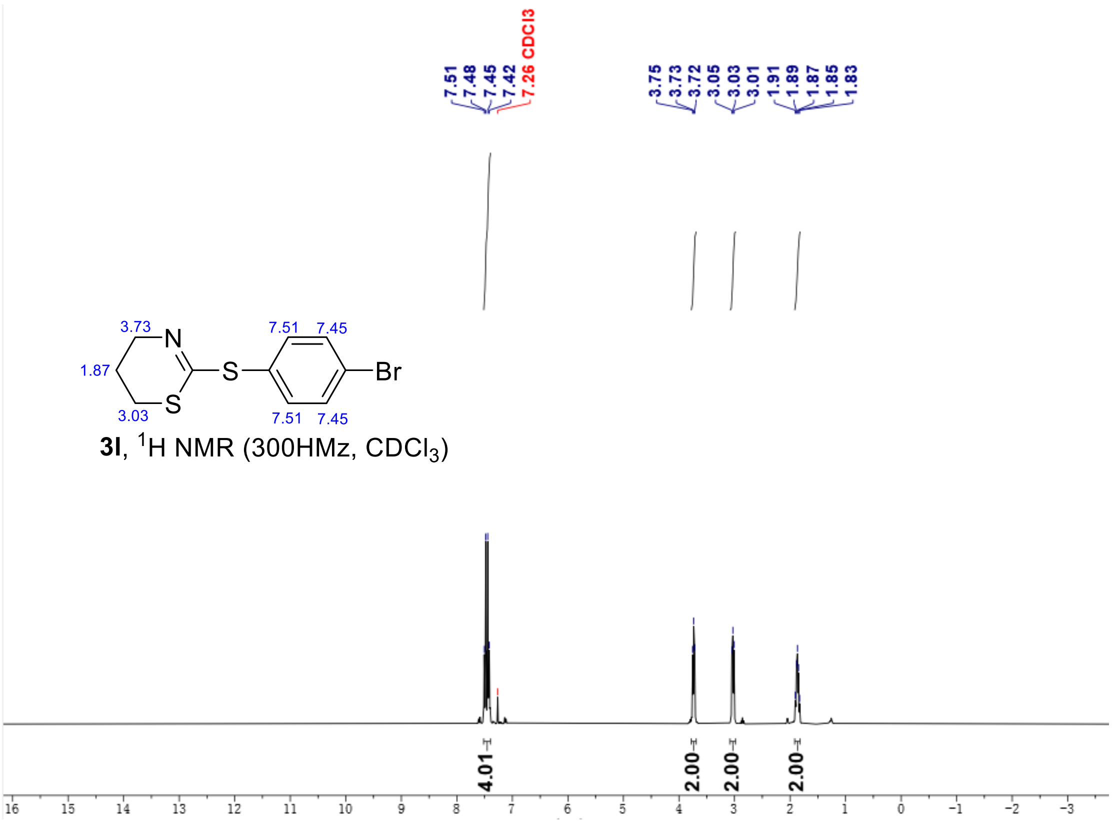
7.51
7.48
7.45
7.42
7.26 CDCl3
3.75
3.73
3.72
3.05
3.03
3.01
1.91
1.89
1.87
1.85
1.83
3.73
1.87
3.03
7.51
7.45
7.51
7.45
N
S
S
Br
3l, 1H NMR (300HMz, CDCl3)
4.01
2.00
2.00
2.00
16 15 14 13 12 11 10 9 8 7 6 5 4 3 2 1 0 -1 -2 -3

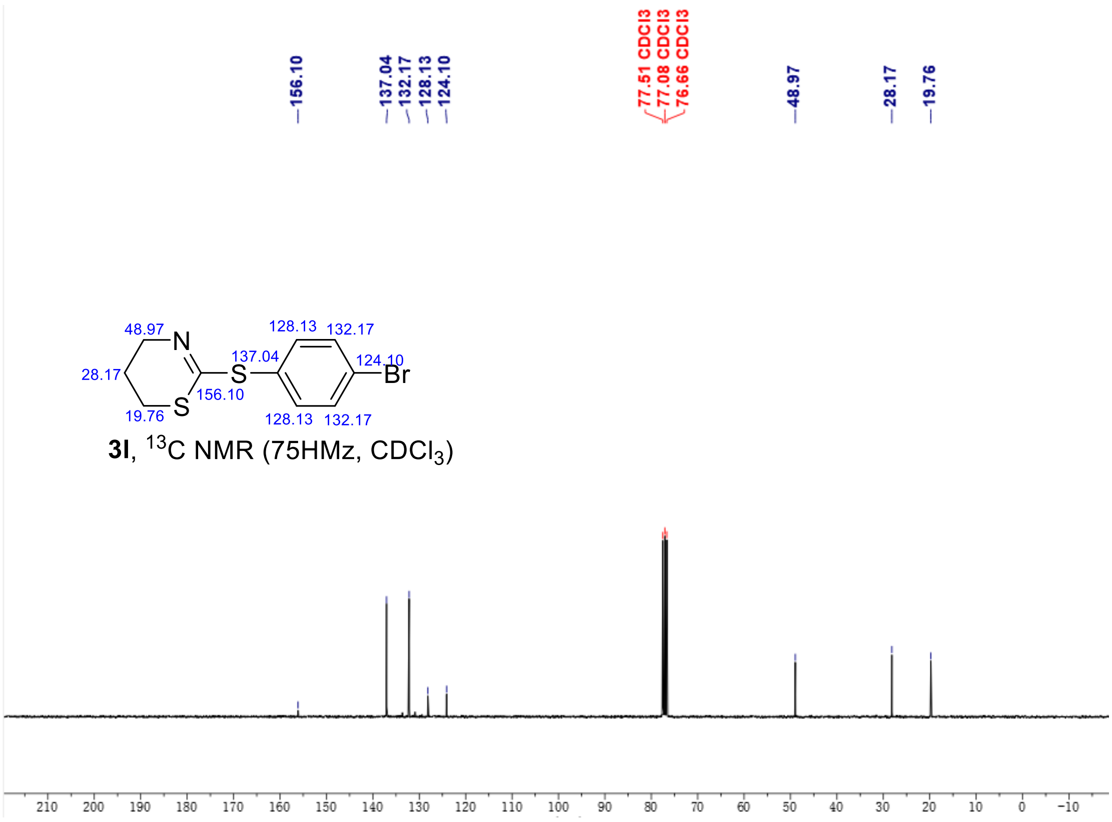
156.10
137.04
132.17
128.13
124.10
77.51 CDCl3
77.08 CDCl3
76.66 CDCl3
48.97
28.17
19.76
48.97
28.17
19.76
156.10
137.04
128.13
132.17
124.10
128.13
132.17
N
S
S
Br
3l, 13C NMR (75HMz, CDCl3)
210 200 190 180 170 160 150 140 130 120 110 100 90 80 70 60 50 40 30 20 10 0 -10

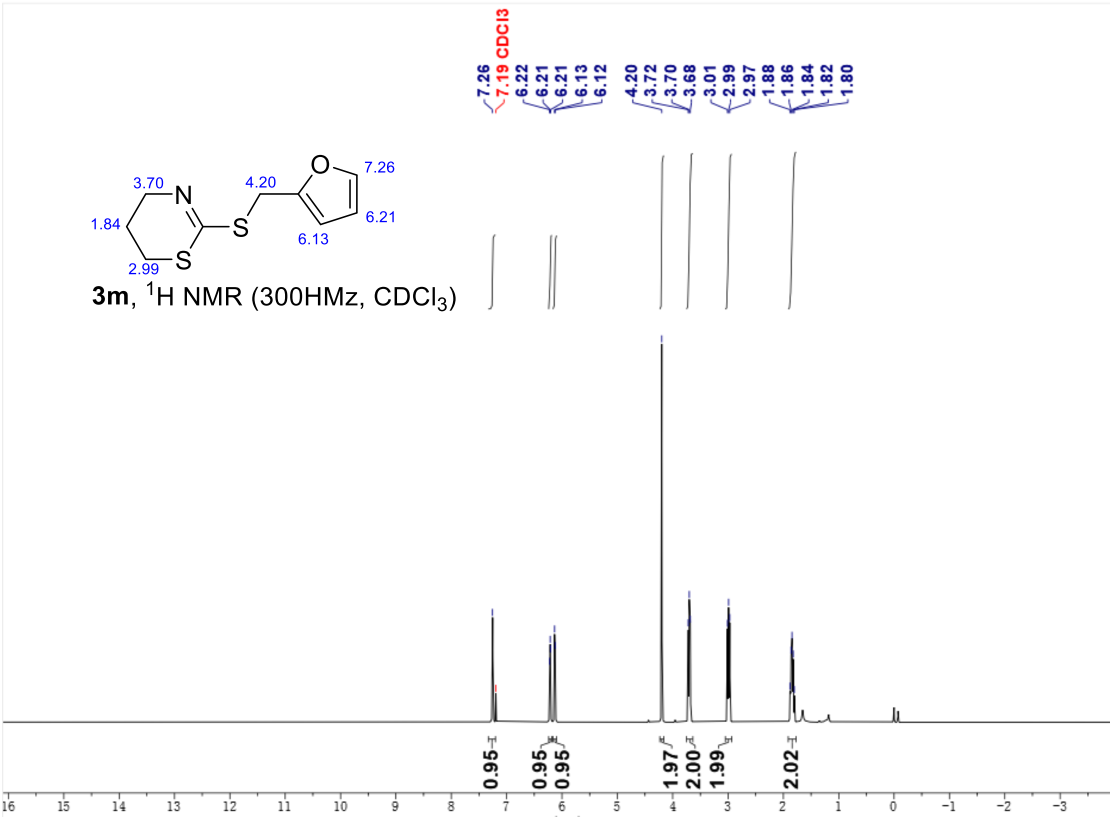
7.26
7.19 CDCl3
6.22
6.21
6.21
6.13
6.12
4.20
3.72
3.70
3.68
3.01
2.99
2.97
1.88
1.86
1.84
1.82
1.80
3.70
4.20
7.26
1.84
6.21
6.13
2.99
3m, 1H NMR (300HMz, CDCl3)
0.95
0.95
0.95
1.97
2.00
1.99
2.02
16
15
14
13
12
11
10
9
8
7
6
5
4
3
2
1
0
-1
-2
-3

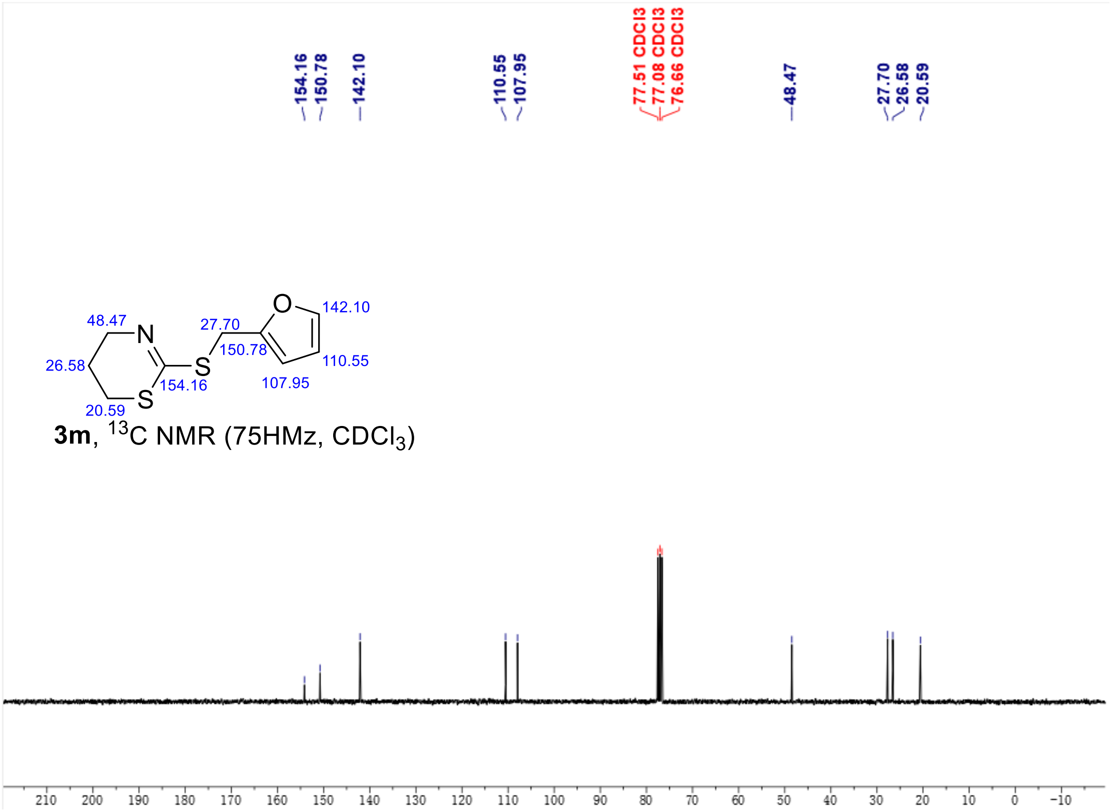
154.16
150.78
142.10
110.55
107.95
77.51 CDCl3
77.08 CDCl3
76.66 CDCl3
48.47
27.70
26.58
20.59
48.47
27.70
142.10
150.78
26.58
110.55
154.16
107.95
20.59
3m, 13C NMR (75HMz, CDCl3)
210
200
190
180
170
160
150
140
130
120
110
100
90
80
70
60
50
40
30
20
10
0
-10

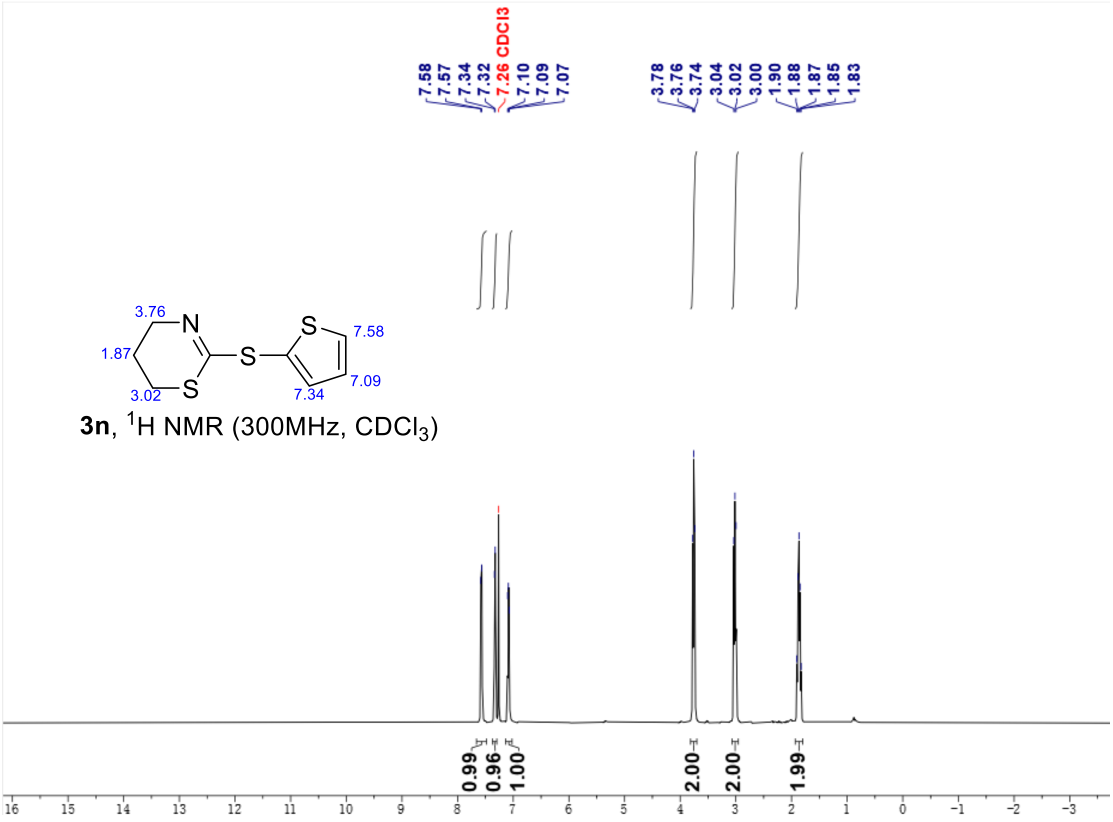
7.58
7.57
7.34
7.32
7.26 CDCl3
7.10
7.09
7.07
3.78
3.76
3.74
3.04
3.02
3.00
1.90
1.88
1.87
1.85
1.83
3.76
1.87
3.02
7.58
7.09
7.34
3n, 1H NMR (300MHz, CDCl3)
0.99
0.96
1.00
2.00
2.00
1.99

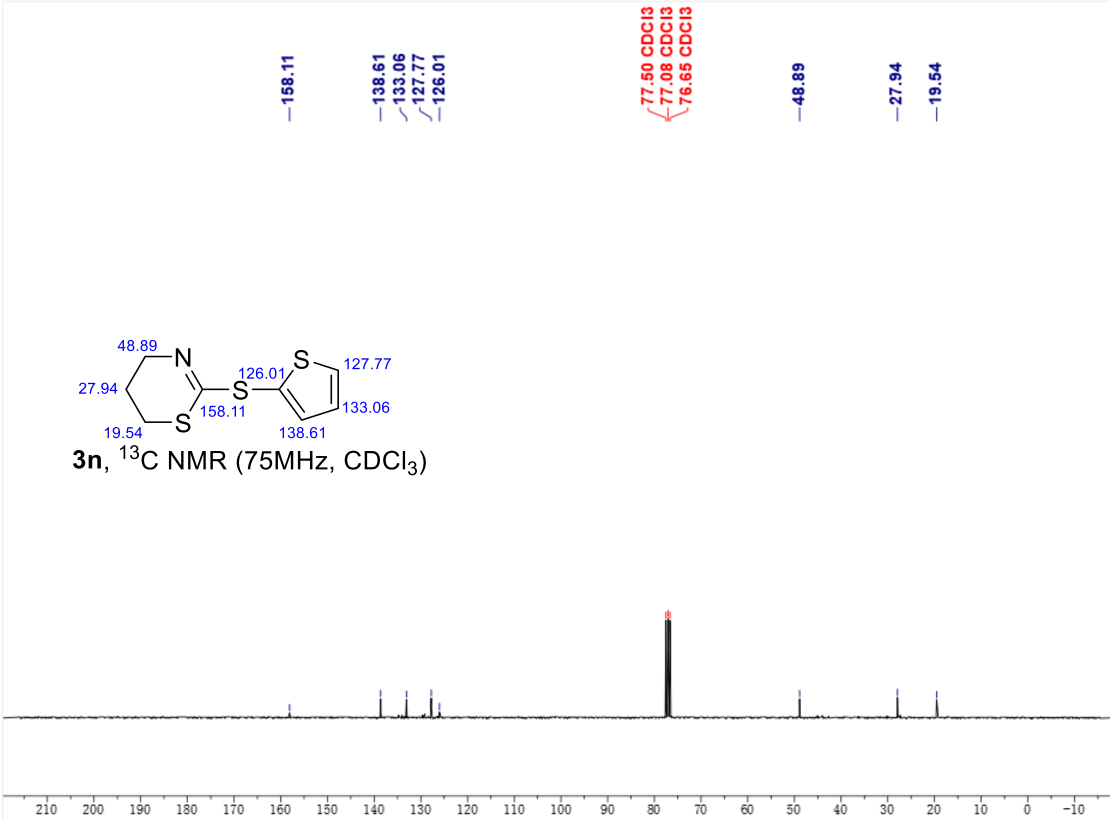
158.11
138.61
133.06
127.77
126.01
77.50 CDCl3
77.08 CDCl3
76.65 CDCl3
48.89
27.94
19.54
3n, 13C NMR (75MHz, CDCl3)

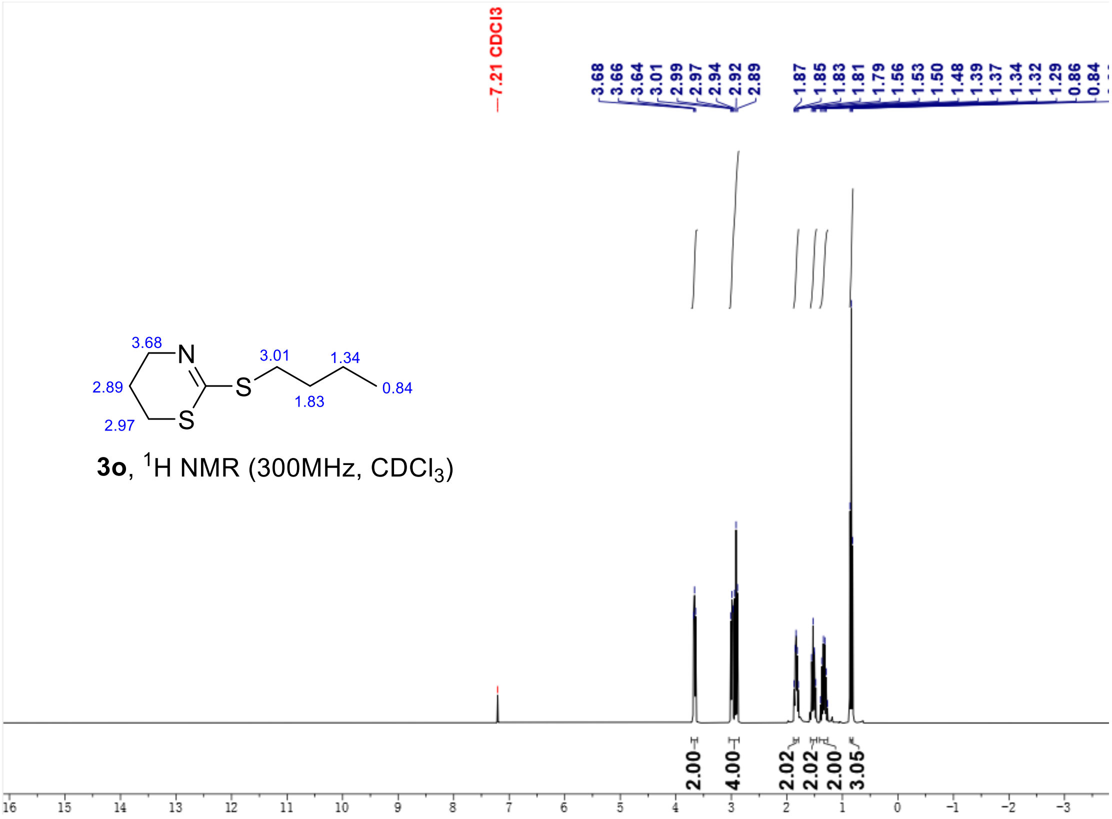
7.21 CDCl3
3.68
3.66
3.64
3.01
2.99
2.97
2.94
2.92
2.89
1.87
1.85
1.83
1.81
1.79
1.56
1.53
1.50
1.48
1.39
1.37
1.34
1.32
1.29
0.86
0.84
3.68
N
3.01
1.34
2.89
S
0.84
1.83
2.97
S
2.00
4.00
2.02
2.02
2.00
3.05
3o, 1H NMR (300MHz, CDCl3)

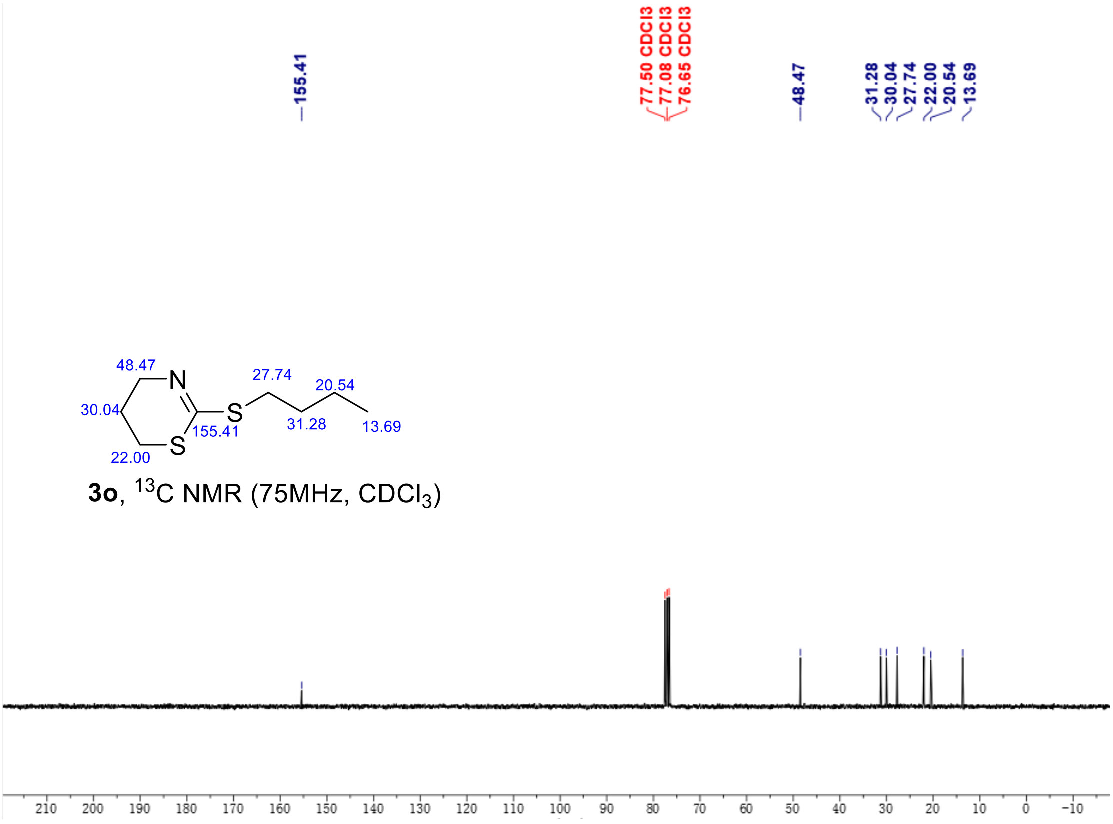
155.41
77.50 CDCl3
77.08 CDCl3
76.65 CDCl3
48.47
31.28
30.04
27.74
22.00
20.54
13.69
48.47
N
27.74
20.54
30.04
S
155.41
31.28
13.69
22.00
S
3o, 13C NMR (75MHz, CDCl3)

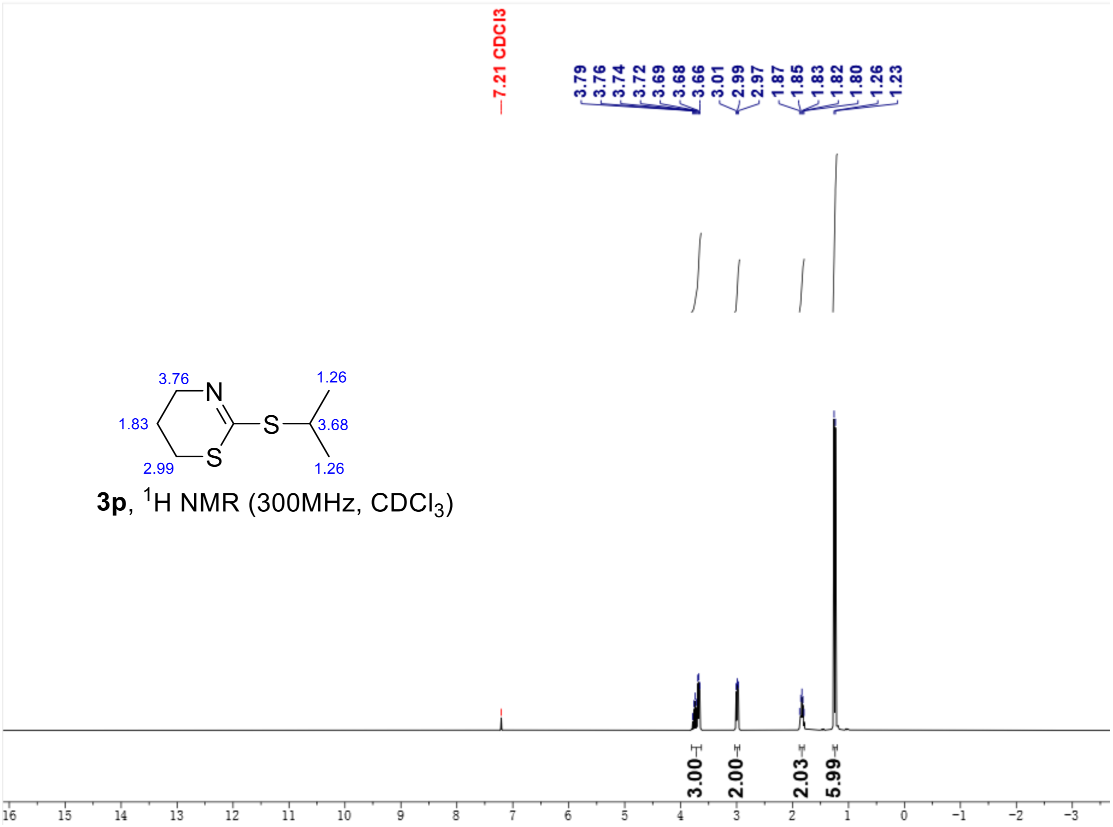

3p, ^{1}H NMR (300MHz, $CDCl_3$)

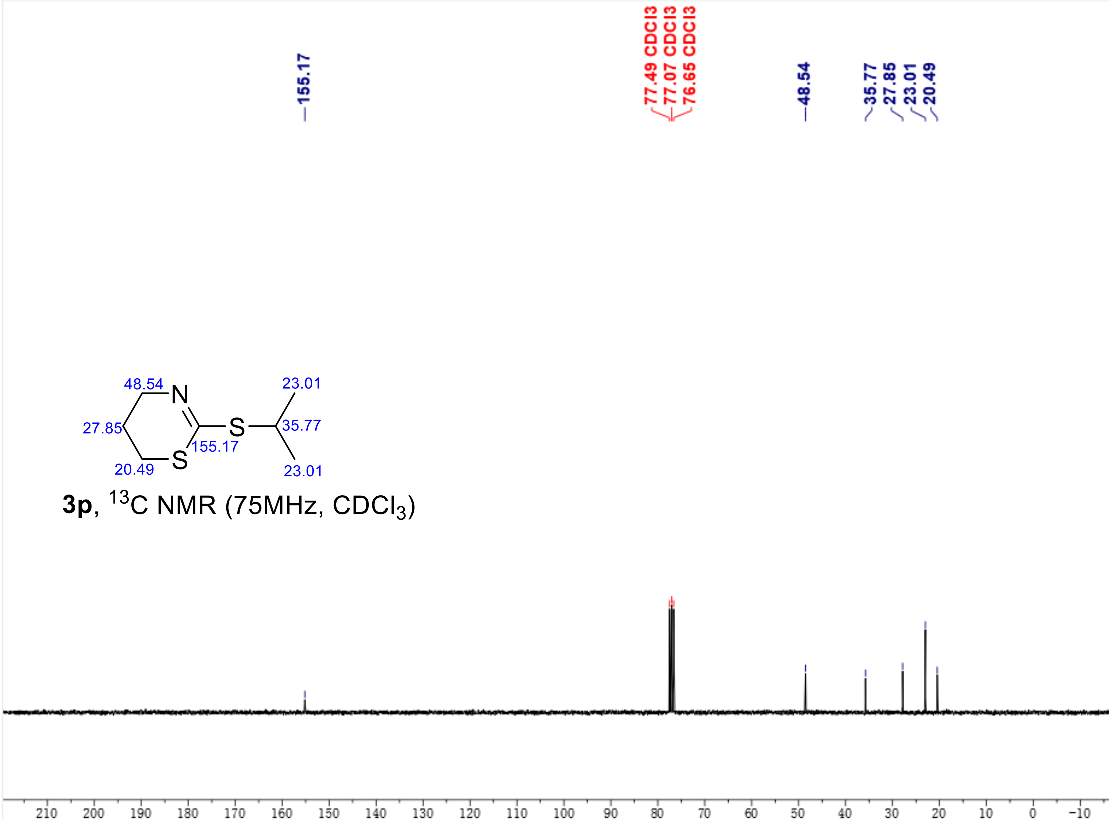

3p, ^{13}C NMR (75MHz, $CDCl_3$)

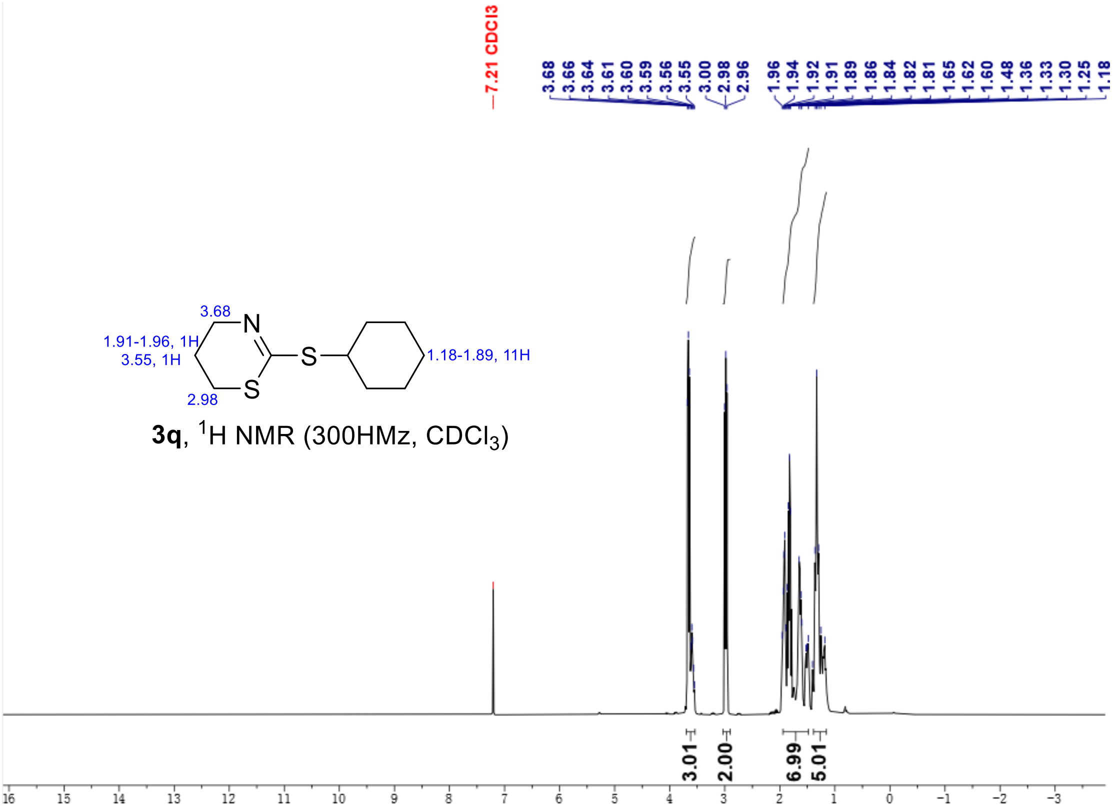

3q, ^{1}H NMR (300HMz, $CDCl_3$)

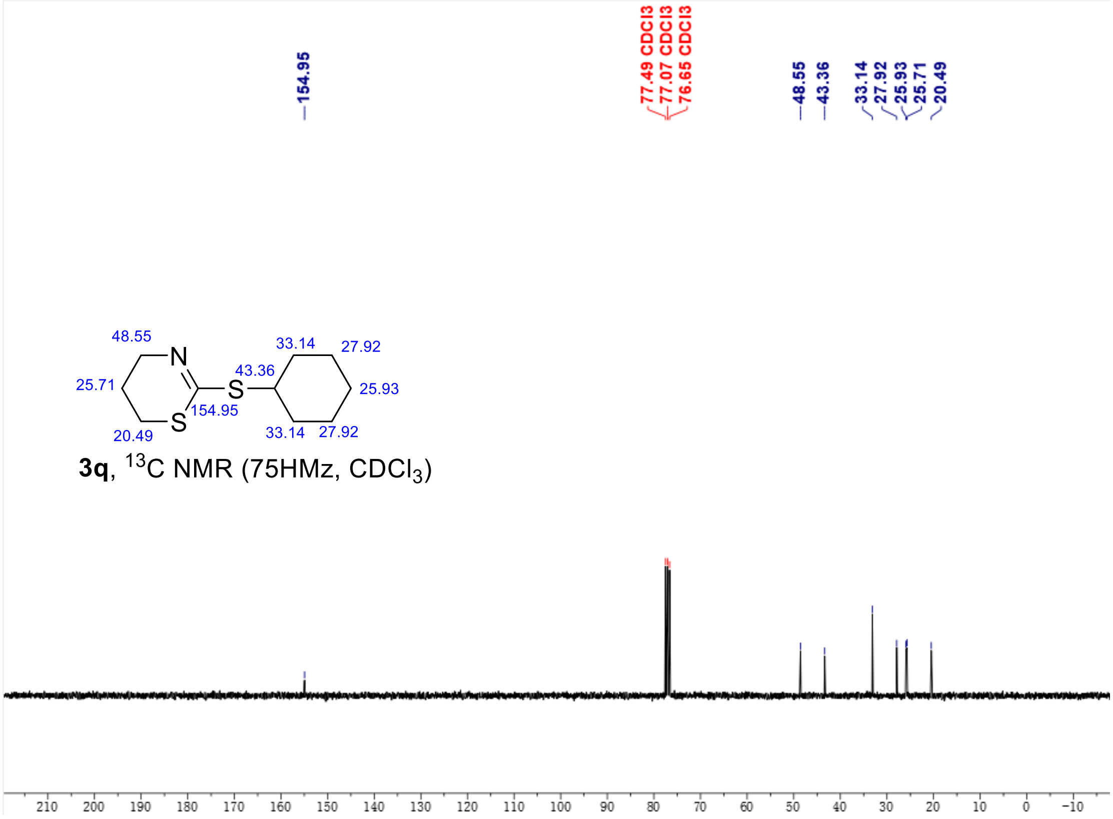

3q, ^{13}C NMR (75HMz, $CDCl_3$)

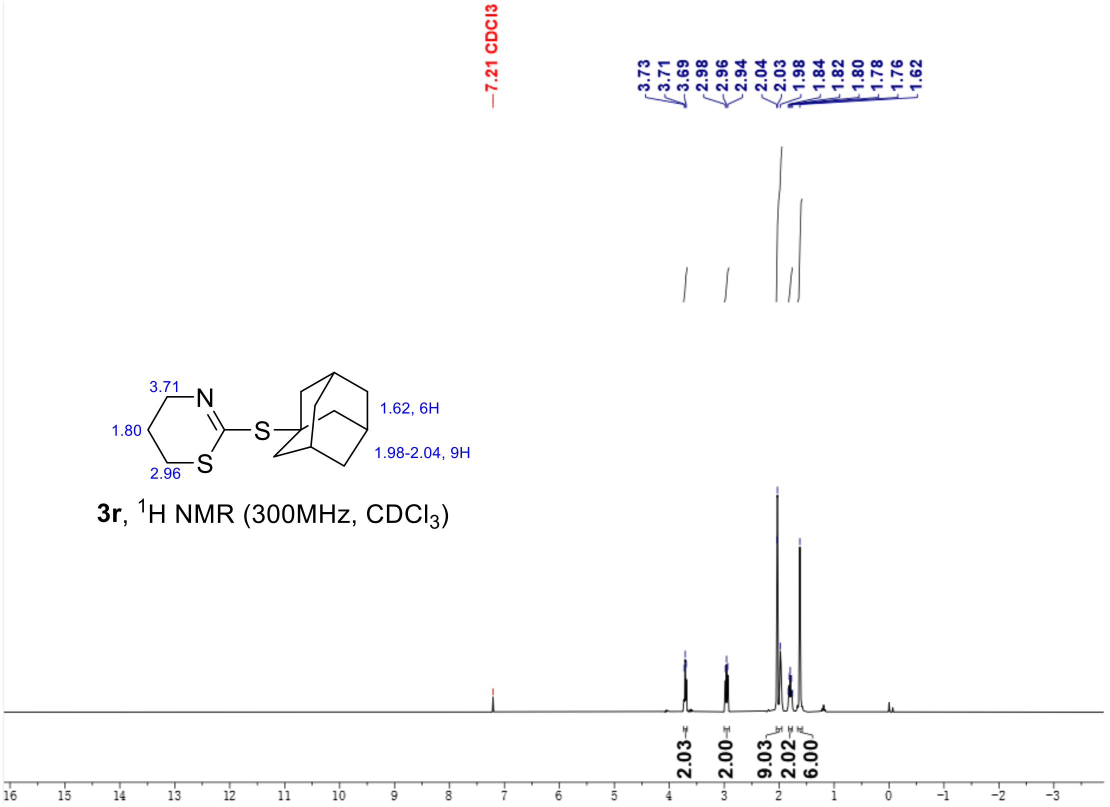

3r, ^{1}H NMR (300MHz, $CDCl_3$)

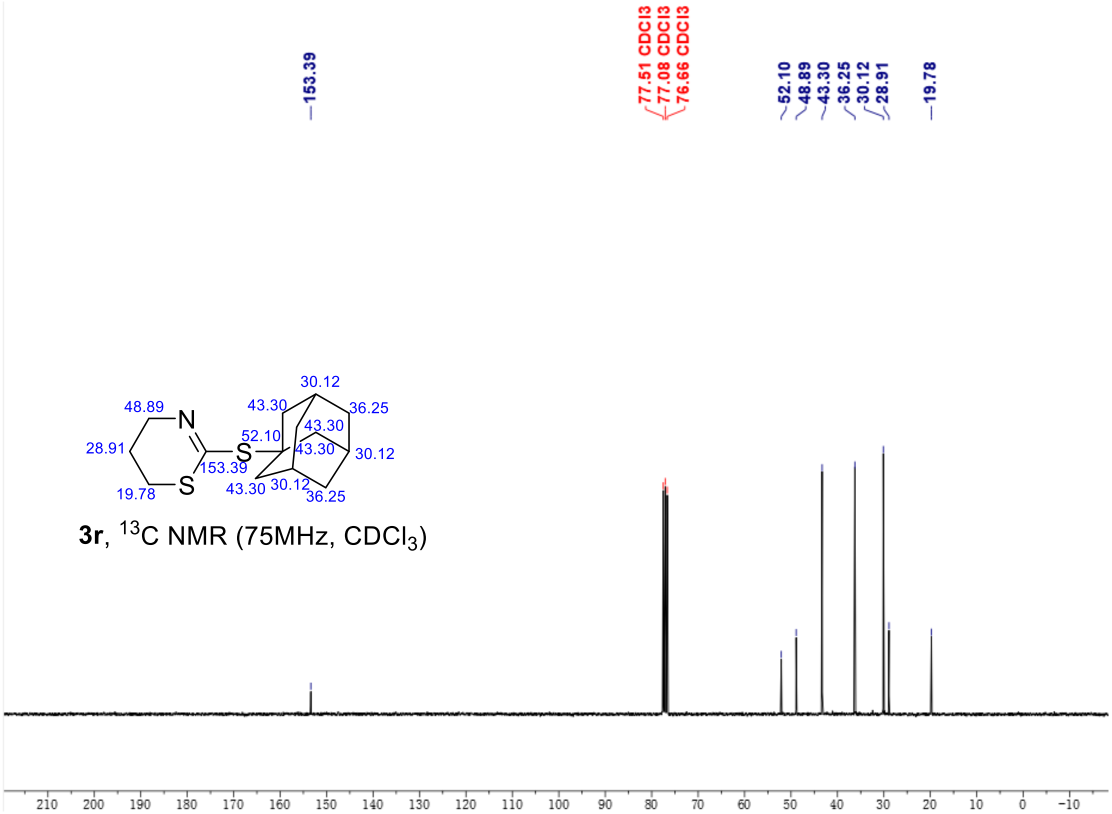

3r, ^{13}C NMR (75MHz, $CDCl_3$)

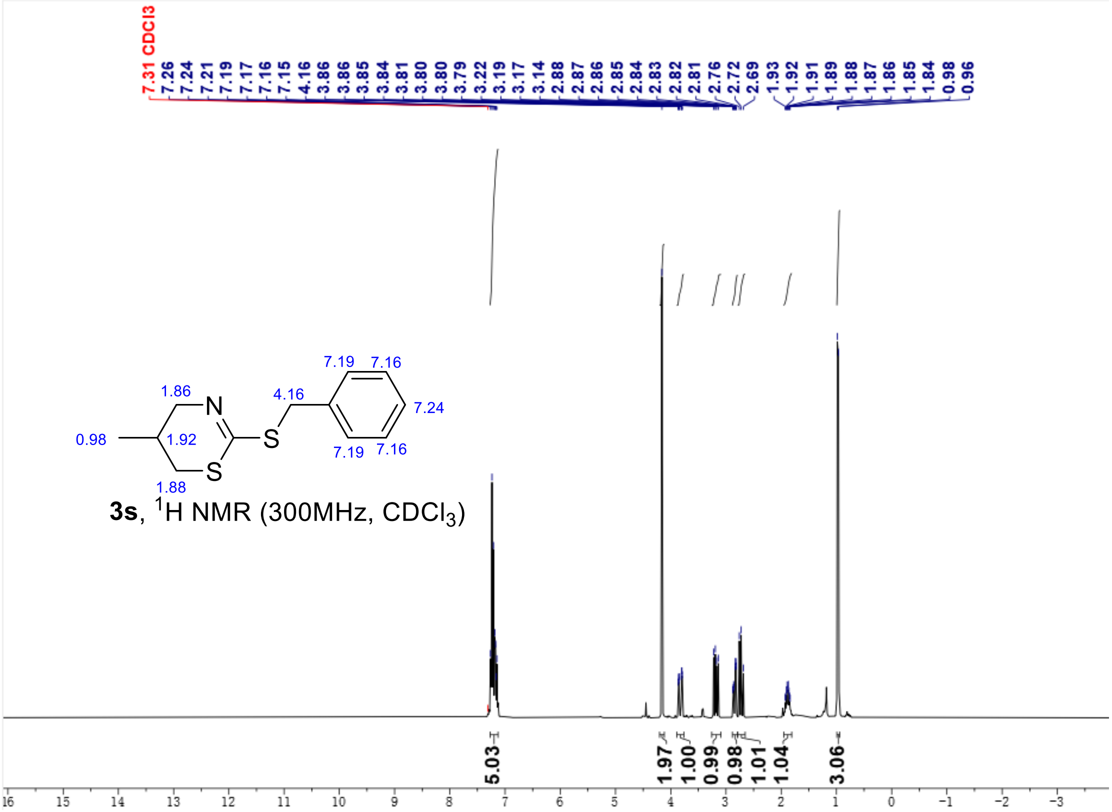

3s, ^{1}H NMR (300MHz, $CDCl_3$)

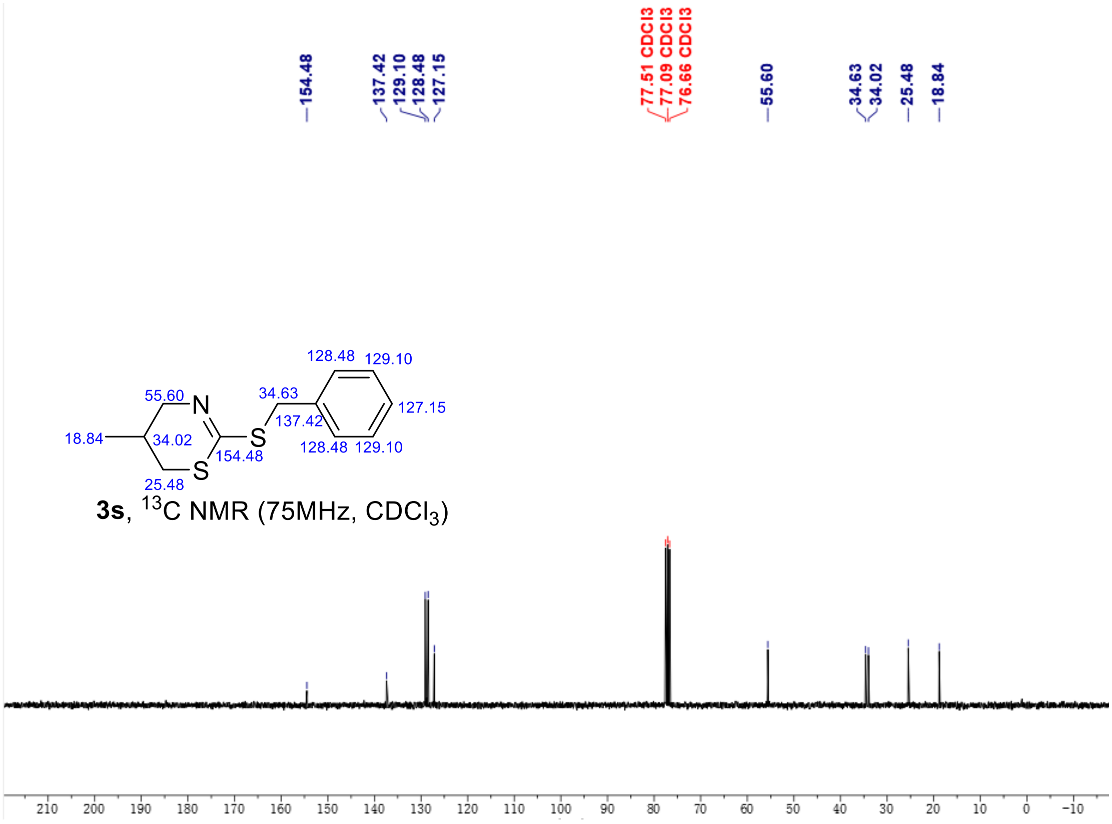

3s, ^{13}C NMR (75MHz, $CDCl_3$)

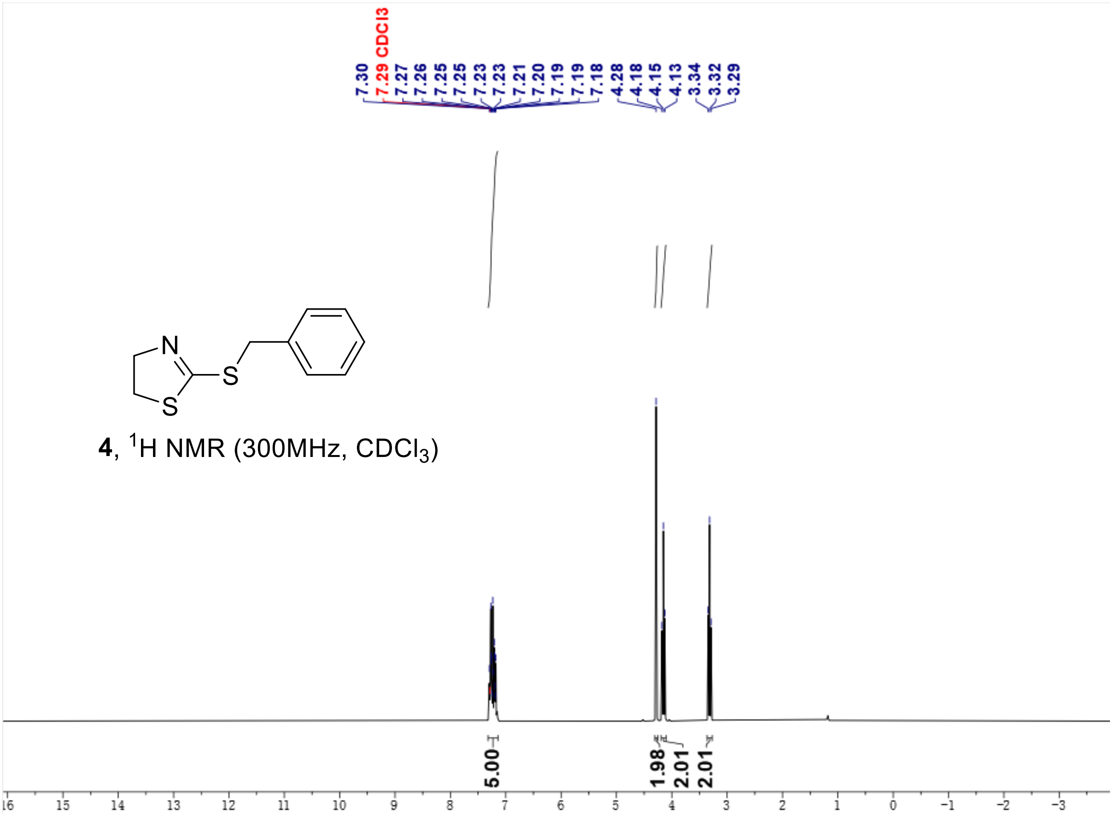

4, ^{1}H NMR (300MHz, $CDCl_3$)

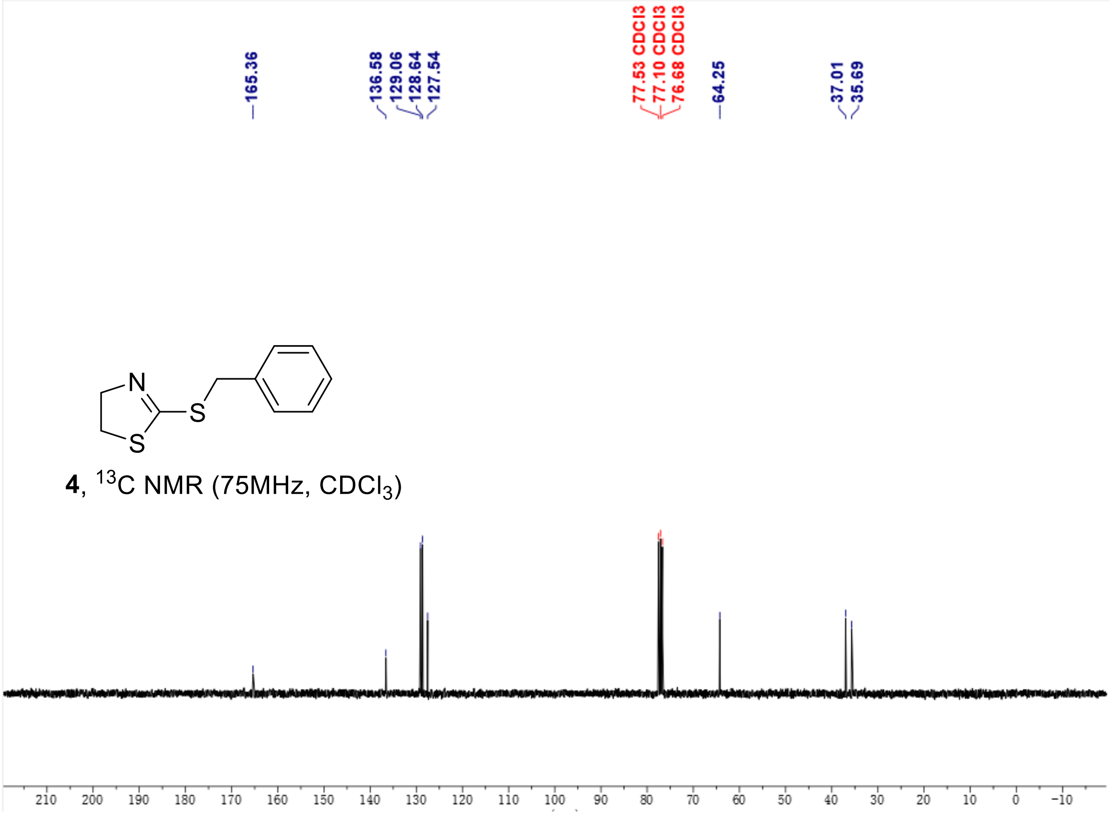

4, ^{13}C NMR (75MHz, $CDCl_3$)

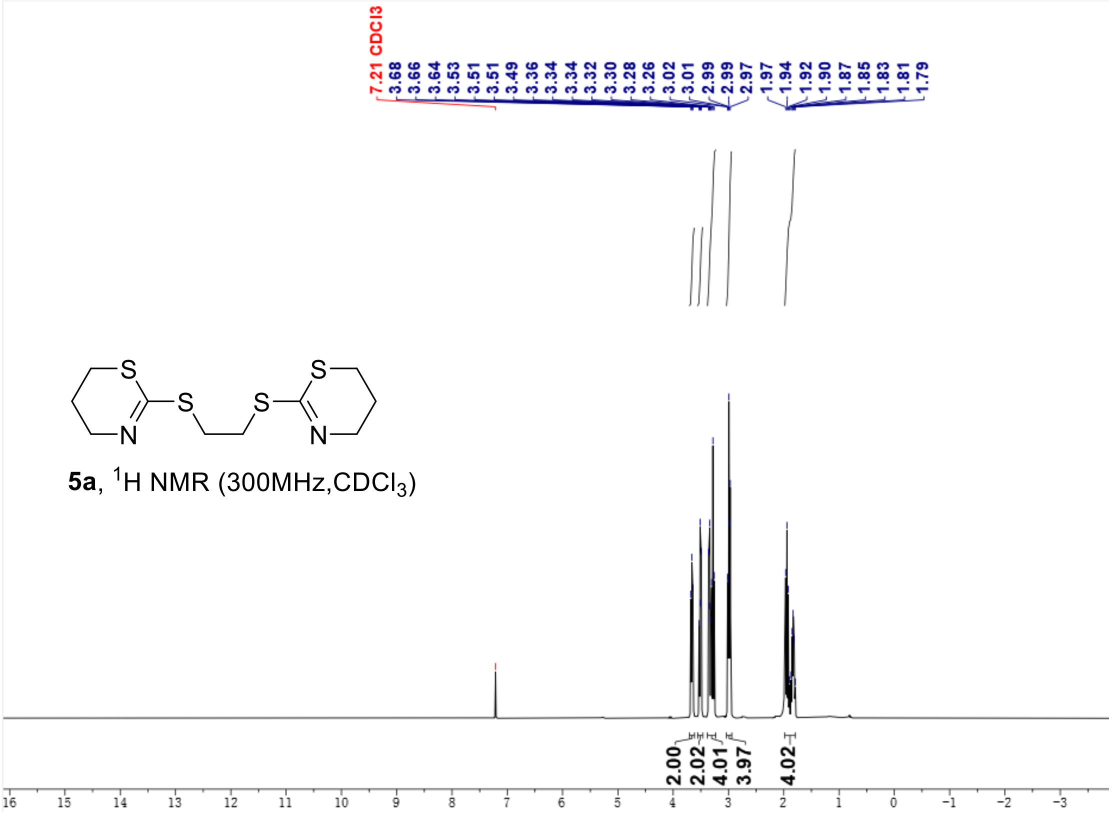

5a, ^{1}H NMR (300MHz,$CDCl_3$)

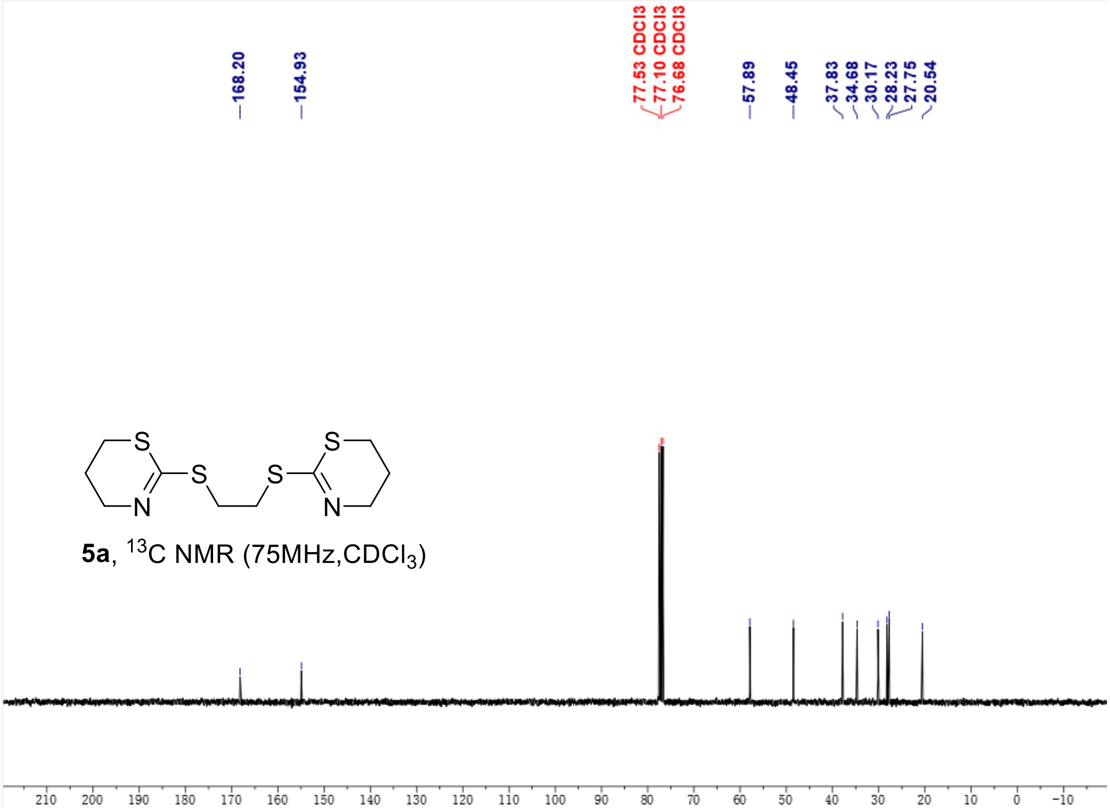

5a, ^{13}C NMR (75MHz,$CDCl_3$)

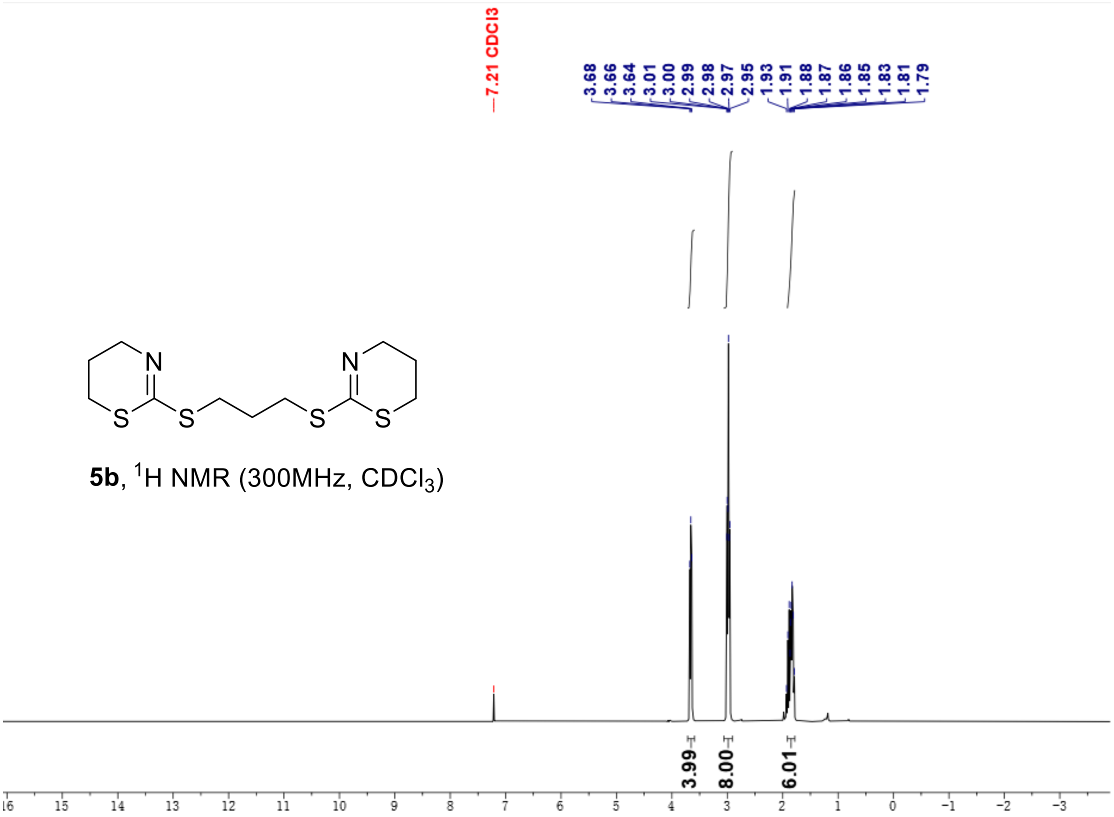

5b, ^{1}H NMR (300MHz, $CDCl_3$)

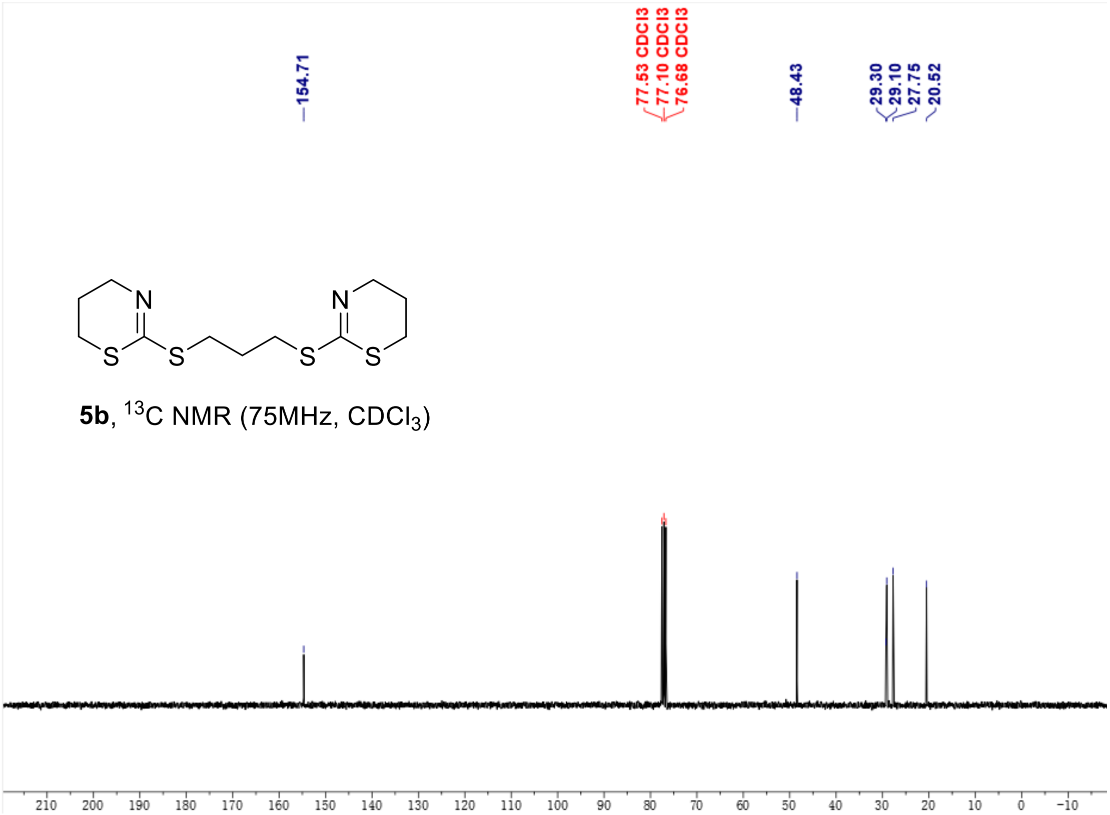

5b, ^{13}C NMR (75MHz, $CDCl_3$)

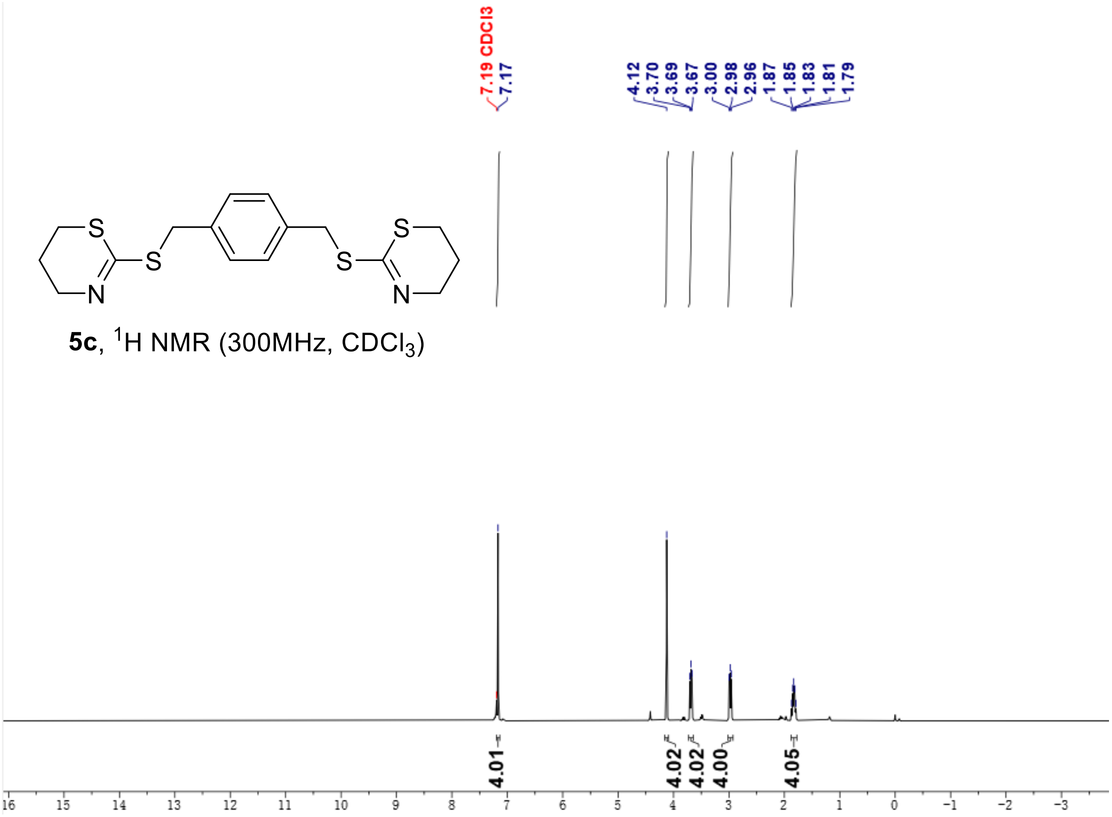

5c, ^{1}H NMR (300MHz, $CDCl_3$)

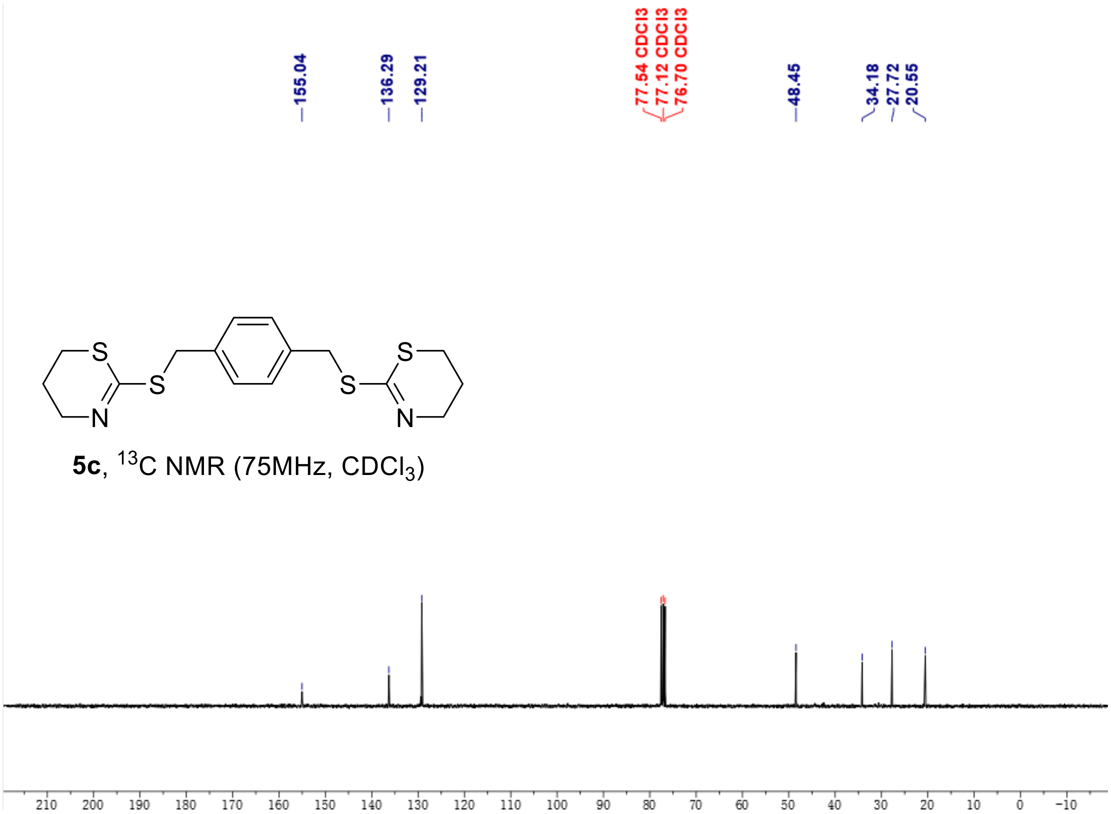

5c, ^{13}C NMR (75MHz, $CDCl_3$)

5. HRMS of products 3-5

3a

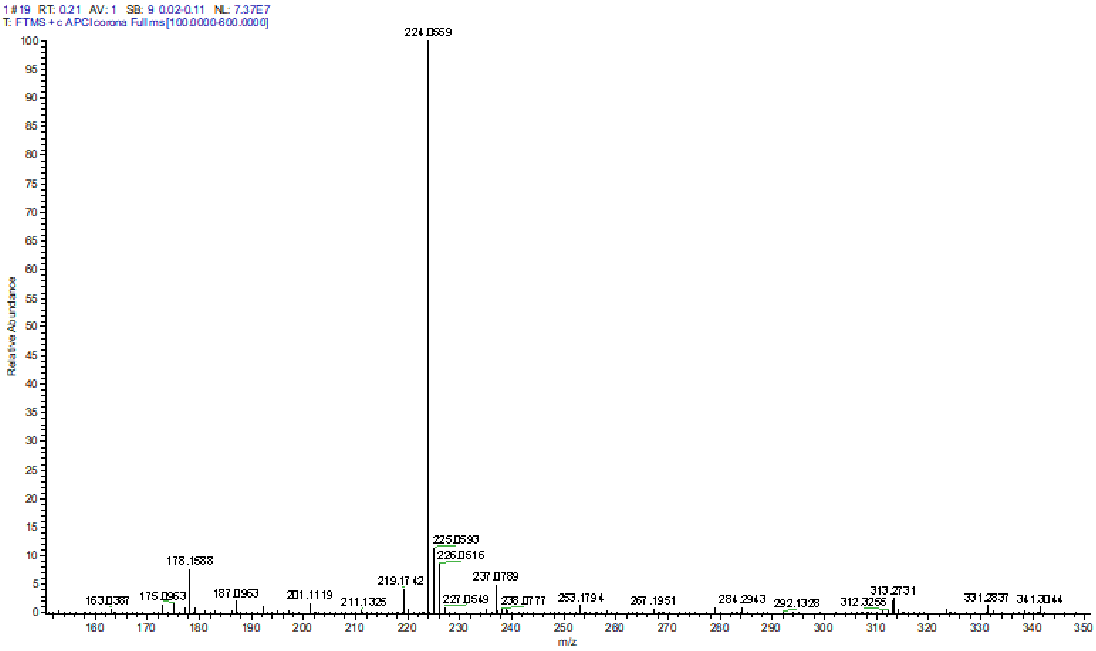

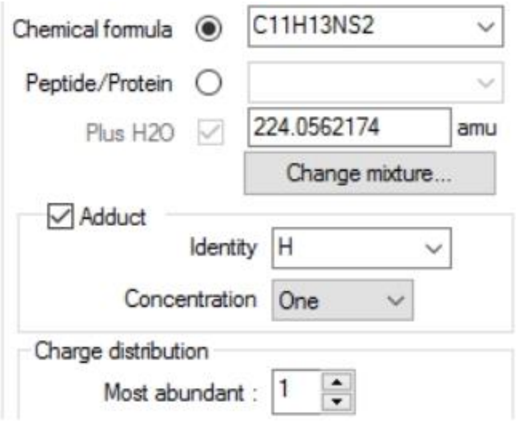

3b

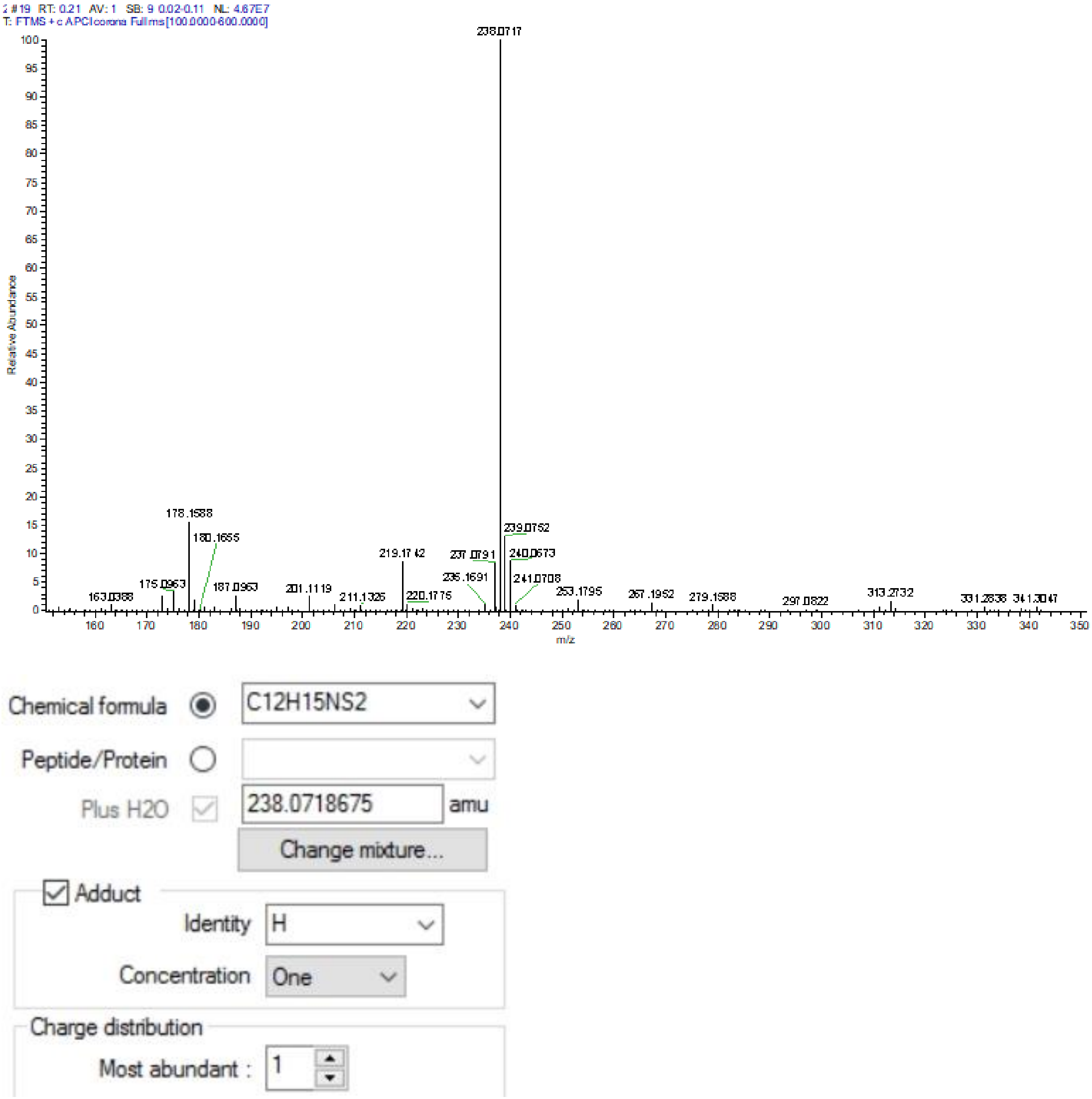
T: FTMS + c APCI corona Full ms [100.0000-600.0000]
238.0717
178.1588
180.1655
175.0963
163.0388
187.0963
201.1119
211.1326
219.1742
220.1775
236.1691
239.0752
240.0673
241.0708
253.1795
267.1952
279.1588
297.0822
313.2732
Relative Abundance
m/z
Chemical formula
C12H15NS2
Peptide/Protein
Plus H2O
238.0718675
amu
Change mixture...
Adduct
Identity
H
Concentration
One
Charge distribution
Most abundant :
1

3c

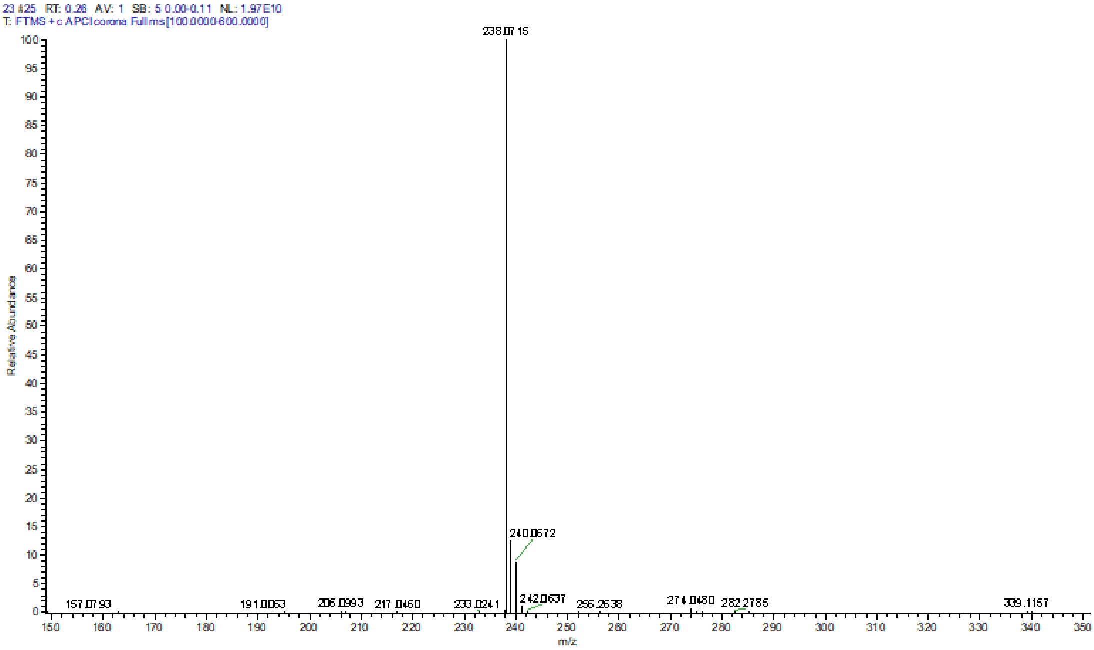

23 #25 RT: 0.26 AV: 1 SB: 5 0.00-0.11 NL: 1.97E10
T: FTMS + c APCI corona Full ms [100.0000-600.0000]
238.0715
240.0672
242.0637
157.0793
191.0063
205.0993
217.0450
233.0241
256.2538
274.0450
282.2785
339.1157
Relative Abundance
m/z

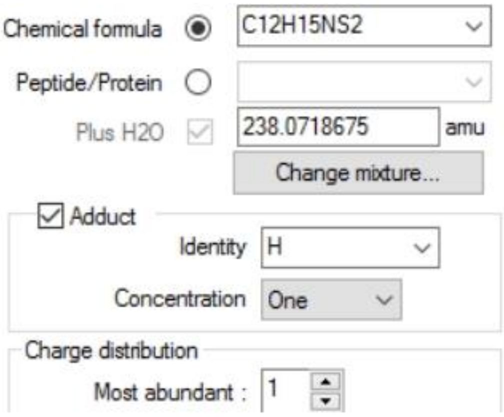

Chemical formula
C12H15NS2
Peptide/Protein
Plus H2O
238.0718675
amu
Change mixture...
Adduct
Identity
H
Concentration
One
Charge distribution
Most abundant :
1

3d

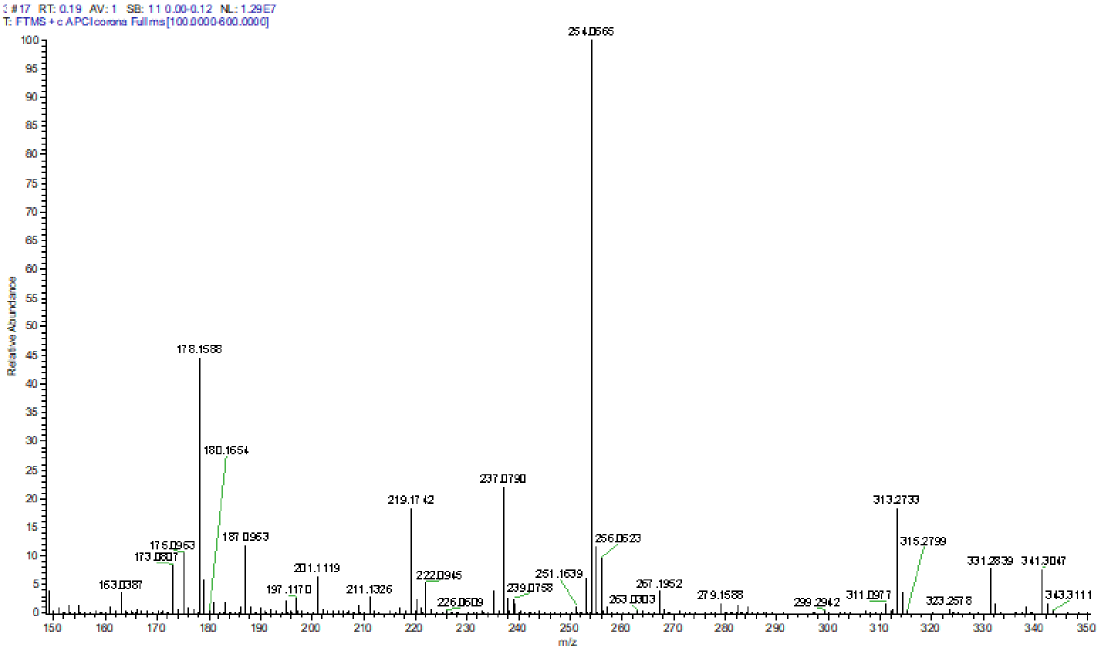
254.0565
178.1588
180.1654
237.0790
219.1742
313.2733
315.2799
187.0963
201.1119
331.2839
341.3047
Relative Abundance
m/z

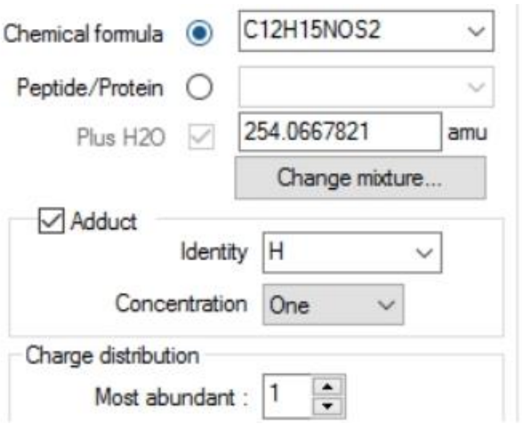
Chemical formula
C12H15NOS2
Peptide/Protein
Plus H2O
254.0667821
amu
Change mixture...
Adduct
Identity
H
Concentration
One
Charge distribution
Most abundant :
1

3e

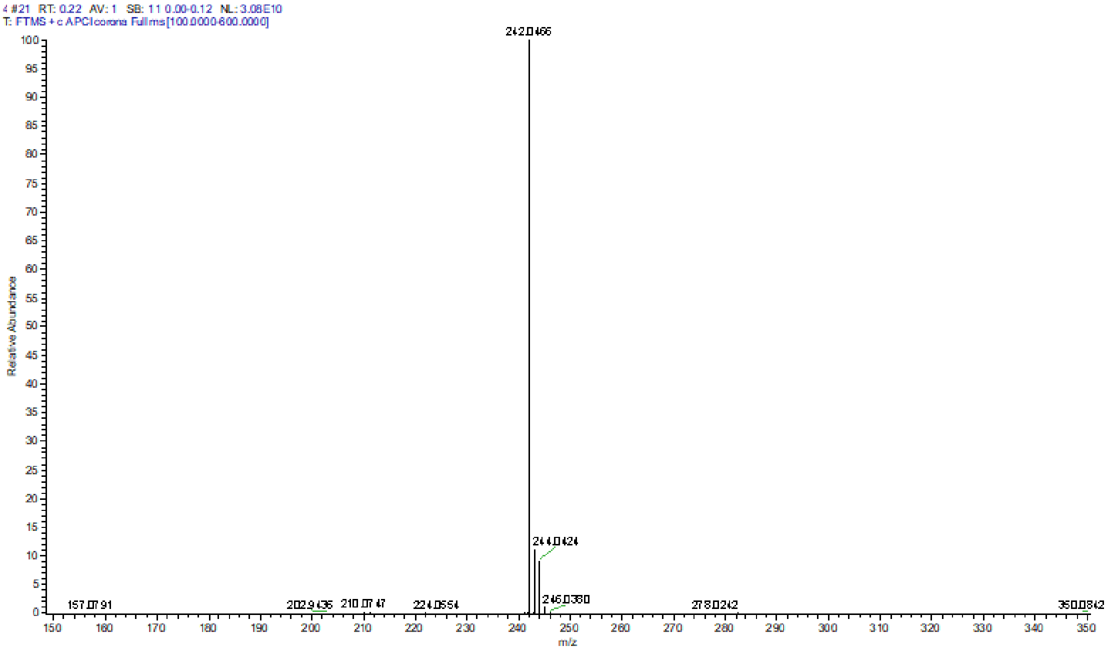
4 #21 RT: 0.22 AV: 1 SB: 11 0.00-0.12 NL: 3.08E10
T: FTMS + c APCI corona Full ms [100.0000-600.0000]
242.0466
244.0424
246.0380
157.0791
202.9436
210.0747
224.0554
278.0242
350.0842
Relative Abundance
m/z

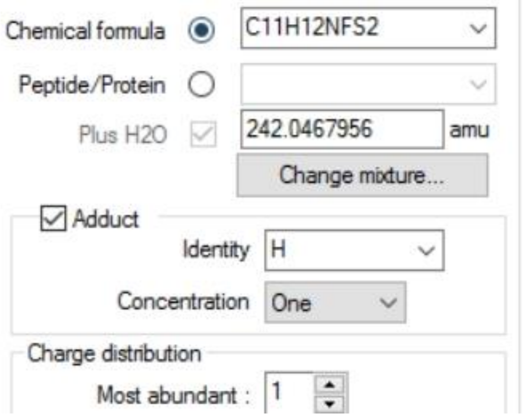
Chemical formula
C11H12NFS2
Peptide/Protein
Plus H2O
242.0467956
amu
Change mixture...
Adduct
Identity
H
Concentration
One
Charge distribution
Most abundant :
1

3f

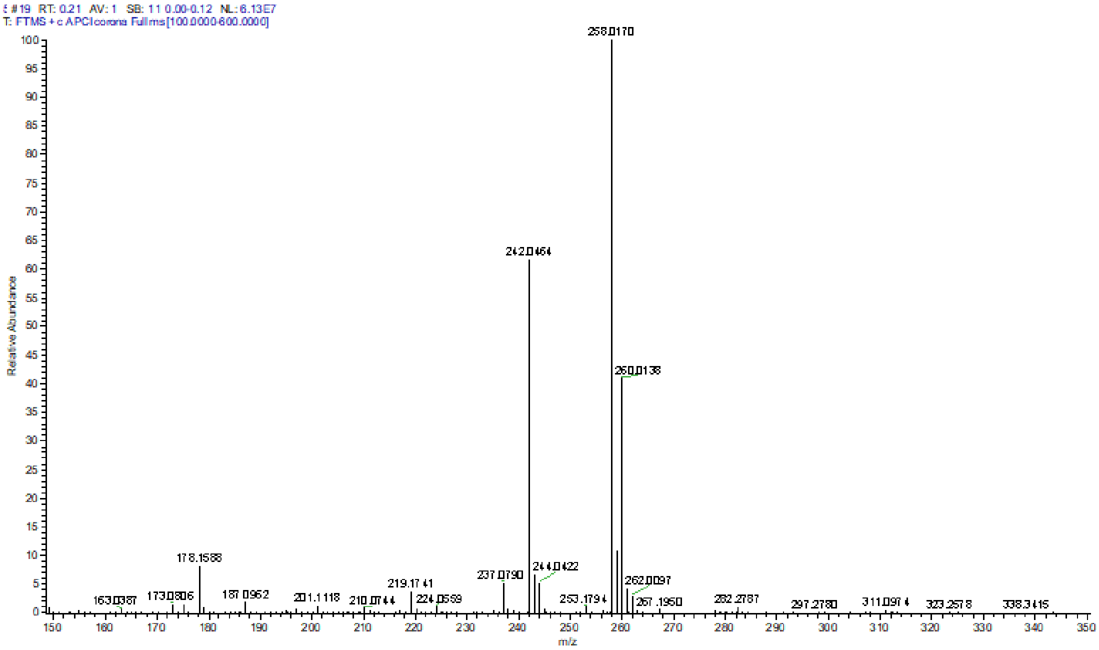

T: FTMS + c APCI corona Full ms [100.0000-600.0000]
Relative Abundance
m/z
258.0170
242.0464
260.0138
178.1588
244.0422
262.0097
237.0790
219.1741

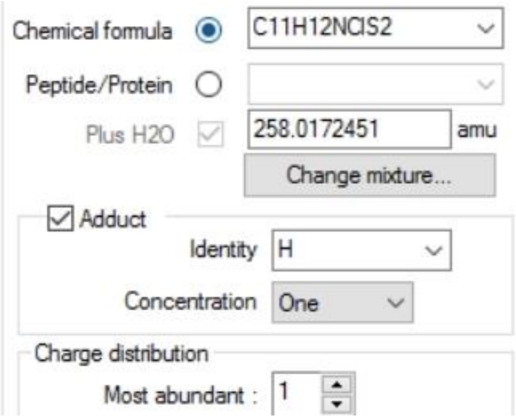

Chemical formula
C11H12NClS2
Peptide/Protein
Plus H2O
258.0172451
amu
Change mixture...
Adduct
Identity
H
Concentration
One
Charge distribution
Most abundant :
1

3g

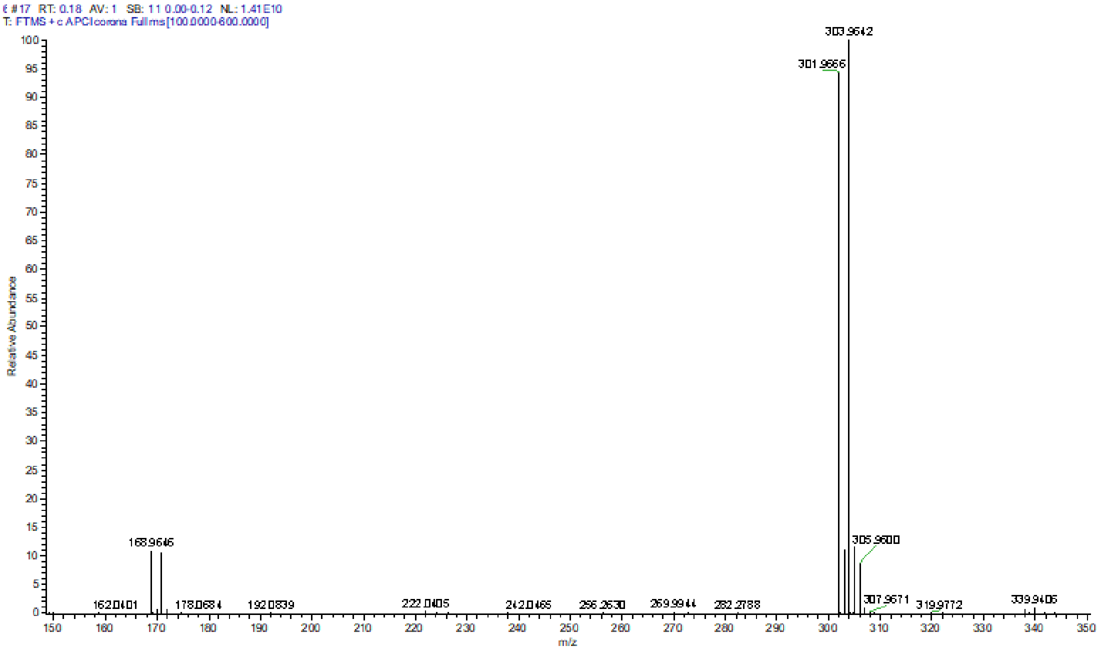

T: FTMS + c APCI corona Full ms [100.0000-600.0000]
303.9642
301.9666
168.9646
305.9600
307.9671
319.9772
339.9405
162.0401
178.0684
192.0839
222.0405
242.0465
256.2630
269.9944
282.2788
Relative Abundance
m/z

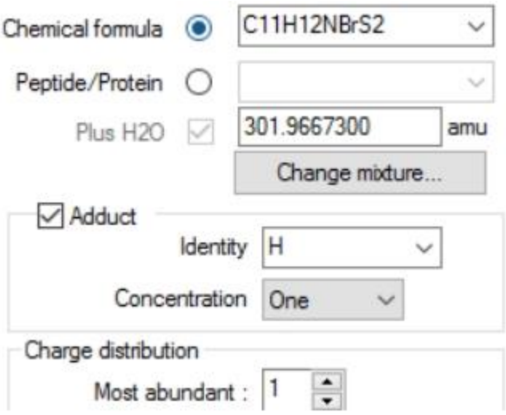

Chemical formula
C11H12NBrS2
Peptide/Protein
Plus H2O
301.9667300
amu
Change mixture...
Adduct
Identity
H
Concentration
One
Charge distribution
Most abundant :
1

3h

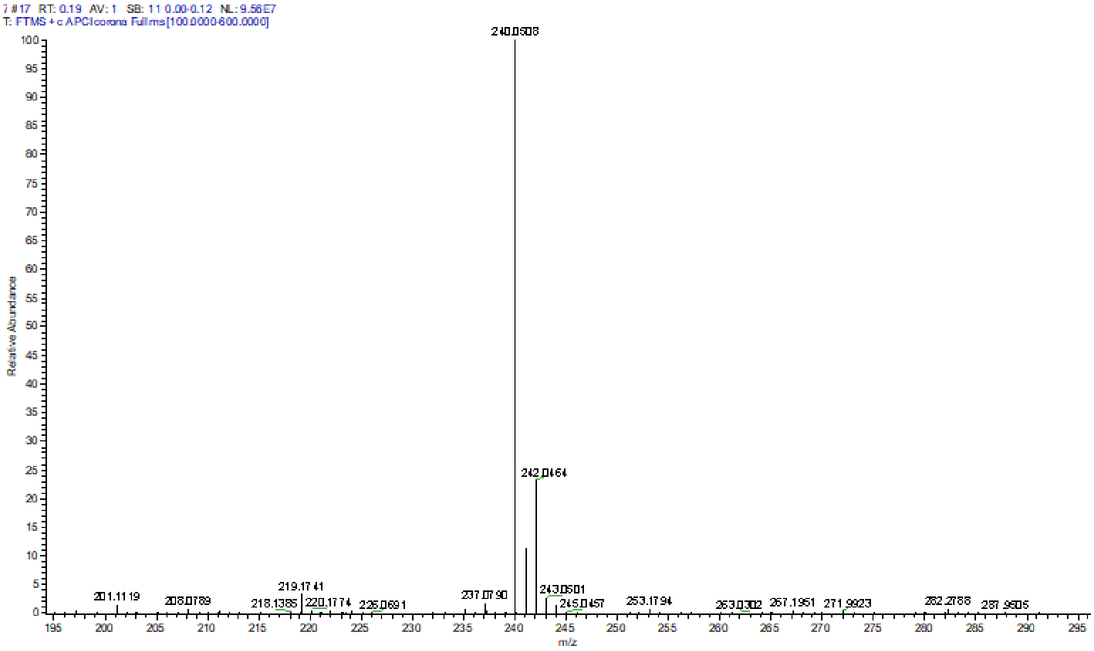

T: FTMS + c APCI corona Full ms [100.0000-600.0000]
240.0508
242.0464
Relative Abundance
m/z

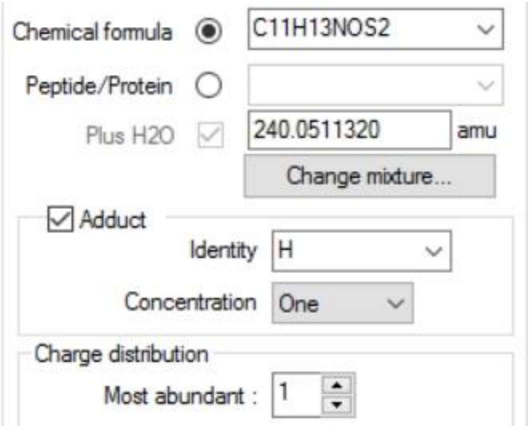

Chemical formula
C11H13NOS2
Peptide/Protein
Plus H2O
240.0511320
amu
Change mixture...
Adduct
Identity
H
Concentration
One
Charge distribution
Most abundant :
1

3i

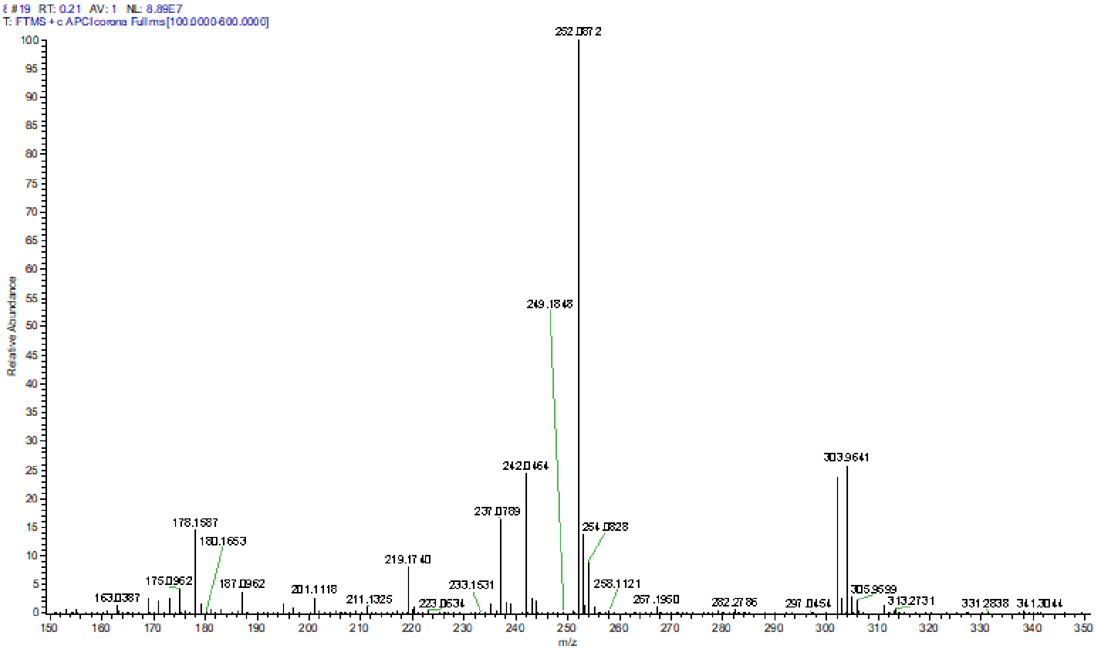

Relative Abundance
m/z
252.0872
249.1848
303.9641
242.0464
237.0789
254.0828
178.1587
180.1653
219.1740

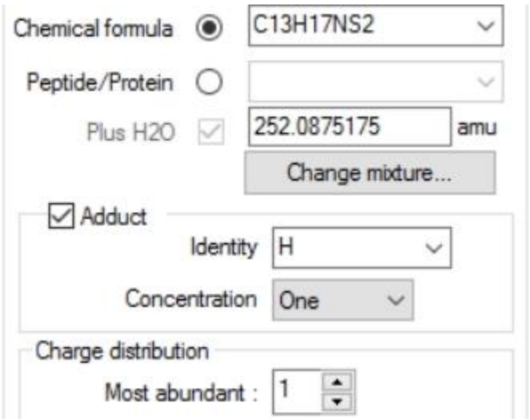

Chemical formula
C13H17NS2
Peptide/Protein
Plus H2O
252.0875175
amu
Change mixture...
Adduct
Identity
H
Concentration
One
Charge distribution
Most abundant :
1

3j

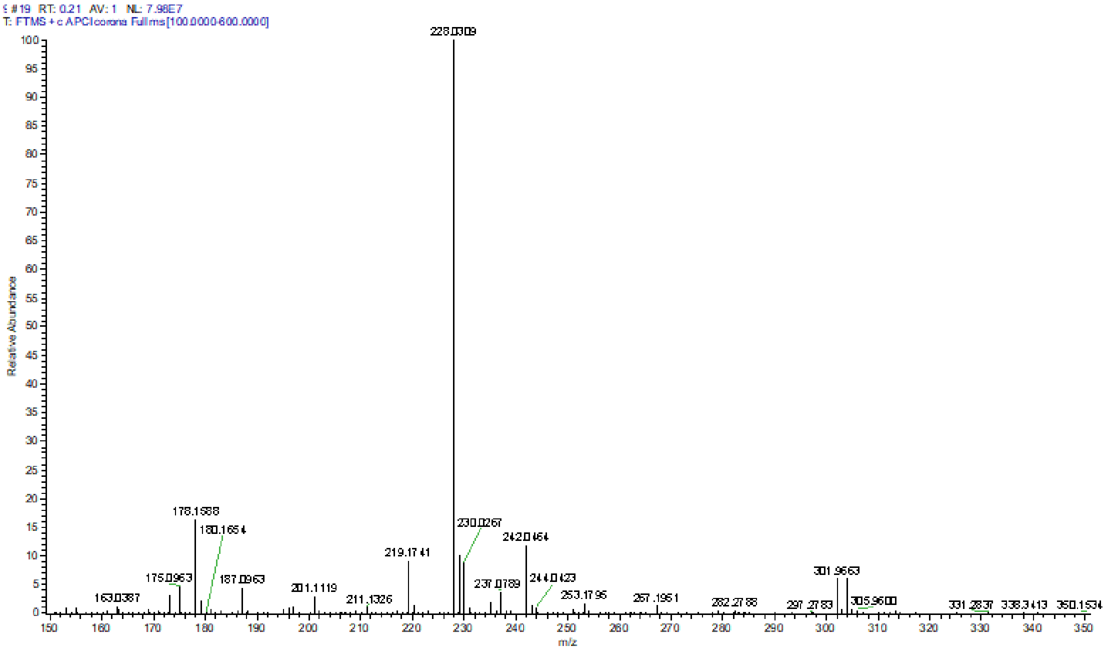
#19 RT: 0.21 AV: 1 NL: 7.98E7
T: FTMS + c APCI corona Full ms [100.0000-600.0000]
228.0309
178.1588
180.1654
175.0963
187.0963
163.0387
201.1119
211.1326
219.1741
230.0267
242.0464
237.0789
244.0423
253.1795
267.1951
282.2788
297.2783
301.9663
305.9600
331.2837
338.3413
350.1534
Relative Abundance
m/z

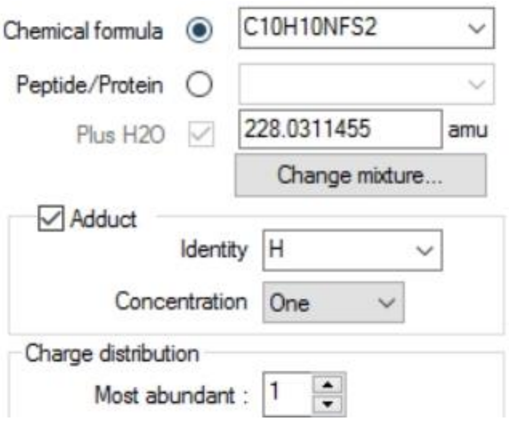
Chemical formula
C10H10NFS2
Peptide/Protein
Plus H2O
228.0311455
amu
Change mixture...
Adduct
Identity
H
Concentration
One
Charge distribution
Most abundant :
1

3k

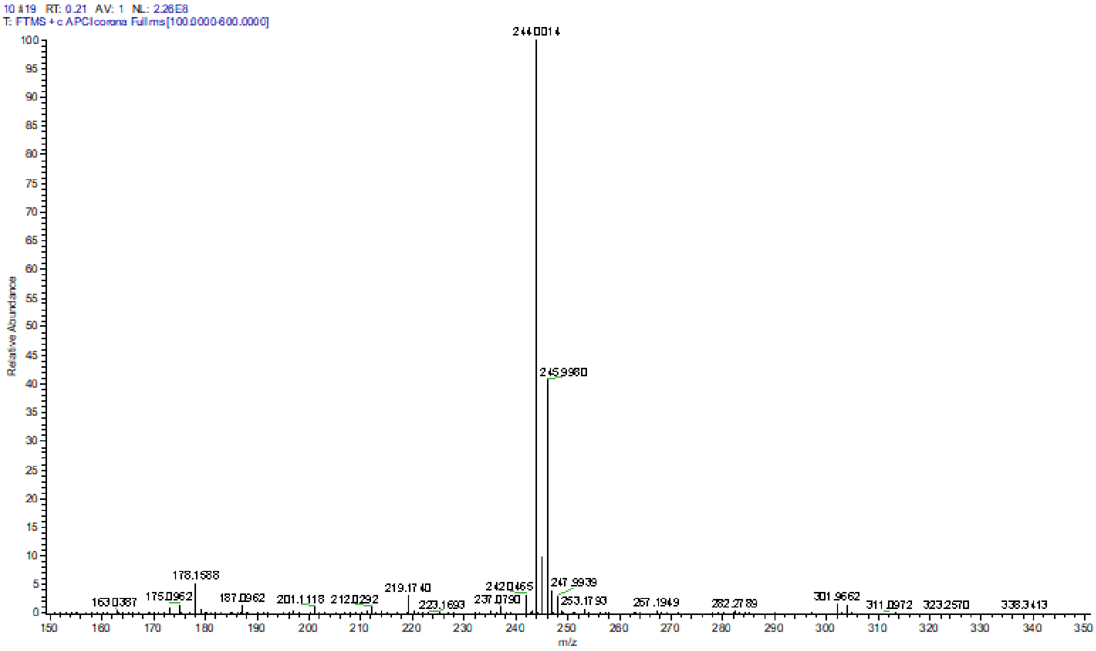

T: FTMS + c APCI corona Full ms [100.0000-600.0000]
244.0014
245.9980
178.1588
Relative Abundance
m/z

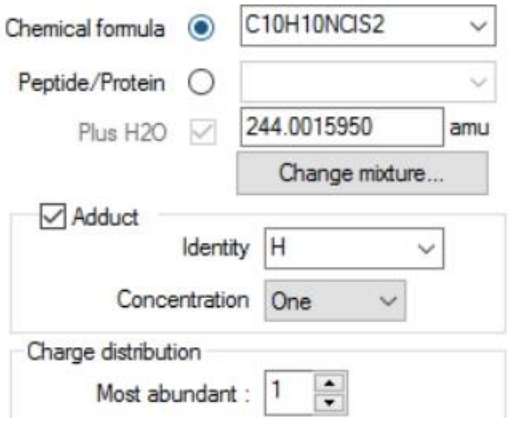

Chemical formula
C10H10NClS2
Peptide/Protein
Plus H2O
244.0015950
amu
Change mixture...
Adduct
Identity
H
Concentration
One
Charge distribution
Most abundant :
1

3l

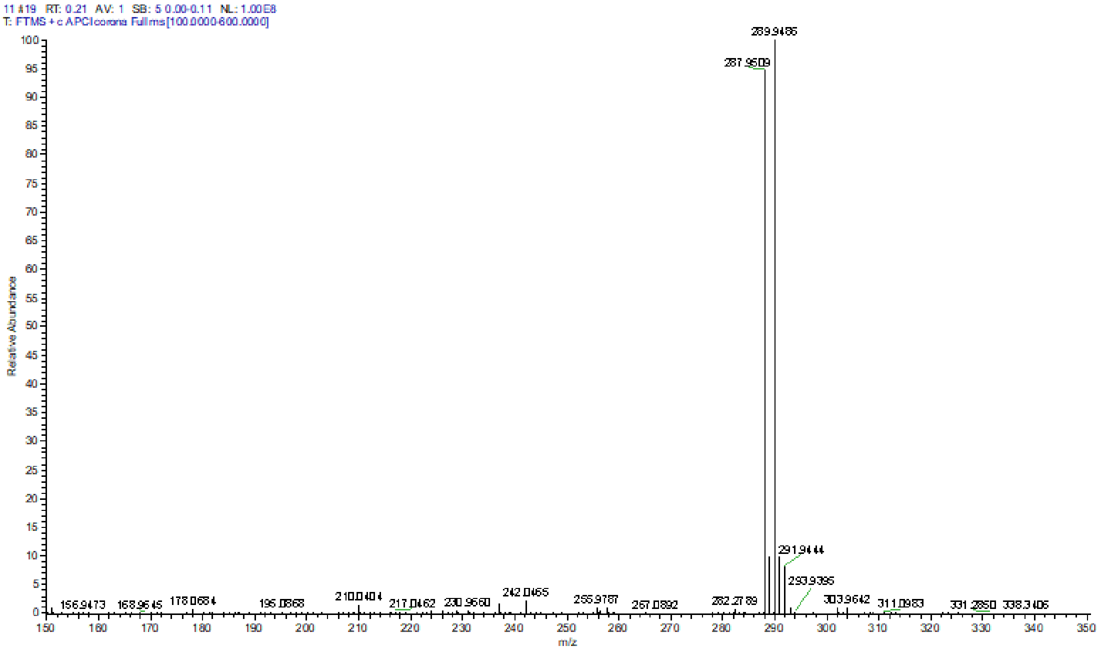

11 #19 RT: 0.21 AV: 1 SB: 5 0.00-0.11 NL: 1.00E8
T: FTMS + c APCI corona Full ms [100.0000-600.0000]
289.9486
287.9509
291.9444
293.9395
Relative Abundance
m/z

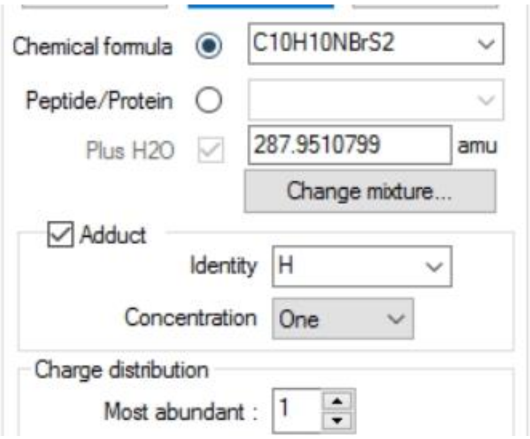

Chemical formula
C10H10NBrS2
Peptide/Protein
Plus H2O
287.9510799
amu
Change mixture...
Adduct
Identity
H
Concentration
One
Charge distribution
Most abundant :
1

3m

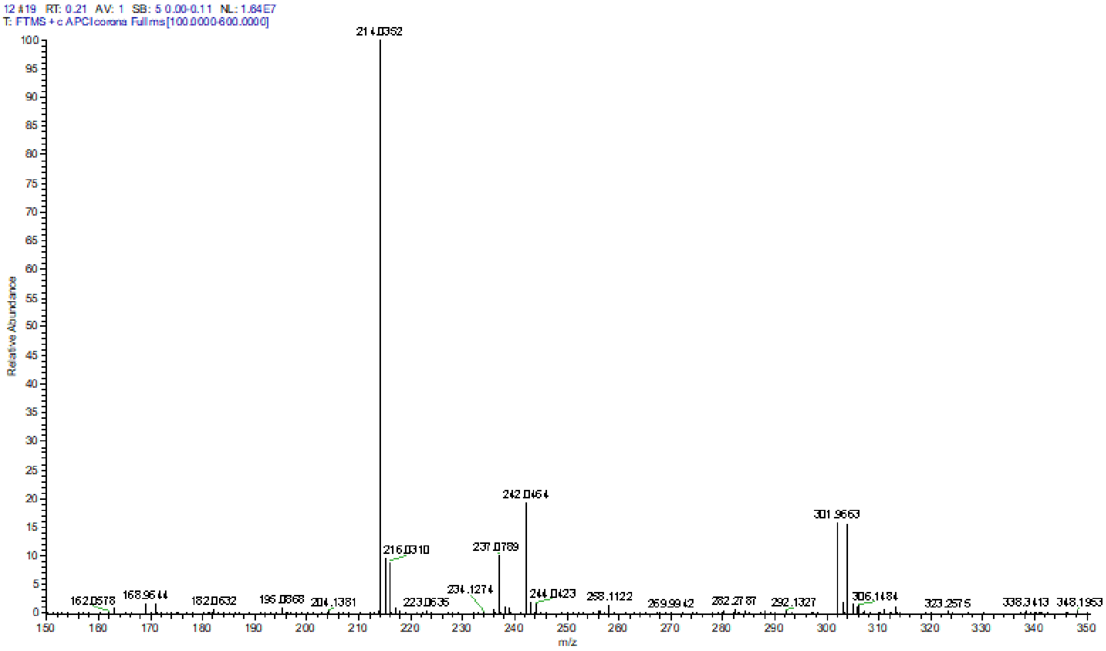

T: FTMS + c APCI corona Full ms [100.0000-600.0000]
Relative Abundance
m/z
214.0352
242.0454
301.9563
216.0310
237.0789

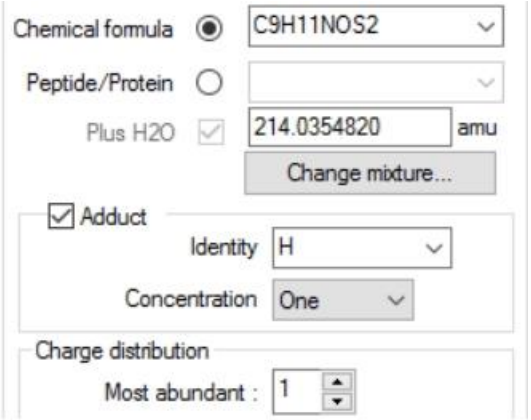

Chemical formula
C9H11NOS2
Peptide/Protein
Plus H2O
214.0354820
amu
Change mixture...
Adduct
Identity
H
Concentration
One
Charge distribution
Most abundant :
1

3n

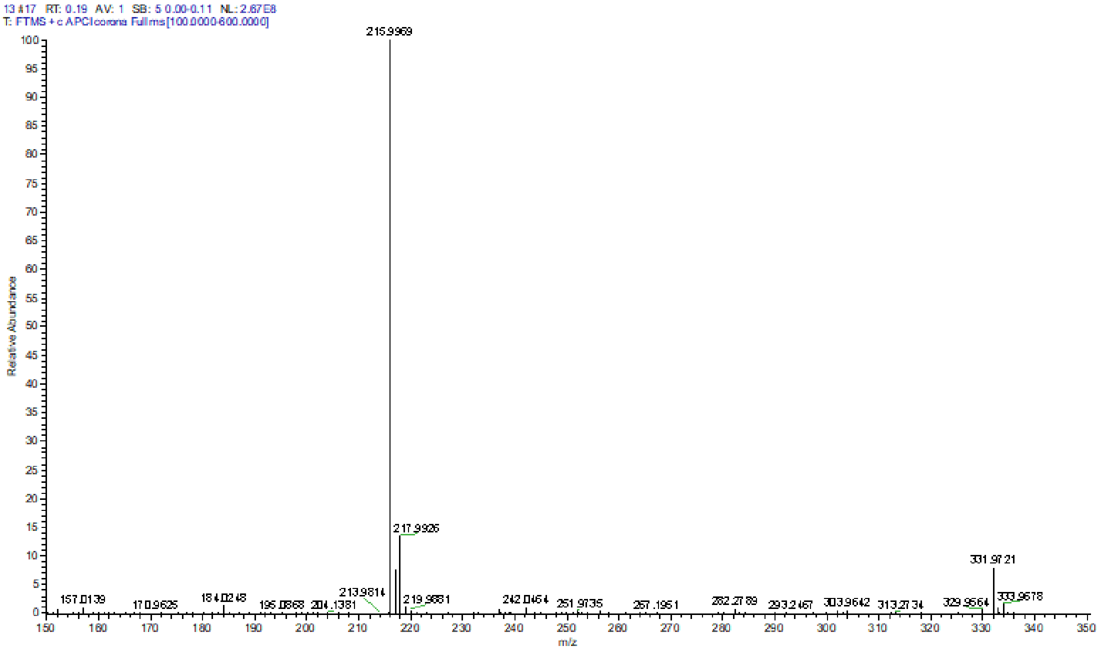

T: FTMS + c APCI corona Full ms [100.0000-600.0000]
215.9969
217.9926
331.9721
Relative Abundance
m/z

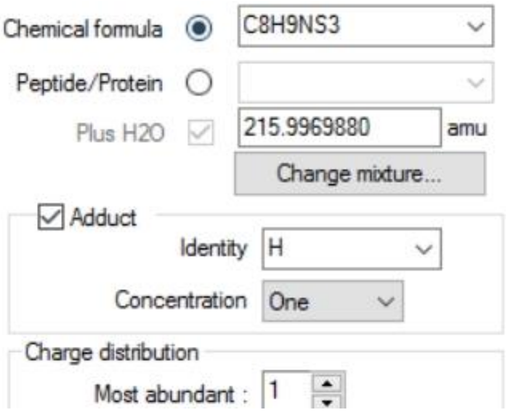

Chemical formula
C8H9NS3
Peptide/Protein
Plus H2O
215.9969880
amu
Change mixture...
Adduct
Identity
H
Concentration
One
Charge distribution
Most abundant :
1

3o

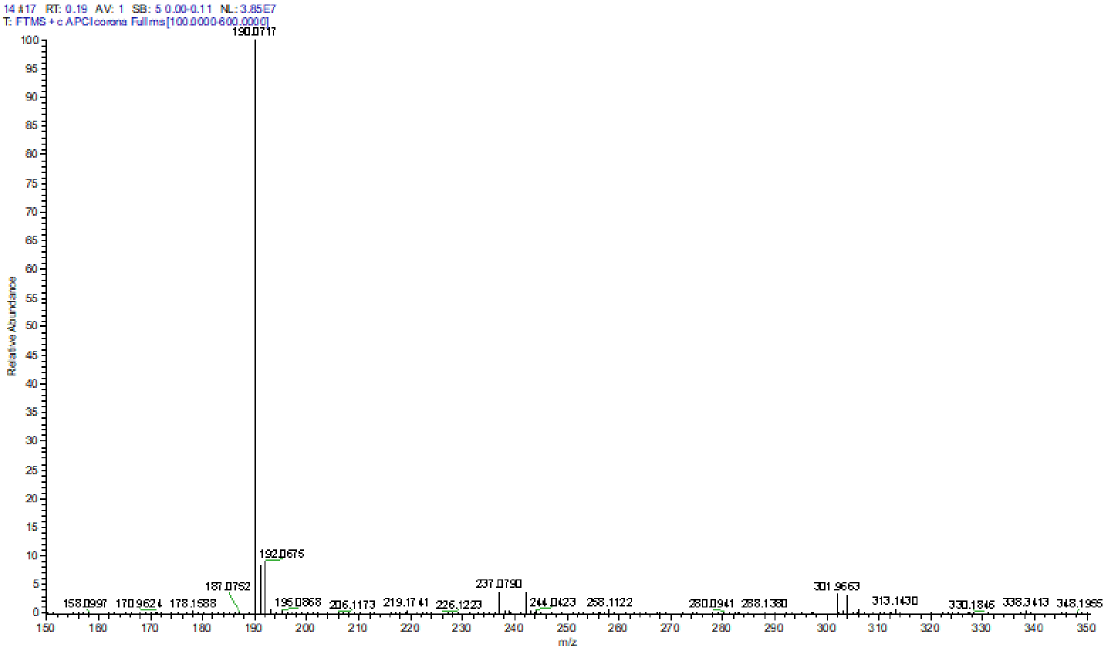

14 #17 RT: 0.19 AV: 1 SB: 5 0.00-0.11 NL: 3.85E7
T: FTMS + c APCI corona Full ms [100.0000-600.0000]
190.0717
192.0675
187.0752
237.0790
301.9663
Relative Abundance
m/z

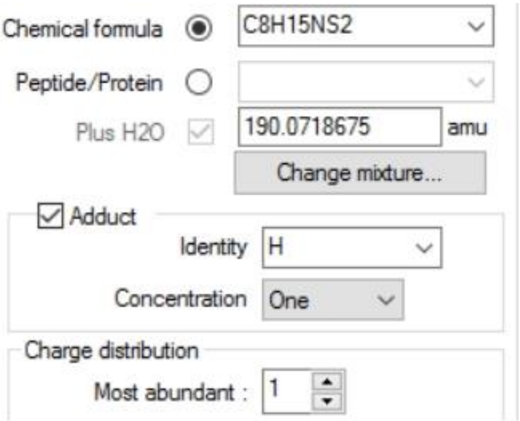

Chemical formula
C8H15NS2
Peptide/Protein
Plus H2O
190.0718675
amu
Change mixture...
Adduct
Identity
H
Concentration
One
Charge distribution
Most abundant :
1

3p

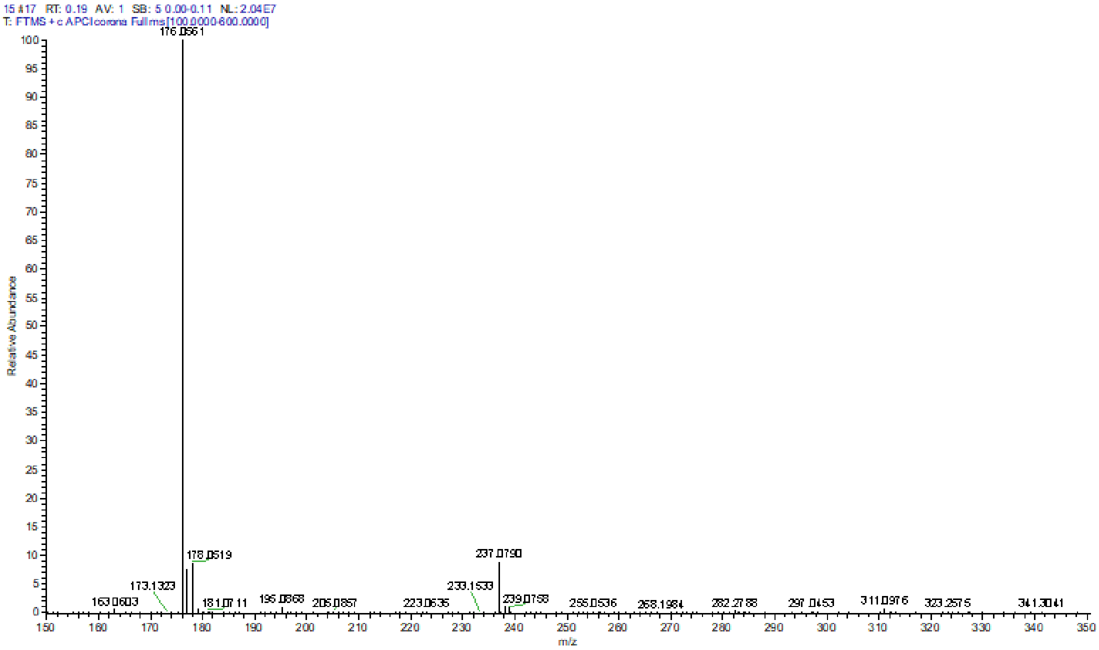
176.0561
178.0519
237.0790
173.1323
233.1533
Relative Abundance
m/z

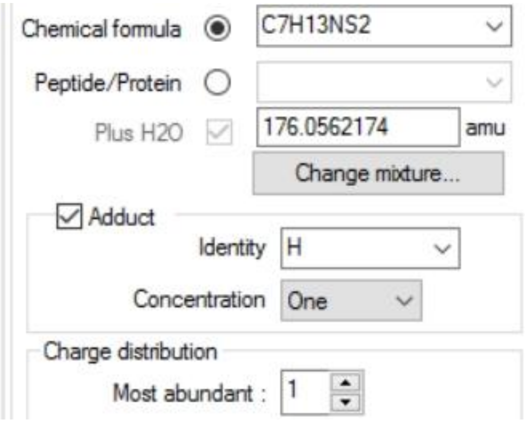
Chemical formula
C7H13NS2
Peptide/Protein
Plus H2O
176.0562174
amu
Change mixture...
Adduct
Identity
H
Concentration
One
Charge distribution
Most abundant :
1

3q

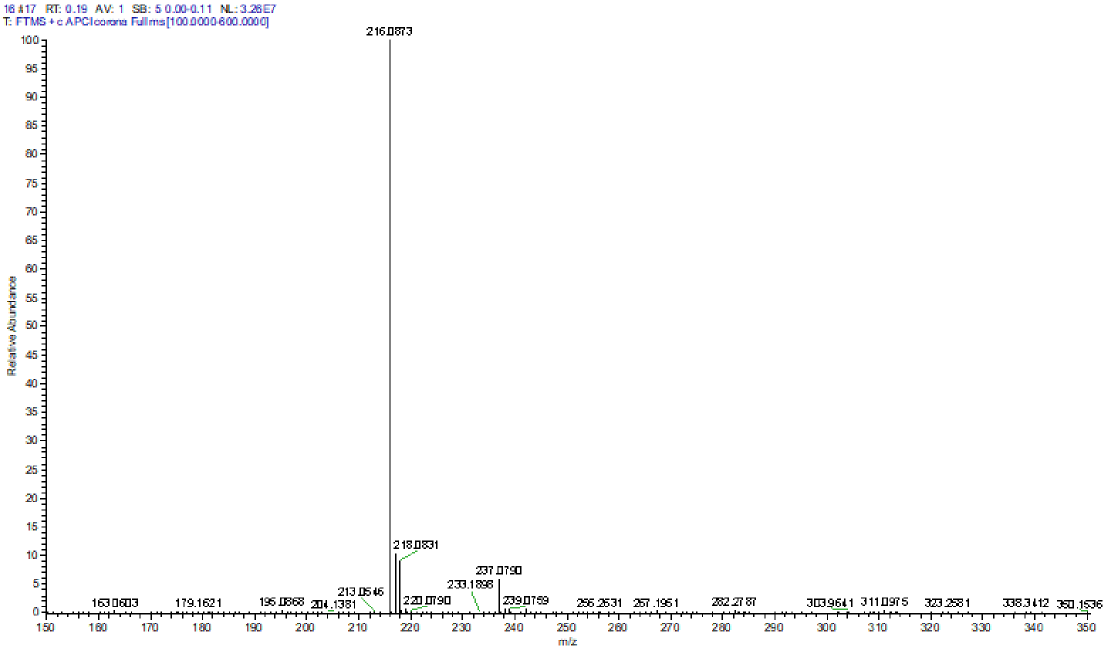
16 #17 RT: 0.19 AV: 1 SB: 5 0.00-0.11 NL: 3.26E7
T: FTMS + c APCI corona Full ms [100.0000-600.0000]
216.0873
218.0831
237.0790
233.1898
213.0546
220.0790
239.0759
163.0603
179.1621
195.0868
204.1381
256.2631
267.1951
282.2787
303.9641
311.0975
323.2581
338.3412
350.1536
Relative Abundance
m/z

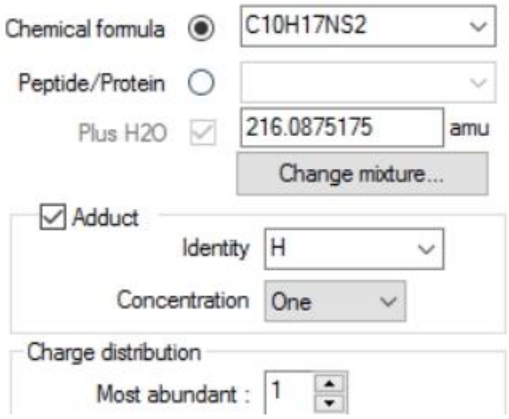
Chemical formula
C10H17NS2
Peptide/Protein
Plus H2O
216.0875175
amu
Change mixture...
Adduct
Identity
H
Concentration
One
Charge distribution
Most abundant :
1

3r

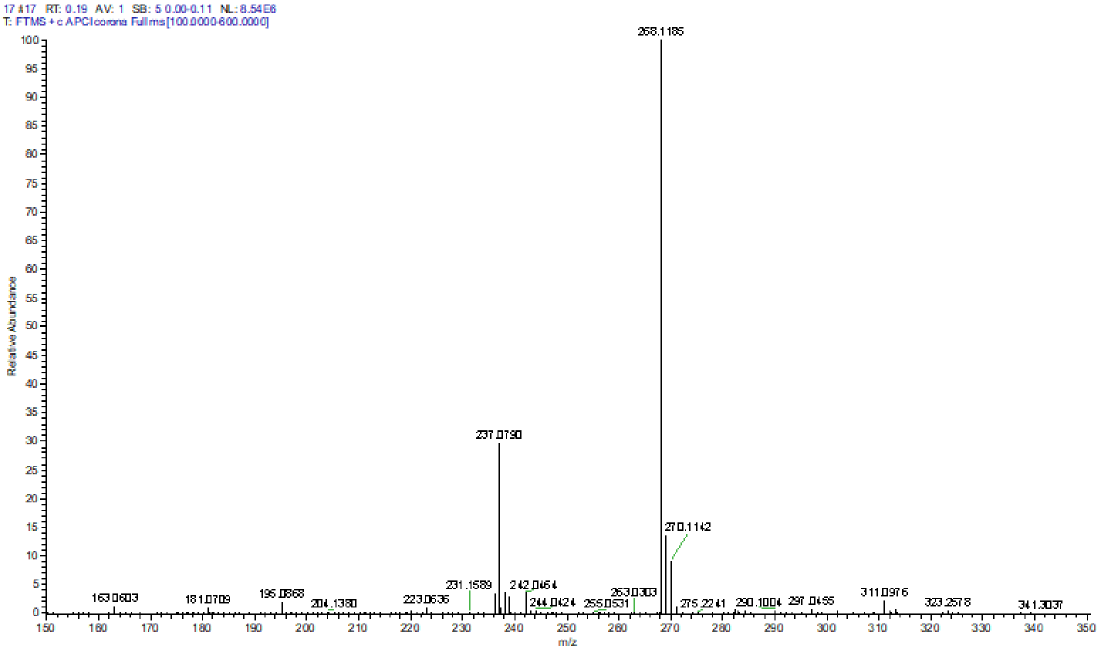
17 #17 RT: 0.19 AV: 1 SB: 5 0.00-0.11 NL: 8.54E6
T: FTMS + c APCI corona Full ms [100.0000-600.0000]
268.1185
237.0790
270.1142
231.1589
242.0454
244.0424
255.0531
263.0303
163.0603
181.0709
195.0868
204.1380
223.0636
275.2241
290.1004
297.0455
311.0976
323.2578
341.3037
Relative Abundance
m/z

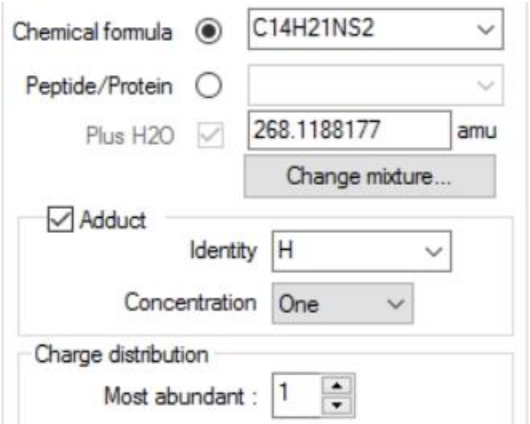
Chemical formula
C14H21NS2
Peptide/Protein
Plus H2O
268.1188177
amu
Change mixture...
Adduct
Identity
H
Concentration
One
Charge distribution
Most abundant :
1

3s

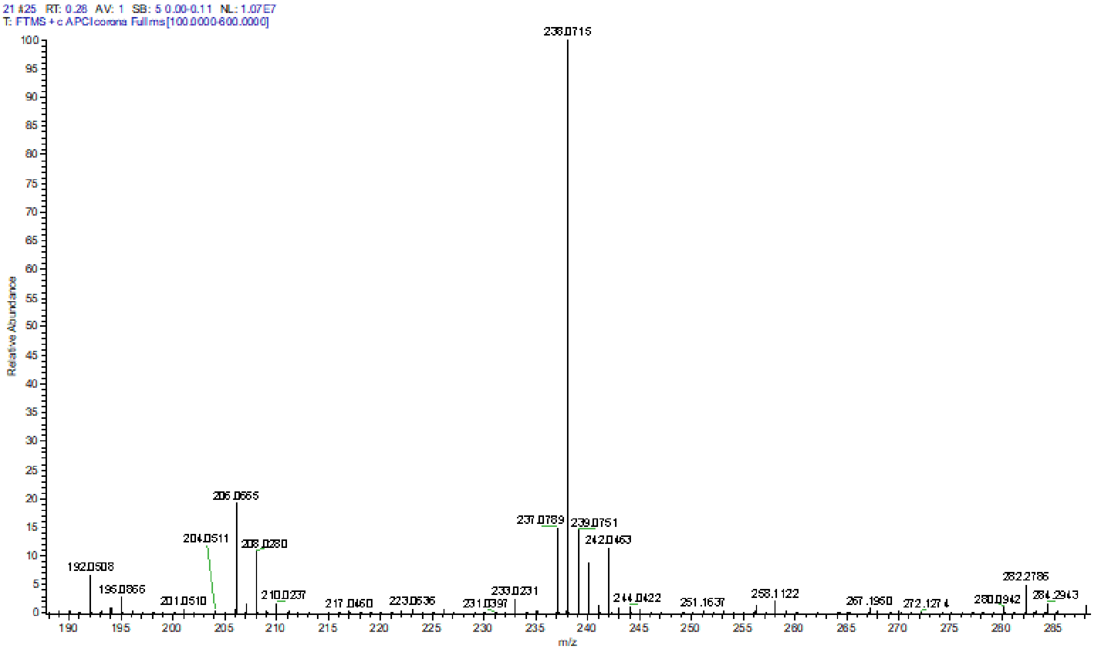

T: FTMS + c APCI corona Full ms [100.0000-600.0000]
238.0715
206.0665
237.0789
239.0751
242.0463
Relative Abundance
m/z

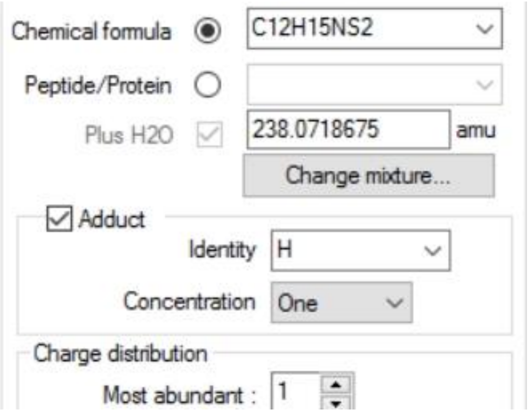

Chemical formula
C12H15NS2
Peptide/Protein
Plus H2O
238.0718675
amu
Change mixture...
Adduct
Identity
H
Concentration
One
Charge distribution
Most abundant :
1

4

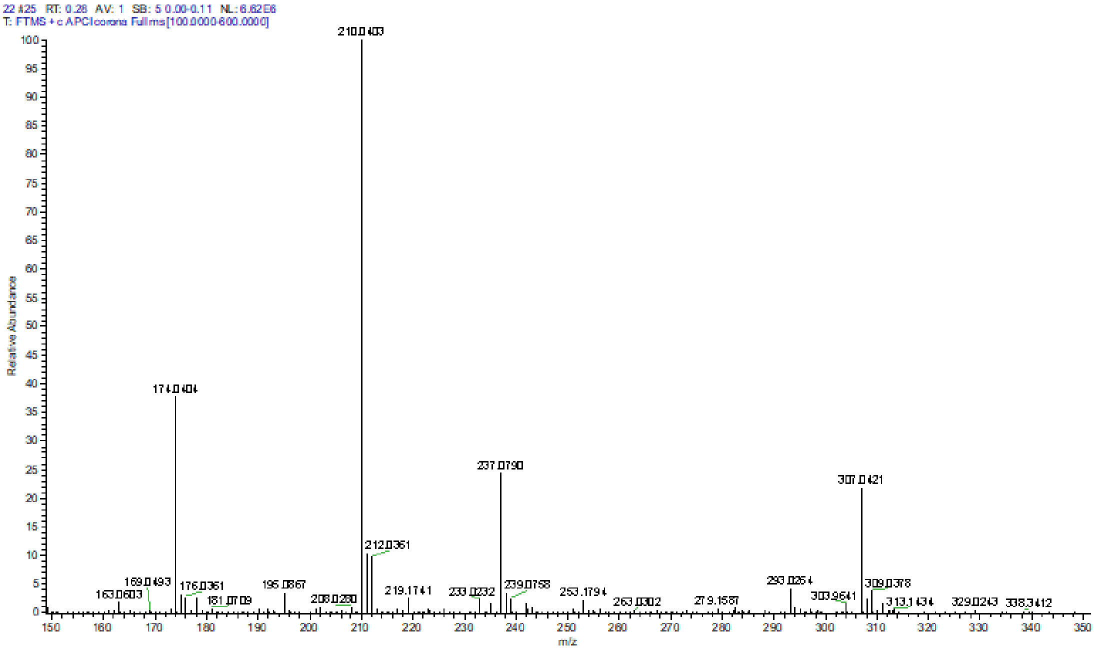

22 #25 RT: 0.28 AV: 1 SB: 5 0.00-0.11 NL: 6.62E6
T: FTMS + c APCI corona Full ms [100.0000-600.0000]
Relative Abundance
m/z
210.0403
174.0404
237.0790
307.0421
212.0351

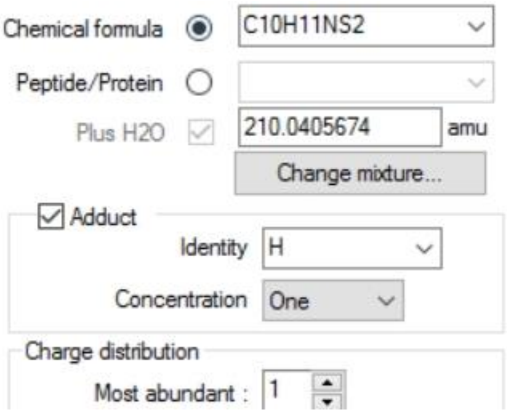

Chemical formula
C10H11NS2
Peptide/Protein
Plus H2O
210.0405674
amu
Change mixture...
Adduct
Identity
H
Concentration
One
Charge distribution
Most abundant :
1

5a

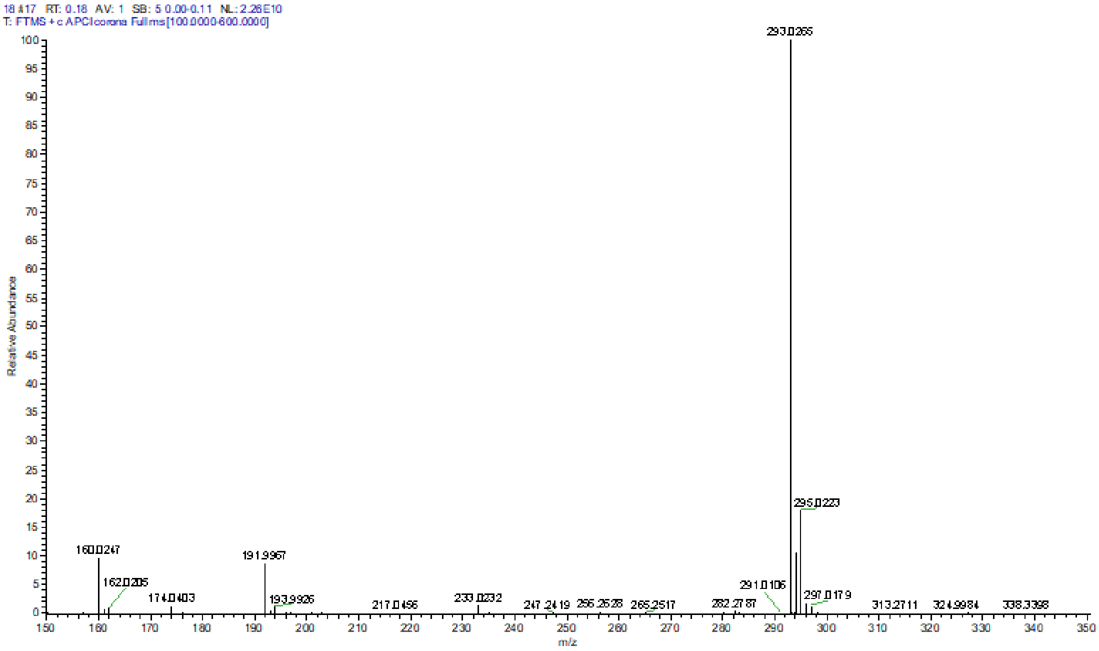
18 #17 RT: 0.18 AV: 1 SB: 5 0.00-0.11 NL: 2.28E10
T: FTMS + c APCI corona Full ms [100.0000-600.0000]
Relative Abundance
m/z
293.0265
295.0223
160.0247
191.9967
162.0205
174.0403
193.9926
217.0456
233.0232
291.0106
297.0179
313.2711
324.9984
338.3398

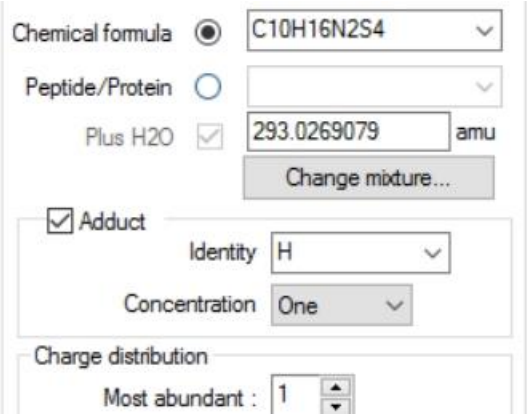
Chemical formula
C10H16N2S4
Peptide/Protein
Plus H2O
293.0269079
amu
Change mixture...
Adduct
Identity
H
Concentration
One
Charge distribution
Most abundant :
1

5b

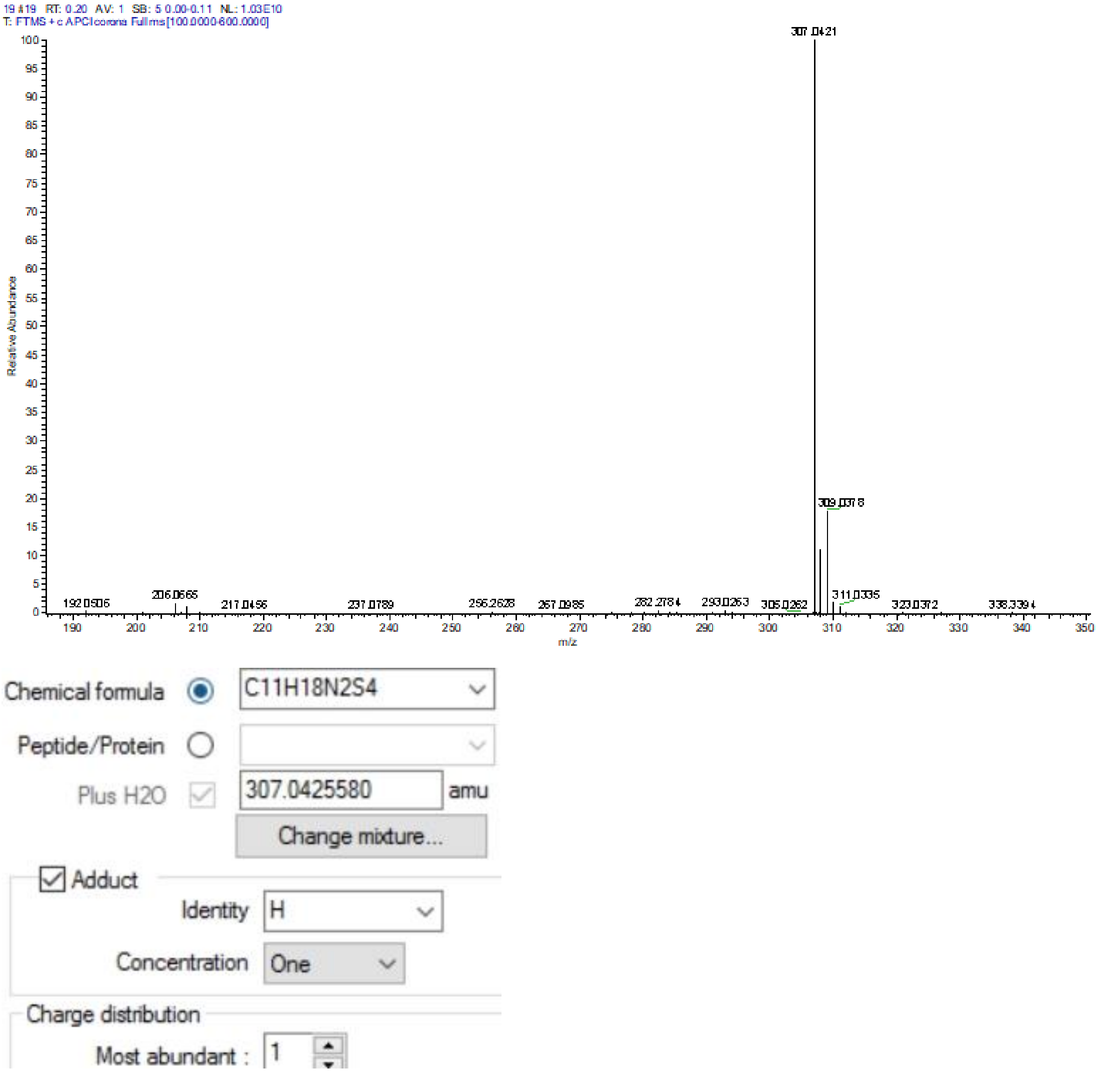

19 #19 RT: 0.20 AV: 1 SB: 5 0.00-0.11 NL: 1.03E10
T: FTMS + c APCI corona Full ms [100.0000-600.0000]
307.0421
309.0378
311.0335
Relative Abundance
m/z
Chemical formula
C11H18N2S4
Peptide/Protein
Plus H2O
307.0425580
amu
Change mixture...
Adduct
Identity
H
Concentration
One
Charge distribution
Most abundant :
1

5c

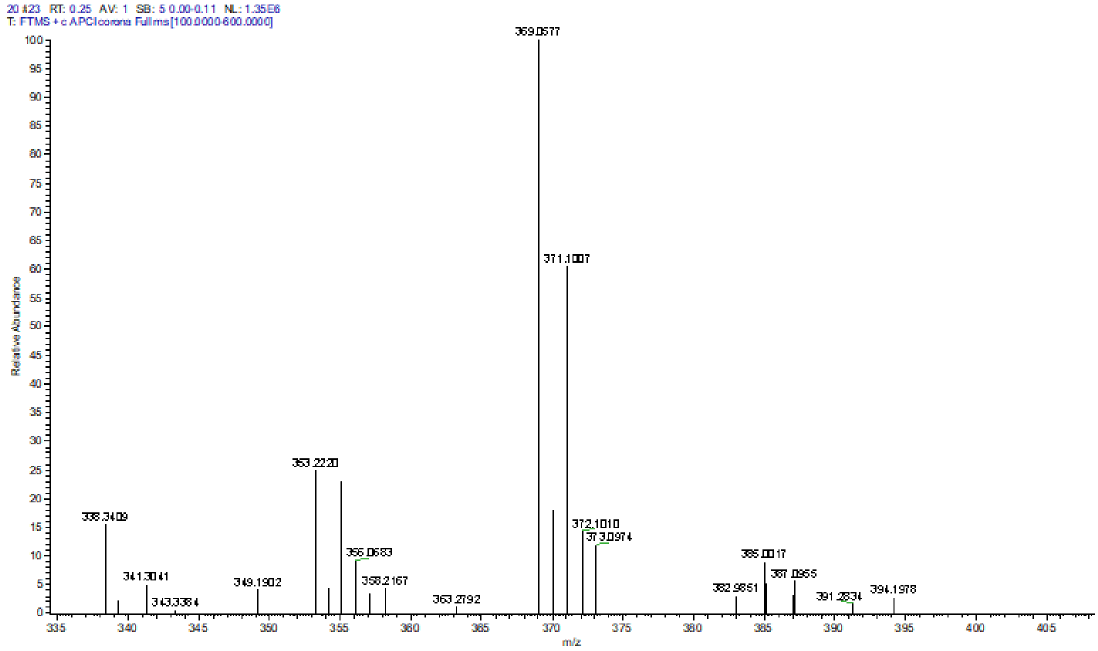

T: FTMS + c APCI corona Full ms [100.0000-600.0000]
Relative Abundance
m/z
369.0577
371.1007
353.2220
338.3409
372.1010
373.0974
356.0583
358.2167
341.3041
343.3384
349.1902
363.2792
385.0017
387.0955
382.9851
391.2834
394.1978

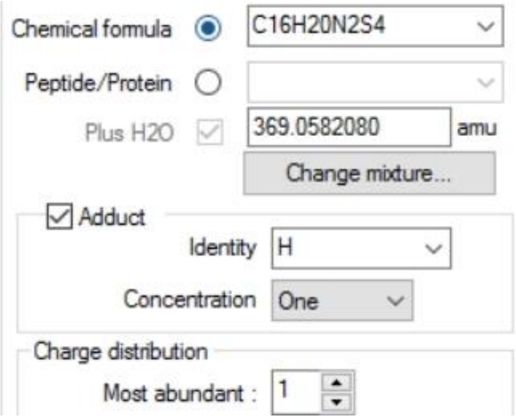

Chemical formula
C16H20N2S4
Peptide/Protein
Plus H2O
369.0582080
amu
Change mixture...
Adduct
Identity
H
Concentration
One
Charge distribution
Most abundant :
1
